# Annual (2024) taxonomic update of RNA-directed RNA polymerase-encoding negative-sense RNA viruses (realm Riboviria: kingdom Orthornavirae: phylum Negarnaviricota)

**DOI:** 10.1099/jgv.0.002077

**Published:** 2025-06-13

**Authors:** Jens H. Kuhn, Scott Adkins, Sergey V. Alkhovsky (Альховский Сергей Владимирович), Wenxia An (安雯霞), Tatjana Avšič-Županc, María A. Ayllón, Katarina Bačnik, Justin Bahl, Anne Balkema-Buschmann, Matthew J. Ballinger, Martin Beer, Nicolas Bejerman, Éric Bergeron, Nadine Biedenkopf, Carol D. Blair, Kim R. Blasdell, Steven B. Bradfute, Thomas Briese, Katherine Brown, Paul A. Brown, Ursula J. Buchholz, Michael J. Buchmeier, Alexander Bukreyev, Felicity Burt, Charles H. Calisher, Sten Calvelage, Mengji Cao (曹孟籍), Inmaculada Casas, Camila Chabi-Jesus, Kartik Chandran, Rémi N. Charrel, Anya Crane, Laura N. Cuypers, Elena Dal Bó, Juan Carlos de la Torre, William M. de Souza, Rik L. de Swart, Humberto J. Debat, Nolwenn M. Dheilly, Nicholas Di Paola, Francesco Di Serio, Ralf G. Dietzgen, Michele Digiaro, J. Felix Drexler, W. Paul Duprex, Ralf Dürrwald, Andrew J. Easton, Toufic Elbeaino, Koray Ergünay, Mirette I.Y. Eshak, Guozhong Feng (冯国忠), Andrew E. Firth, Anthony R. Fooks, Pierre B.H. Formenty, Juliana Freitas-Astúa, Conrad M. Freuling, Tuija Gadd, Selma Gago-Zachert, María Laura García, Adolfo García-Sastre, Aura R. Garrison, Tony L. Goldberg, Jean-Paul J. Gonzalez, Joëlle Goüy de Bellocq, Anthony Griffiths, Martin H. Groschup, Sophie Gryseels, Ion Gutiérrez-Aguirre, Stephan Günther, John Hammond, Jussi Hepojoki, Masayuki Horie (堀江真行), Adam J. Hume, Timothy H. Hyndman, Dirk Höper, Dàohóng Jiāng (姜道宏), Sandra Junglen, Boris Klempa, Jonas Klingström, Hideki Kondō (近藤秀樹), Eugene V. Koonin, Mart Krupovic, Kenji Kubota (久保田健嗣), Gael Kurath, Denis Kutnjak, Lies Laenen, Amy J. Lambert, Benhur Lee, Chengyu Li (李呈宇), Jiànróng Lǐ (李建荣), Jun-Min Li (李俊敏), Igor S. Lukashevich, Piet Maes, Marco Marklewitz, Sergio H. Marshall, Shin-Yi L. Marzano, John W. McCauley, Nataša Mehle, Ali Mirazimi, Toshiyuki Morikawa (守川俊幸), Elke Mühlberger, Thomas Müller, Rayapati Naidu, Tomohide Natsuaki (夏秋知英), Beatriz Navarro, José A. Navarro, Yutaro Neriya (煉谷裕太朗), Sergey V. Netesov (Нетёсов Сергей Викторович), Vittorio M. Nicoloso, Gabriele Neumann, Tiina Nokireki, Norbert Nowotny, Márcio R.T. Nunes, Francisco M. Ochoa-Corona, Gustavo Palacios, Vicente Pallás, Anna Papa (Άννα Παπά), Sofia Paraskevopoulou (Σοφία Παρασκευοπούλου), Colin R. Parrish, Alex Pauvolid-Corrêa, Anja Pecman, Daniel R. Pérez, Florian Pfaff, Richard K. Plemper, Thomas S. Postler, Sheli R. Radoshitzky, Pedro L. Ramos-González, Maja Ravnikar, Renato O. Resende, Gábor Reuter, Carina A. Reyes, Mark Paul Selda Rivarez, Víctor Romanowski, Dennis Rubbenstroth, Luisa Rubino, Jonathan A. Runstadler, Ana Ruiz-Padilla, Sead Sabanadzovic, Maria S. Salvato, Takahide Sasaya (笹谷孝英)I, Connie S. Schmaljohn, Heike Schmidt-Posthaus, Martin Schwemmle, Gabrijel Seljak, Torsten Seuberlich, Mang Shi (施莽), Yoshifumi Shimomoto (下元祥史), Peter Simmonds, Manuela Sironi, Donald B. Smith, Sophie Smither, Jin-Won Song (송진원), Kirsten M. Spann, Jessica R. Spengler, Mark D. Stenglein, Ayato Takada (高田礼人), Chika Takemura, Niina Tammiranta, Robert B. Tesh, Natalie J. Thornburg, Nicole D. Tischler, Yasuhiro Tomitaka (冨髙保弘), Keizō Tomonaga (朝長啓造), Noël Tordo, Massimo Turina, Ioannis E. Tzanetakis (Ιωάννης Ε. Τζανετάκης), Anna Maria Vaira, Bernadette van den Hoogen, Bert Vanmechelen, Nikos Vasilakis (Νίκος Βασιλάκης), Martin Verbeek, Susanne von Bargen, Ana Vučurović, Jiro Wada (和田治郎), Victoria Wahl, Peter J. Walker, Fei Wang (王飞), Anna E. Whitfield, John V. Williams, Yuri I. Wolf, Hironobu Yanagisawa (栁澤広宣), Caixia Yang (杨彩霞), Gongyin Ye (叶恭银), Mei Chun Yu (于美春), F. Murilo Zerbini, Song Zhang, Arnfinn Lodden Økland, Holly R. Hughes

**Affiliations:** 1 Integrated Research Facility at Fort Detrick (IRF-Frederick), National Institute of Allergy and Infectious Diseases (NIAID), National Institutes of Health (NIH), Fort Detrick, Frederick, MD, USA; 2 U.S. Horticultural Research Laboratory, Agricultural Research Service, U.S. Department of Agriculture, Fort Pierce, FL, USA; 3 D.I. Ivanovsky Institute of Virology of N.F. Gamaleya National Center on Epidemiology and Microbiology of Ministry of Health of Russian Federation, Moscow, Russia; 4 Liaoning Key Laboratory of Urban Integrated Pest Management and Ecological Security, College of Life Science and Engineering, Shenyang University, Dadong, Shenyang, Liaoning, PR China; 5 Institute of Microbiology and Immunology, Faculty of Medicine, University of Ljubljana, Ljubljana, Slovenia; 6 Centro de Biotecnología y Genómica de Plantas, Universidad Politécnica de Madrid (UPM)—Instituto Nacional de Investigación y Tecnología Agraria y Alimentaria (INIA/CSIC), Campus de Montegancedo, Pozuelo de Alarcón, Madrid, Spain; 7 Departamento de Biotecnología-Biología Vegetal, Escuela Técnica Superior de Ingeniería Agronómica, Alimentaria y de Biosistemas, Universidad Politécnica de Madrid (UPM), Madrid, Spain; 8 Department of Biotechnology and Systems Biology, National Institute of Biology, Ljubljana, Slovenia; 9 Department of Infectious Diseases, College of Veterinary Medicine, University of Georgia, Athens, GA, USA; 10 Department of Epidemiology and Biostatistics, College of Public Health, University of Georgia, Athens, GA, USA; 11 Institute of Novel and Emerging Infectious Diseases, Friedrich-Loeffler-Institut, Greifswald-Insel Riems, Greifswald, Germany; 12 Department of Biological Sciences, Mississippi State University, Mississippi State, Starkville, MS, USA; 13 Institute of Diagnostic Virology, Friedrich-Loeffler-Institut, Greifswald-Insel Riems, Germany; 14 UFyMA, INTA-CONICET, Córdoba, Argentina; 15 Viral Special Pathogens Branch, Division of High-Consequence Pathogens and Pathology, Centers for Disease Control and Prevention, Atlanta, GA, USA; 16 Institute of Virology, Philipps-University Marburg, Marburg, Germany; 17 Department of Microbiology, Immunology and Pathology, Colorado State University, Fort Collins, CO, USA; 18 Australian Centre for Disease Preparedness, Commonwealth Scientific and Industrial Research Organisation (CSIRO), Geelong, VIC, Australia; 19 University of New Mexico Health Sciences Center, Albuquerque, NM, USA; 20 Center for Infection and Immunity and Department of Epidemiology, Mailman School of Public Health, Columbia University, New York, NY, USA; 21 Department of Pathology, University of Cambridge, Cambridge, UK; 22 Laboratory of Ploufragan-Plouzané-Niort, French Agency for Food, Environmental and Occupational Health Safety (ANSES), Ploufragan, France; 23 RNA Viruses Section, Laboratory of Infectious Diseases, National Institute of Allergy and Infectious Diseases (NIAID), National Institutes of Health (NIH), Bethesda, MD, USA; 24 Department of Molecular Biology and Biochemistry, University of California, Irvine, CA, USA; 25 The University of Texas Medical Branch at Galveston, Galveston, TX, USA; 26 Division of Virology, National Health Laboratory Service and Division of Virology, University of the Free State, Bloemfontein, South Africa; 27 Colorado State University, Fort Collins, CO, USA; 28 National Citrus Engineering and Technology Research Center, Citrus Research Institute, Southwest University, Beibei, Chongqing, PR China; 29 Respiratory Virus and Influenza Unit, National Microbiology Center, Instituto de Salud Carlos III, Madrid, Spain; 30 Departamento de Fitopatologia e Nematologia, Escola Superior de Agricultura Luiz de Queiroz (ESALQ), Universidade de São Paulo, Piracicaba, São Paulo, Brazil; 31 Department of Microbiology and Immunology, Albert Einstein College of Medicine, Bronx, NY, USA; 32 Unite des Virus Emergents (Aix-Marseille Univ, Universita di Corsica, IRD 190, Inserm 1207, IRBA), Marseille, France; 33 University of Antwerp, Antwerp, Belgium; 34 Royal Belgian Institute of Natural Sciences, Brussels, Belgium; 35 CIDEFI, Facultad de Ciencias Agrarias y Forestales, Universidad de La Plata, La Plata, Argentina; 36 Department of Immunology and Microbiology IMM-6, The Scripps Research Institute, La Jolla, CA, USA; 37 Department of Microbiology, Immunology and Molecular Genetics, University of Kentucky, Lexington, KY, USA; 38 Department of Virology, Wageningen Bioveterinary Research, Lelystad, Netherlands; 39 Instituto de Patología Vegetal, Centro de Investigaciones Agropecuarias, Instituto Nacional de Tecnología Agropecuaria (IPAVE-CIAP-INTA), Córdoba, Argentina; 40 Consejo Nacional de Investigaciones Científicas y Técnicas, Unidad de Fitopatología y Modelización Agrícola, Córdoba, Argentina; 41 Pathogen Discovery Laboratory, Institut Pasteur, Paris, France; 42 U.S. Army Medical Research Institute of Infectious Diseases, Fort Detrick, Frederick, MD, USA; 43 Istituto per la Protezione Sostenibile delle Piante, Consiglio Nazionale delle Ricerche, Bari, Italy; 44 Queensland Alliance for Agriculture and Food Innovation, The University of Queensland, St. Lucia, QLD, Australia; 45 CIHEAM, Istituto Agronomico Mediterraneo di Bari, Valenzano, Italy; 46 Institute of Virology, Charité-Universitätsmedizin, corporate member of Freie Universität Berlin, Humboldt-Universität zu Berlin, Berlin, Germany; 47 School of Medicine, University of Pittsburgh, Pittsburgh, PA, USA; 48 Robert Koch Institut, Berlin, Germany; 49 School of Life Sciences, University of Warwick, Coventry, UK; 50 Walter Reed Biosystematics Unit (WRBU), Smithsonian Institution, Museum Support Center, Suitland, MD, USA; 51 One Health Branch, Walter Reed Army Institute of Research (WRAIR), Silver Spring, MD, USA; 52 Department of Entomology, Smithsonian Institution–National Museum of Natural History (NMNH), Washington, DC, USA; 53 China National Rice Research Institute, Hangzhou, PR China; 54 Animal and Plant Health Agency, Weybridge, Surrey, UK; 55 World Health Organization, Geneva, Switzerland; 56 Embrapa Cassava and Fruits, Cruz das Almas, Bahia, Brazil; 57 Institute of Molecular Virology and Cell Biology, Friedrich-Loeffler-Institut, Greifswald-Insel Riems, Germany; 58 Laboratory and Research Division, Finnish Food Authority, Helsinki, Finland; 59 Institute of Biochemistry and Biotechnology, Martin Luther University Halle-Wittenberg, Halle/Saale, Germany; 60 Instituto de Biotecnología y Biología Molecular, Facultad de Ciencias Exactas, CONICET UNLP, La Plata, Argentina; 61 Icahn School of Medicine at Mount Sinai, New York, NY, USA; 62 Department of Pathobiological Sciences, School of Veterinary Medicine, University of Wisconsin-Madison, Madison, WI, USA; 63 Department of Microbiology and Immunology, Division of Biomedical Graduate Research Organization, School of Medicine, Georgetown University, Washington, DC, USA; 64 Institute of Vertebrate Biology of the Czech Academy of Sciences, Brno, Czechia; 65 Department of Molecular Microbiology and Immunology, Bond Life Sciences Center, Laboratory for Infectious Disease Research, University of Missouri, Columbia, MO, USA; 66 Institute of Novel and Emerging Infectious Diseases, Friedrich-Loeffler-Institut, Greifswald-Insel Riems, Germany; 67 Department of Virology, WHO Collaborating Centre for Arboviruses and Hemorrhagic Fever Reference and Research, Bernhard-Nocht Institute for Tropical Medicine, Hamburg, Germany; 68 Floral and Nursery Plants Research Unit, U.S. National Arboretum, Washington, DC, USA; 69 Agricultural Research Service, U.S. Department of Agriculture, Beltsville, MD, USA; 70 Department of Virology, University of Helsinki, Medicum, Helsinki, Finland; 71 Institute of Veterinary Pathology, Vetsuisse Faculty, University of Zurich, Zurich, Switzerland; 72 Graduate School of Veterinary Science, Osaka Metropolitan University, Izumisano, Osaka, Japan; 73 International Research Center for Infectious Diseases, Osaka Metropolitan University, Izumisano, Osaka, Japan; 74 Department of Virology, Immunology and Microbiology, Chobanian and Avedisian School of Medicine, National Emerging Infectious Diseases Laboratories, Boston University, Boston, MA, USA; 75 School of Veterinary Medicine, Murdoch University, Murdoch, WA, Australia; 76 State Key Laboratory of Agricultural Microbiology, Huazhong Agricultural University, Wuhan, Hubei Province, PR China; 77 Institute of Virology, Charité-Universitätsmedizin Berlin, Freie Universität Berlin, Humboldt-Universität zu Berlin, and Berlin Institute of Health, Berlin, Germany; 78 Biomedical Research Center, Institute of Virology, Slovak Academy of Sciences, Bratislava, Slovakia; 79 Department of Biomedical and Clinical Sciences, Linköping University, Linköping, Sweden; 80 Institute of Plant Science and Resources, Okayama University, Kurashiki, Japan; 81 Computational Biology Branch, Division of Intramural Research, National Library of Medicine (NLM), National Institutes of Health (NIH), Bethesda, MD, USA; 82 Institut Pasteur, Université Paris Cité, CNRS UMR6047, Archaeal Virology Unit, Paris, France; 83 Institute for Plant Protection, National Agriculture and Food Research Organization (NARO), Tsukuba, Ibaraki, Japan; 84 Western Fisheries Research Center, U.S. Geological Survey, Seattle, WA, USA; 85 Department of Laboratory Medicine, University Hospitals Leuven, Leuven, Belgium; 86 Centers for Disease Control and Prevention, Fort Collins, CO, USA; 87 Department of Microbiology, Icahn School of Medicine at Mount Sinai, New York, NY, USA; 88 Department of Veterinary Biosciences, College of Veterinary Medicine, The Ohio State University, Columbus, OH, USA; 89 State Key Laboratory for Managing Biotic and Chemical Threats to the Quality and Safety of Agro-Products, Key Laboratory of Biotechnology in Plant Protection of Ministry of Agriculture and Zhejiang Province, Institute of Plant Virology, Ningbo University, Ningbo, PR China; 90 Department of Pharmacology and Toxicology, School of Medicine, and the Center for Predictive Medicine for Biodefense and Emerging Infectious Diseases, University of Louisville, Louisville, KY, USA; 91 Zoonotic Infectious Diseases Unit, Rega Institute, KU Leuven, Leuven, Belgium; 92 FIND, Geneva, Switzerland; 93 Instituto de Biología-Laboratorio de Genética Molecular-Pontificia Universidad Católica de Valparaíso, Campus Curauma, Valparaíso, Chile; 94 Agricultural Research Service, U. S. Department of Agriculture, Toledo, OH, USA; 95 Worldwide Influenza Centre, Francis Crick Institute, London, UK; 96 School for Viticulture and Enology, University of Nova Gorica, Vipava, Slovenia; 97 Folkhalsomyndigheten, Stockholm, Sweden; 98 Japan Plant Protection Association, Tokyo, Japan; 99 Department of Plant Pathology, Irrigated Agricultural Research and Extension Center, Washington State University, Prosser, WA, USA; 100 Utsunomiya University, Utsunomiya, Japan; 101 Instituto de Biología Molecular y Celular de Plantas, Universitat Politècnica de València-Consejo Superior de Investigaciones Científicas, Valencia, Spain; 102 School of Agriculture, Utsunomiya University, Utsunomiya, Japan; 103 Department of Pathobiological Sciences, Influenza Research Institute, University of Wisconsin-Madison, Madison, USA; 104 Institute of Virology, University of Veterinary Medicine Vienna, Vienna, Austria; 105 College of Medicine, Mohammed Bin Rashid University of Medicine and Health Sciences, Dubai Health, Dubai, UAE; 106 Laboratory of Molecular Biotechnology, Institute of Biological Sciences, Federal University of Pará State, Belem, Pará, Brazil; 107 Institute for Biosecurity and Microbial Forensics, Oklahoma State University, Stillwater, Oklahoma, USA; 108 Instituto de Biología Molecular y Celular de Plantas (IBMCP), Consejo Superior de Investigaciones Cientificas-Universitat Politècnica de Valencia, Valencia, Spain; 109 National Reference Centre for Arboviruses and Haemorrhagic Fever Viruses, Department of Microbiology, Medical School, Aristotle University of Thessaloniki, Thessaloniki, Greece; 110 Bioinformatics and Systems Biology, Robert Koch Institute, Berlin, Germany; 111 College of Veterinary Medicine, Baker Institute for Animal Health, Cornell University, Ithaca, NY, USA; 112 Departamento de Veterinária, Universidade Federal de Viçosa, Viçosa, Minas Gerais, Brazil; 113 Department of Population Health, College of Veterinary Medicine, University of Georgia, Athens, GA, USA; 114 Center for Translational Antiviral Research, Georgia State University Institute for Biomedical Sciences, Atlanta, GA, USA; 115 Vaccine Design and Development Laboratory, International AIDS Vaccine Initiative, Jersey City, NJ, USA; 116 Division of Antivirals, Office of Infectious Diseases, Center for Drug Evaluation and Research, Food and Drug Administration, Silver Spring, MD, USA; 117 Instituto Biológico de São Paulo, São Paulo, Brazil; 118 Departamento de Biologia Celular, Universidade de Brasília, Brasília, Brazil; 119 Department of Medical Microbiology and Immunology, Medical School, University of Pécs, Pécs, Hungary; 120 Instituto de Biotecnología y Biología Molecular, CONICET-UNLP, Facultad de Ciencias Exactas, Universidad Nacional de La Plata, Buenos Aires, Argentina; 121 Faculty of Land and Food Systems, The University of British Columbia, Vancouver, Canada; 122 College of Agriculture and Agri-Industries, Caraga State University, Butuan City, Philippines; 123 Department of Infectious Disease & Global Health, Tufts University Cummings School of Veterinary Medicine, North Grafton, MA, USA; 124 Department of Agricultural Science and Plant Protection, Mississippi State University, Mississippi State, MS, USA; 125 Department of Veterinary Medicine, University of Maryland, College Park, MD, USA; 126 Strategic Planning Headquarters, National Agriculture and Food Research Organization, Tsukuba, Japan; 127 Institute for Fish and Wildlife Health, University of Bern, Bern, Switzerland; 128 Faculty of Medicine, University Medical Center-University Freiburg, Freiburg, Germany; 129 University of Bern, Bern, Switzerland; 130 Sun Yat-sen University, Shenzhen, PR China; 131 Kochi Agricultural Research Center, Nankoku, Kochi, Japan; 132 Nuffield Department of Medicine, University of Oxford, Oxford, UK; 133 School of Medicine and Surgery, University of Milano-Bicocca, Monza, Italy; 134 CBR Division, Dstl Porton Down, Salisbury, Wiltshire, UK; 135 Department of Microbiology, College of Medicine, Korea University, Seoul, Republic of Korea; 136 School of Biomedical Sciences, Faculty of Health, Queensland University of Technology, Brisbane, QLD, Australia; 137 Department of Microbiology, Immunology, and Pathology, College of Veterinary Medicine and Biomedical Sciences, Colorado State University, Fort Collins, CO, USA; 138 Division of Global Epidemiology, International Institute for Zoonosis Control, Hokkaido University, Sapporo, Japan; 139 Kochi Agricultural Research Center, Nankoku, Japan; 140 Centers for Disease Control and Prevention, Atlanta, GA, USA; 141 Laboratorio de Virología Molecular, Centro Ciencia & Vida, Fundación Ciencia & Vida and Escuela de Bioquímica, Facultad de Medicina y Ciencia, Universidad San Sebastián, Santiago, Chile; 142 Institute for Plant Protection, National Agriculture and Food Research Organization, Tsukuba, Japan; 143 Institute for Life and Medical Sciences (LiMe), Kyoto University, Kyoto, Japan; 144 Institut Pasteur de Guinée, Conakry, Guinea; 145 Institute for Sustainable Plant Protection, National Research Council of Italy (IPSP-CNR), Torino, Italy; 146 Department of Entomology and Plant Pathology, Division of Agriculture, University of Arkansas System, Fayetteville, AR, USA; 147 Department of Viroscience, Erasmus MC, University Medical Centre Rotterdam, Rotterdam, Netherlands; 148 VirusBank Platform, Department of Microbiology, Immunology and Transplantation, KU Leuven, Leuven, Belgium; 149 Biointeractions and Plant Health, Wageningen University and Research, Wageningen, Netherlands; 150 Division Phytomedicine, Faculty of Life Sciences, Humboldt-Universität zu Berlin, Berlin, Germany; 151 National Biodefense Analysis and Countermeasures Center, Fort Detrick, Frederick, MD, USA; 152 School of Chemistry and Molecular Biosciences, University of Queensland, St. Lucia, QLD, Australia; 153 Key Laboratory of Special Pathogens and Biosafety, Wuhan Institute of Virology, Chinese Academy of Sciences, Wuhan, Hubei, PR China; 154 Department of Entomology and Plant Pathology, North Carolina State University, Raleigh, NC, USA; 155 School of Medicine and Public Health, University of Wisconsin-Madison, Madison, WI, USA; 156 Yokohama Plant Protection Station, Yokohama, Kanagawa, Japan; 157 Institute of Insect Sciences, Zhejiang University, Hangzhou, PR China; 158 Department de Fitopatologia/BIOAGRO, Universidade Federal de Viçosa, Viçosa, Minas Gerais, Brazil; 159 Pharmaq Analytiq, Bergen, Norway

**Keywords:** *Aliusviridae*, *Arenaviridae*, articulaviral, *Articulavirales*, *Artoviridae*, *Aspiviridae*, *Bornaviridae*, bunyaviral, *Bunyavirales*, *Bunyaviricetes*, bunyavirus, *Crepuscuviridae*, *Discoviridae*, *Elliovirales*, *Filoviridae*, *Fimoviridae*, *Goujianvirales*, *Hantaviridae*, *Hareavirales*, International Committee on Taxonomy of Viruses (ICTV), jingchuviral, *Jingchuvirales*, *Konkoviridae*, *Lispiviridae*, megaclassification, megataxonomy, mononegaviral, *Mononegavirales*, muviral, *Muvirales*, *Mymonaviridae*, *Myriaviridae*, naedreviral, *Naedrevirales*, *Nairoviridae*, *Natareviridae*, negarnaviricot, *Negarnaviricota*, *Nyamiviridae*, *Orthomyxoviridae*, *Orthornavirae*, *Paramyxoviridae*, *Peribunyaviridae*, *Phasmaviridae*, *Phenuiviridae*, *Pneumoviridae*, *Rhabdoviridae*, *Riboviria*, *Sunviridae*, *Tenuivirus*, *Tosoviridae*, *Tospoviridae*, *Tulasviridae*, virus classification, virus nomenclature, virus taxonomy

## Abstract

In April 2024, following the annual International Committee on Taxonomy of Viruses (ICTV) ratification vote on newly proposed taxa, the phylum *Negarnaviricota* was expanded by 1 new order, 1 new family, 6 new subfamilies, 34 new genera and 270 new species. One class, two orders and six species were renamed. Seven families and 12 genera were moved; ten species were renamed and moved; and nine species were abolished. This article presents the updated taxonomy of *Negarnaviricota* as currently accepted by the ICTV, providing an essential annual update on the classification of members of this phylum that deepen understandings of their evolution, and supports critical public health measures for virus identification and tracking.

## Introduction

Phylum *Negarnaviricota* was established in 2018 by the International Committee on Taxonomy of Viruses (ICTV) for negative-sense RNA viruses encoding evolutionarily related RNA-directed RNA polymerases (RdRPs). The phylum includes two subphyla, *Haploviricotina* and *Polyploviricotina*, for negative-sense RNA viruses that encode RdRPs with or without mRNA capping activity, respectively [1,4]. The phylum was last updated in 2023 [5]. Here, we present the changes that were proposed to the phylum via official taxonomic proposals (TaxoProps) in 2023 and accepted by the ICTV in April 2024 [6]. These changes are now part of the official ICTV taxonomy [7,8]. Annual updates to virus taxonomy ensure that the classification systems remain responsive to new discoveries and reflect the latest research, facilitating a comprehensive approach to virus tracking and study.

## Taxonomic changes in subphylum *Haploviricotina*

### Class *Milneviricetes*, order *Serpentovirales*

Order *Serpentovirales* was renamed *Naedrevirales* to prevent confusion with pisoniviricete *Serpentovirinae* (TaxoProp 2023.007P.A.Serpentovirales_1nord_rename).

#### Family *Aspiviridae*

Genus *Ophiovirus* was expanded by one new species, *Ophiovirus capsici*, for pepper chlorosis associated virus (PepCaV) discovered by high-throughput sequencing (HTS) in pepper (*Capsicum annuum* L.) sampled in Kōchi Prefecture (高知県), Japan [9] (TaxoProp 2023.008P.A.Aspiviridae_1nsp).

### Class *Monjiviricetes*, order *Mononegavirales*

#### Family *Bornaviridae*

Genus *Cartilovirus* was created to include one new species, *Cartilovirus plani*, for little skate bornavirus (LSBV) discovered in a Sequence Read Archive (SRA) of little skates [rajid *Leucoraja erinacea* (Mitchill, 1825)] [10] (TaxoProp 2023.005M.A.Bornaviridae_1ngen_3nsp).

Genus *Cultervirus* was expanded by two new species, *Cultervirus electrophori* and *Cultervirus inflati*, for electric eel bornavirus (EEBV) discovered in an SRA of electric eel [gymnotid *Electrophorus electricus* (Linnaeus, 1766)] and finepatterned puffer bornavirus (FPBV) discovered in an SRA of finepatterned puffers [tetraodontid *Takifugu poecilonotus* (Temminck & Schlegel, 1850)], respectively [10] (TaxoProp 2023.005M.A.Bornaviridae_1ngen_3nsp).

#### Family *Filoviridae*

The family was expanded by one new genus, *Loebevirus*, including one new species, *Loebevirus percae*, for Lötschberg virus (LTBV) discovered by HTS in European perch (*Perca fluviatilis* Linnaeus, 1758) [11] (TaxoProp 2023.025M.Filoviridae_1 ng_1nsp).

#### Family *Lispiviridae*

Genus *Birfecvirus* was created to include one new species, *Birfecvirus tibetense*, for arlivirus sp. XZN142933 (ALV) discovered by HTS in bird faeces sampled in Tibet, China [12] (TaxoProp 2023.011M.A.Lispiviridae_1ngen_4nsp). The abbreviation ALV is changed here to ALV33 to distinguish this virus from arlivirus sp. virus (ALV) of species *Avesvirus sinense*.

Genus *Anicalvirus* was expanded by one new species, *Anicalvirus hesdarense*, for hymenopteran arli-related virus OKIAV100 (HARV-100) discovered by HTS in parasitoid wasps (orussid *Orussus unicolor* Latreille, 1812) sampled in Darmstadt, Hesse (Hessen), Germany [13] (TaxoProp 2023.011M.A.Lispiviridae_1ngen_4nsp).

Genus *Copasivirus* was expanded by one new species, *Copasivirus cattienense*, for Cát Tiên Hospitalitermes lispi-like virus (CHLV) discovered by HTS in termites (nasutitermitinine *Hospitalitermes bicolor* Holmgren, 1912) in Cát Tiên National Park (Vườn quốc gia Cát Tiên), Vietnam [14] (TaxoProp 2023.011M.A.Lispiviridae_1ngen_4nsp).

Genus *Ganiavirus* was expanded by one new species, *Ganiavirus fuyunense*, for Fùyùn tick virus 1 (FTV1) discovered by HTS in unspecified ticks sampled in Fùyùn County (富蕴县), Xīnjiāng Uygur Autonomous Region (新疆维吾尔自治区), China [15] (TaxoProp 2023.011M.A.Lispiviridae_1ngen_4nsp).

#### Family *Mymonaviridae*

Genus *Penicillimonavirus* was expanded by one new species, *Penicillimonavirus cercosporae*, for Cercospora beticola negative-stranded virus 3 (CbNSV3) discovered by metagenomics in dothideomycete fungi [mycosphaerellaceaen *Cercospora beticola* Sacc. (1876)] sampled in China [16] (TaxoProp 2023.016M.A.Mymonaviridae_11nsp).

Genus *Phyllomonavirus* was expanded by one new species, *Phyllomonavirus culicis*, for XiangYun mono-chu-like virus 7 discovered by metagenomics in mosquitoes (culicid *Culex pipiens* Linnaeus, 1758) sampled in Yúnnán Province (云南省), China (unpublished; GenBank #OL700132) (TaxoProp 2023.016M.A.Mymonaviridae_11nsp). XiangYun mono-chu-like virus 7 is decided here to be abbreviated XYmclV7.

Genus *Plasmopamonavirus* was expanded by two new species, *Plasmopamonavirus alphatrichodermae* and *Plasmopamonavirus betatrichodermae*, for Trichoderma harzianum mononegaviruses 1 (ThMV1) and 2 (ThMV2) discovered by metagenomics in sordariomycete fungi [hypocreaceaen *Trichoderma harzianum* Rifai (1969)] in an Italian collection, respectively [17] (TaxoProp 2023.016M.A.Mymonaviridae_11nsp).

Genus *Sclerotimonavirus* was expanded by seven new species (TaxoProp 2023.016M.A.Mymonaviridae_11nsp):

- *Sclerotimonavirus alphaalternariae* for Alternaria dianthicola negative-stranded RNA virus 1 (AdNSRV1) discovered by metagenomics in dothideomycete fungi [pleosporaceaen *Alternaria dianthicola* Neerg. (1945)] sampled in Húnán Province (湖南省), China [18];
- *Sclerotimonavirus prolifusarii* for Fusarium proliferatum mymonavirus 1 discovered by metagenomics in sordariomycete fungi [nectriaceaen *Fusarium proliferatum* (Matsush.) Nirenberg ex Gerlach & Nirenberg (1976)] sampled in Italy (unpublished; GenBank #OK524200). Fusarium proliferatum mymonavirus 1 is decided here to be abbreviated FpMV1;
- *Sclerotimonavirus alpharhipicephali* for Zhāngzhōu mymon tick virus 3 discovered by metagenomics in brown dog ticks [ixodid *Rhipicephalus sanguineus* (Latreille, 1806)] sampled in Zhāngzhōu (漳州市), Fújiàn Province (福建省), China [19]; Zhāngzhōu mymon tick virus 3 is decided here to be abbreviated ZzMtV3;
- *Sclerotimonavirus betaalternariae* for Alternaria tenuissima negative-stranded RNA virus 2 (AtNSRV2) discovered by metagenomics in dothideomycete fungi [pleosporaceaen *Alternaria tenuissima* Samuel Paul Wiltshire (1933)] sampled in China [20];
- *Sclerotimonavirus betarhipicephali* for Sānyà mymon tick virus 1 discovered by metagenomics in brown dog ticks [ixodid *Rhipicephalus sanguineus* (Latreille, 1806)] sampled in Hǎinán Province (海南省), China [19]; Sānyà mymon tick virus 1 is decided here to be abbreviated SyMtV1;
- *Sclerotimonavirus ceratocystidis* for Sclerotimonavirus cacaofunestae (SVc) discovered by metagenomics in sordariomycete fungi [ceratocystidaceaen *Ceratocystis cacaofunesta* Engelbr. & T.C.Harr. (2005)] [21]; and
- *Sclerotimonavirus trichodermae* for Trichoderma harzianum negative-stranded virus 2 (ThNV2) discovered by metagenomics in sordariomycete fungi [hypocreaceaen *Trichoderma harzianum* Rifai (1969)] in an Italian collection [17].

#### Family *Nyamiviridae*

Genus *Berhavirus* was expanded by four new species (TaxoProp 2023.029M.Nyamiviridae_7nsp):

- *Berhavirus alphagirardiae* for girado virus 1 (GiV1), *Berhavirus betagirardiae* for girado virus 2 (GiV2) and *Berhavirus gammagirardiae* for girado virus 3 (GiV3), all discovered by mining of a transcriptome shotgun assembly (TSA) database, in a dugesiid triclad [*Girardia dorotocephala* (Woodworth, 1897)] [22] and
- *Berhavirus macrostomi* for macroli virus (MacrV), discovered by mining of a TSA database, in a flatworm (macrostomid *Macrostomum lignano* Ladurner, Schärer, Salvenmoser, & Rieger, 2005) [22].

Genus *Formivirus* was expanded by one new species, *Formivirus ixodis*, for Ixodes ricinus orinovirus-like virus 1 (IROV1), identified by HTS in castor bean ticks [ixodid *Ixodes ricinus* (Linnaeus, 1758)] sampled in Croatia [23].

Genus *Nyavirus* was expanded by one new species, *Nyavirus gerbillisci*, for Toure virus (TOUV) isolated from northern savanna gerbils [murid *Gerbilliscus kempi* (Wroughton, 1906)] sampled in Senegal [24] and unpublished (GenBank #OL774864) (TaxoProp 2023.029M.Nyamiviridae_7nsp).

Genus *Tapwovirus* was expanded by one new species, *Tapwovirus taeniae*, for taeniapi virus (TNPV), discovered by mining of a TSA database, in rabbit tapeworms (taeniid *Taenia pisiformis* Bloch 1780) [22].

#### Family *Paramyxoviridae*

Five new subfamilies were created (TaxoProp 2023.018M.Paramyxoviridae_reorg):

- *Feraresvirinae*, to harbour the established genera *Aquaparamyxovirus*, *Ferlavirus* and *Respirovirus*;
- *Glossavirinae*, *Ichthysvirinae* and *Skoliovirinae* to harbour the three previously unassigned genera *Cynoglossusvirus*, *Hoplichthysvirus* and *Scoliodonvirus,* respectively; and
- *Kamposvirinae*, to harbour a new genus, *Hippocavirus*, to include one new species, *Hippocavirus hippocampi*, for Hippocampus erectus paramyxovirus 1 (HePmV1) discovered by HTS in lined seahorses (syngnathid *Hippocampus erectus* Perry, 1810) sampled in Shāndōng Province (山东省), China [25].

##### Subfamily *Avulavirinae*

Genus *Metaavulavirus* was expanded by three new species:

- *Metaavulavirus bangorense* for ‘APMV-2/Finch/N.Ireland/Bangor/73’ isolated from an unspecified finch sampled in Bangor, Northern Ireland, UK [26] – here renamed avian paramyxovirus 26;
- *Metaavulavirus calidris* for ‘AMPV-6/red-necked stint/Japan/8KS0813/2008’ isolated from a red-necked stint [scolopacid *Calidris ruficollis* (Pallas, 1776)] sampled in Hokkaido (北海道), Japan [27] – here renamed avian paramyxovirus 24; and
- *Metaavulavirus procarduelis* for ‘APMV-2/Procarduelis nipalensis/China/Suiling/53/2013’ discovered by HTS in dark-breasted rosefinch [fringillid *Procarduelis nipalensis* (Hodgson, 1836)] sampled in Suílíng County (绥棱县), Hēilóngjiāng Province (黑龙江省), China (unpublished; GenBank #KT071755) [28] – here renamed avian paramyxovirus 27.

Genus *Orthoavulavirus* was expanded by two new species:

- *Orthoavulavirus arenariae* for avian paramyxovirus 23 (APMV23) isolated from a cloacal swab of a ruddy turnstone [scolopacid *Arenaria interpres* (Linnaeus, 1758)] sampled in FL, USA [28]; and
- *Orthoavulavirus taiwanense* for ‘APMV-12/Anseriformes/Taiwan/AHRI101/2015’ discovered in an unspecified anseriform bird sampled in Taiwan [29] – here renamed avian paramyxovirus 25 (APMV25).

Genus *Paraavulavirus* was expanded by one new species, *Paraavulavirus neophemae* for ‘APMV3/PKT/Netherland/449/75’ isolated from a parakeet (psittaculid *Neophema* sp.) sampled in the Netherlands [30,31] – here renamed avian paramyxovirus 28 (APMV28).

##### Subfamily *Feraresvirinae*

Genus *Respirovirus* was expanded by two new species:

- *Respirovirus henanense* for porcine parainfluenza virus 2 (PPIV2) discovered by HTS in domestic pigs sampled in Hénán Province (河南省), China [32] and
- *Respirovirus rupicaprae* for ‘respirovirus ChamoisRV/IT2014’ isolated from Alpine chamois [bovid *Rupicapra rupicapra rupicapra* (Linnaeus, 1758)] sampled in the northwestern Italian Alps [33] – here renamed chamois respirovirus (ChRPV).

##### Subfamily *Orthoparamyxovirinae*

Subfamily *Orthoparamyxovirinae* was expanded by five genera:

Genus *Bovinavirus* was created to include new species *Bovinavirus bovis* for Bulang virus (BulV) discovered by HTS in cattle (bovid *Bos taurus* Linnaeus, 1758) sampled in New South Wales, Australia [34].

Genus *Henipavirus* was expanded by one new species, *Henipavirus angavokelyense*, for Angavokely virus (AngV) discovered by HTS in Malagasy straw-coloured fruit bats (pteropodid *Eidolon dupreanum* Schlegel & Pollen, 1866) sampled in Angavokely near Antananarivo, Madagascar [35]. Ghana virus (GhV; species *Henipavirus ghanaense*) is here renamed Okum tree virus (OtrV).

Genus *Jeilongvirus* was expanded by 24 new species:

- *Jeilongvirus chaetodipodis* for ‘paramyxoviridae sp. isolate AB1’ discovered by HTS in Bailey’s pocket mice [heteromyid *Chaetodipus baileyi* (Merriam, 1894)] sampled in Arizona, USA [36] – here renamed chaeba virus (ChaBV);
- *Jeilongvirus eothenomysis* for Wǔfēng Eothenomys melanogaster jeilongvirus 1 (here decided to be abbreviated WfEmJV1) discovered by HTS in a Père David’s red-backed vole [cricetid *Eothenomys melanogaster* (A. Milne-Edwards, 1871)] sampled in Wǔfēng (五峰镇), Húběi Province (湖北省), China [37];
- *Jeilongvirus gueckedouense* for memana virus (MemaV) discovered by HTS in Natal mastomys (murid *Mastomys natalensis* Smith, 1834) sampled in Méliandou, Nzérékoré Region, Guinea [38];
- *Jeilongvirus lishuiense* for Lóngquán Niviventer fulvescens jeilongvirus 2 (here decided to be abbreviated LqNfJV2) discovered by HTS in Indomalayan niviventer [murid *Niviventer fulvescens* (Gray, 1847)] sampled in Lóngquán (龙泉市), Zhèjiāng Province (浙江省), China [37];
- *Jeilongvirus longquanense* for Lóngquán Niviventer fulvescens jeilongvirus 1 (here decided to be abbreviated LqNfJV1) discovered by HTS in Indomalayan niviventer [murid *Niviventer fulvescens* (Gray, 1847)] sampled in Lóngquán (龙泉市), Zhèjiāng Province (浙江省), China [37];
- *Jeilongvirus mastomysis* for ninomys virus (NinYsV) discovered by HTS in a harvest mouse [murid *Micromys minutus* (Pallas, 1771)] sampled in Ninove, East Flanders (Oost-Vlaanderen/Flandre-Orientale/Ostflandern), Belgium [39];
- *Jeilongvirus meliandouense* for Méliandou lophuromys virus discovered by HTS in rusty-bellied brush-furred rats [murid *Lophuromys sikapusi* (Temminck, 1853)] sampled in Méliandou, Nzérékoré Region, Guinea [38];
- *Jeilongvirus merionis* for gerbil paramyxovirus (GePV) discovered by HTS in Mongolian jirds [murid *Meriones unguiculatus* (Milne-Edwards, 1867)] sampled in Shaanxi/Shǎnxī Province (陕西省), China [40];
- *Jeilongvirus microti* for Ninove microtus virus (NiMiV) discovered by HTS in field voles [cricetid *Microtus agrestis* (Linnaeus, 1761)] sampled in Ninove, East Flanders (Oost-Vlaanderen/Flandre-Orientale/Ostflandern), Belgium [38];
- *Jeilongvirus neotomae* for ‘Paramyxoviridae sp. isolate AK5’ discovered by HTS in white-throated woodrats (cricetid *Neotoma albigula* Hartley, 1894) sampled in AZ, USA [36] – here renamed neotal virus (NeTV);
- *Jeilongvirus ninovense* for ninapo virus (NinaV) discovered by HTS in long-tailed field mice [murid *Apodemus sylvaticus* (Linnaeus, 1758)] sampled in Ninove, East Flanders (Oost-Vlaanderen/Flandre-Orientale/Ostflandern), Belgium [39];
- *Jeilongvirus niviventris* for Lóngquán Niviventer niviventer jeilongvirus 1 (here decided to be abbreviated LqNnJV1) discovered by HTS in a Himalayan niviventer [murid *Niviventer niviventer* (Hodgson, 1836)] sampled in Lóngquán (龙泉市), Zhèjiāng Province (浙江省), China [37];
- *Jeilongvirus nzerekorense* for Méliandou mastomys virus (MeMV) discovered by HTS in reddish-white mastomys [murid *Mastomys erythroleucus* (Temminck, 1853)] sampled in Méliandou, Nzérékoré Region, Guinea [38];
- *Jeilongvirus ochotonae* for Ochotona cansus jeilongvirus (here decided to be abbreviated OcJV) discovered by HTS in a Gansu pika (ochotonid *Ochotona cansus* Lyon, 1907) sampled in China [41];
- *Jeilongvirus oujiangense* for Wēnzhōu Rattus norvegicus jeilongvirus 1 (here decided to be abbreviated WzRnJV1) discovered by HTS in brown rats [murid *Rattus norvegicus* (Berkenhout, 1769)] sampled in Wēnzhōu (温州市), Zhèjiāng Province (浙江省), China [37];
- *Jeilongvirus pajuense* for Paju Apodemus paramyxovirus 1 (PAPV1) discovered by HTS in striped field mice [murid *Apodemus agrarius* (Pallas, 1771)] sampled in multiple provinces of South Korea [42];
- *Jeilongvirus praomysis* for Méliandou praomys virus (MePV) discovered by HTS in West African praomys [murid *Praomys rostratus* (Miller, 1900)] sampled in Méliandou, Nzérékoré Region, Guinea [38];
- *Jeilongvirus ratti* for Wēnzhōu Rattus losea jeilongvirus 2 (here decided to be abbreviated WzRlJV2) discovered by HTS in Losea rats [murid *Rattus losea* (R. Swinhoe, 1871)] sampled in Wēnzhōu (温州市), Zhèjiāng Province (浙江省), China [37];
- *Jeilongvirus sichuanense* for Eothenomys eva jeilongvirus (here decided to be abbreviated EeJV) discovered by HTS in Eva’s red-backed voles [cricetid *Caryomys eva* (Thomas, 1911)) sampled in Sìchuān Province (四川省), China [41];
- *Jeilongvirus taichungense* for Wǔfēng Rattus nitidus jeilongvirus 1 (here decided to be abbreviated WfRnJV1) discovered by HTS in a white-footed Indochinese rat [murid *Rattus nitidus* (Hodgson, 1845)] sampled in Wǔfēng (五峰镇), Húběi Province (湖北省), China [37];
- *Jeilongvirus typhlomysis* for Wǔfēng Typhlomys cinereus jeilongvirus 1 (here decided to be abbreviated WfTcJV1) discovered by HTS in a soft-furred tree mouse (platacanthomyid *Typhlomys cinereus* Milne-Edwards, 1877) sampled in Wǔfēng (五峰镇), Húběi Province (湖北省), China [37];
- *Jeilongvirus winnikense* for denotus virus (DenoV) discovered by HTS in common voles [cricetid *Microtus arvalis* (Pallas, 1778)] sampled in Ninove, East Flanders (Oost-Vlaanderen/Flandre-Orientale/Ostflandern), Belgium [39];
- *Jeilongvirus wufengense* for Wǔfēng Apodemus chevrieri jeilongvirus 1 (here decided to be abbreviated WfAcJV1) discovered by HTS in Chevrier’s field mice [murid *Apodemus chevrieri* (Milne-Edwards, 1868)] sampled in Wǔfēng (五峰镇), Húběi Province (湖北省), China [37]; and
- *Jeilongvirus yeoncheonense* for Paju Apodemus paramyxovirus 2 (PAPV2) discovered by HTS in striped field mice [murid *Apodemus agrarius* (Pallas, 1771)] sampled in multiple provinces of South Korea [42].

Species *Jeilongvirus anhuiense*, *Jeilongvirus felis* and *Jeilongvirus murinae* were abolished.

Genus *Morbillivirus* was expanded by three new species:

- *Morbillivirus myotis* for Myotis bat morbillivirus (MBaMV) discovered by HTS in a riparian myotis (vespertilionid *Myotis riparius* Handley, 1960) sampled in the Amazon, Brazil [43];
- *Morbillivirus phyllostomi* for Phyllostomus bat morbillivirus (here decided to be abbreviated PBaMV) discovered by HTS in a greater spear-nosed bat [phyllostomid *Phyllostomus hastatus* (Pallas, 1767)] sampled in Atlantic Forest (Mata Atlântica), Brazil [43]; and
- *Morbillivirus suis* for porcine morbillivirus (PoMV) discovered by HTS in domestic pigs (suid *Sus scrofa* Linnaeus, 1758) sampled in northern Mexico [44].

Genus *Narmovirus* was expanded by three new species:

- *Narmovirus meliandouense* for meleucus virus (meleV) discovered by HTS in reddish-white mastomys [murid *Mastomys erythroleucus* (Temminck, 1853)] sampled in Méliandou, Nzérékoré Region, Guinea [38];
- *Narmovirus microti* for denalis virus (DenaV) discovered by HTS in common voles [cricetid *Microtus arvalis* (Pallas, 1778)] sampled in Ninove, East Flanders (Oost-Vlaanderen/Flandre-Orientale/Ostflandern), Belgium [38]; and
- *Narmovirus ninovense* for denestis virus (DeneV) discovered by HTS in field voles [cricetid *Microtus agrestis* (Linnaeus, 1761)] sampled in Ninove, East Flanders (Oost-Vlaanderen/Flandre-Orientale/Ostflandern), Belgium [38].

Genus *Parahenipavirus* was created for species *Henipavirus mojiangense* (moved from genus *Henipavirus* and renamed as *Parahenipavirus mojiangense*) and ten new species:

- *Parahenipavirus chodsigoae* for Wǔfēng Chodsigoa smithii henipavirus 1 (here decided to be abbreviated WfCsHV1) discovered by HTS in a Smith’s shrew [soricid *Chodsigoa smithii* (Thomas, 1911)] sampled in Wǔfēng (五峰镇), Húběi Province (湖北省), China [37];
- *Parahenipavirus crocidurae* for Wǔfēng Crocidura attenuata henipavirus 1 (here decided to be abbreviated WfCaHV1) discovered by HTS in Asian grey shrews (soricid *Crocidura attenuata* Milne-Edwards, 1872) sampled in Wǔfēng (五峰镇), Húběi Province (湖北省), China [37];
- *Parahenipavirus daeryongense* for Daeryong virus (DARV) discovered by HTS in Asian lesser white-toothed shrews (soricid *Crocidura shantungensis* Miller, 1901) sampled in Gangwon Province (강원도), South Korea [45];
- *Parahenipavirus gamakense* for Gamak virus (GAKV) discovered by HTS in Ussuri white-toothed shrews [soricid *Crocidura lasiura* (Dobson, 1890)] sampled in Gangwon Province (강원도), Gyeonggi Province (경기도) and South Gyeongsang Province (경상남도), South Korea [45];
- *Parahenipavirus jingmenense* for Jīngmén Crocidura shantungensis henipavirus 1 (here decided to be abbreviated JmCsHV1) discovered by HTS in Asian lesser white-toothed shrews (soricid *Crocidura shantungensis* Miller, 1901) sampled in Jīngmén (荆门市), Húběi Province (湖北省), China [37];
- *Parahenipavirus langyaense* for Lángyá virus (LayV) isolated from humans sampled in historic Lángyá Commandery (琅琊郡), Shāndōng Province (山东省) and Hénán Province (河南省), China [46];
- *Parahenipavirus meliandouense* for melian virus (MeliV) discovered by HTS in a large-headed shrew (soricid *Crocidura grandiceps* Hutterer, 1983) sampled in Méliandou, Nzérékoré Region, Guinea [38];
- *Parahenipavirus soricis* for ninorex virus (NinExV) discovered by HTS in a Eurasian pygmy shrew (soricid *Sorex minutus* Linnaeus, 1766) sampled in Ninove, East Flanders (Oost-Vlaanderen/Flandre-Orientale/Ostflandern), Belgium [39];
- *Parahenipavirus wenzhouense* for Wēnzhōu Apodemus agrarius henipavirus 1 (here decided to be abbreviated WzAaHV1) discovered by HTS in striped field mice [murid *Apodemus agrarius* (Pallas, 1771)] sampled in Wēnzhōu (温州市), Zhèjiāng Province (浙江省), China [37]; and
- *Parahenipavirus winnikense* for denwin virus (DewV) discovered by HTS in greater white-toothed shrews [soricid *Crocidura russula* (Hermann, 1780)] sampled in Ninove, East Flanders (Oost-Vlaanderen/Flandre-Orientale/Ostflandern), Belgium [38].

Genus *Parajeilongvirus* was created for species *Jeilongvirus comorosense*, *Jeilongvirus erinacei*, *Jeilongvirus madagascarense* and *Jeilongvirus miniopteri* (moved from genus *Jeilongvirus* and renamed as *Parajeilongvirus comorosense*, *Parajeilongvirus erinacei*, *Parajeilongvirus madagascarense* and *Parajeilongvirus miniopterid*, respectively) and 14 new species:

- *Parajeilongvirus brazilense* for ‘Diaemus bat paramyxovirus isolate PREDICT/PBZ-2282’ discovered by HTS in a white-winged vampire bat (phyllostomid *Diaemus youngi* Jentink, 1893) sampled in Atlantic Forest (Mata Atlântica), Brazil [43] – here renamed Diaemus bat paramyxovirus 1 (DBPV1);
- *Parajeilongvirus carolliae* for Carollia bat paramyxovirus (here decided to be abbreviated CBPV) discovered by HTS in Seba’s short-tailed bats (phyllostomid *Carollia perspicillata* (Linnaeus, 1758)] sampled in the Amazon, Brazil [43];
- *Parajeilongvirus desmodi* for Boe virus (BOEV) discovered by HTS in a common vampire bat [phyllostomid *Desmodus rotundus* (Geoffroy, 1810)] sampled around Araçatuba, São Paulo, Brazil [47];
- *Parajeilongvirus diaemi* for ‘Diaemus bat paramyxovirus isolate PREDICT/PBZ-3205’ discovered by HTS in a white-winged vampire bat (phyllostomid *Diaemus youngi* Jentink, 1893) sampled in Atlantic Forest (Mata Atlântica), Brazil [43] – here renamed Diaemus bat paramyxovirus 2 (DBPV2);
- *Parajeilongvirus hainanense* for Hipposideros armiger paramyxovirus (HaParaV) discovered by HTS in great leaf-nosed bats [hipposiderid *Hipposideros armiger* (Hodgson, 1835)] sampled in Hǎinán Province (海南省), China [48];
- *Parajeilongvirus hipposideri* for Hipposideros bat paramyxovirus (here decided to be abbreviated HBPV) discovered by HTS in Cantor’s leaf-nosed bats (hipposiderid *Hipposideros galeritus* Cantor, 1846) sampled in State of Sabah (Negeri Sabah), Malaysia [43];
- *Parajeilongvirus hubeiense* for Jīngmén Miniopterus schreibersii paramyxovirus 1 (here decided to be abbreviated JmMsPV1) discovered by HTS in Schreibers’s long-fingered bats [miniopterid *Miniopterus schreibersii* (Kuhl, 1817)] sampled in Jīngmén (荆门市), Húběi Province (湖北省), China [37];
- *Parajeilongvirus jingmenense* for Jīngmén Myotis davidii paramyxovirus 1 (here decided to be abbreviated JmMdPV1) discovered by HTS in a David’s myotis (vespertilionid *Myotis davidii* Peters, 1869) sampled in Jīngmén (荆门市), Húběi Province (湖北省), China [37];
- *Parajeilongvirus myotis* for Wǔfēng Myotis altarium paramyxovirus 1 (here decided to be abbreviated WfMaPV1) discovered by HTS in Szechwan myotis (vespertilionid *Myotis altarium* Thomas, 1911) sampled in Wǔfēng (五峰镇), Húběi Province (湖北省), China [37];
- *Parajeilongvirus pipistrelli* for piparella virus (PipaV) discovered by HTS in common pipistrelles [vespertilionid *Pipistrellus pipistrellus* (Schreber, 1774)] sampled in Aalst, East Flanders (Oost-Vlaanderen/Flandre-Orientale/Ostflandern), Belgium [39];
- *Parajeilongvirus plecoti* for plecomyxo virus (PlecoV) discovered by HTS in brown long-eared bats [vespertilionid *Plecotus auritus* (Linnaeus, 1758)] sampled in Oudenaarde, East Flanders (Oost-Vlaanderen/Flandre-Orientale/Ostflandern), Belgium [39];
- *Parajeilongvirus rhinolophi* for Wǔfēng Rhinolophus pearsonii paramyxovirus 1 (here decided to be abbreviated WfRpPV1) discovered by HTS in Pearson’s horseshoe bats (rhinolophid *Rhinolophus pearsonii* Horsfield, 1851) sampled in Wǔfēng (五峰镇), Húběi Province (湖北省), China [37];
- *Parajeilongvirus wenzhouense* for Wēnzhōu Myotis davidii paramyxovirus 1 (here decided to be abbreviated WzMdPV1) discovered by HTS in David’s myotis (vespertilionid *Myotis davidii* Peters, 1869) sampled in Wēnzhōu (温州市), Zhèjiāng Province (浙江省), China [37]; and
- *Parajeilongvirus zhejiangense* for Wēnzhōu Myotis laniger paramyxovirus 2 (here decided to be abbreviated WzMlPV2) discovered by HTS in Chinese water myotis (vespertilionid *Myotis laniger* Peters, 1871) sampled in Wēnzhōu (温州市), Zhèjiāng Province (浙江省), China [37].

Genus *Paramorbillivirus* was created for four new species:

- *Paramorbillivirus berylmysis* for Lóngquán Berylmys bowersi morbillivirus 1 (here decided to be abbreviated LqBbMV1) discovered by HTS in a Bower’s berylmys [murid *Berylmys bowersi* (Anderson, 1879)] sampled in Lóngquán (龙泉市), Zhèjiāng Province (浙江省), China [37];
- *Paramorbillivirus gierlense* for Gierle apodemus virus (GapV) discovered by HTS in a long-tailed field mouse [murid *Apodemus sylvaticus* (Linnaeus, 1758)] sampled in Gierle, Antwerp Province (Provincie Antwerpen/Province d'Anvers/Provinz Antwerpen), Belgium [38];
- *Paramorbillivirus niviventris* for Wǔfēng Niviventer fulvescens morbillivirus 1 (here decided to be abbreviated WfNfMV1) discovered by HTS in Indomalayan niviventer [murid *Niviventer fulvescens* (Gray, 1847)] sampled in Wǔfēng (五峰镇), Húběi Province (湖北省), China [37]; and
- *Paramorbillivirus pueyrredonense* for Ratón oliváceo morbillivirus (RoMV) discovered by screening public RNA-Seq data from hairy soft-haired akodonts [cricetid *Abrothrix hirta* (Thomas, 1895)] sampled around Pueyrredón/Cochrane Lake, Santa Cruz Province (Provincia de Santa Cruz), Argentina, and from olive-coloured akodonts [cricetid *Abrothrix olivacea* (Waterhouse, 1837)] sampled in Aysén Province (Provincia de Aysén), Chile [49].

Genus *Tupaivirus* was created for species *Narmovirus tupaiae* (moved from genus *Narmovirus* and renamed as *Tupaivirus tupaiae*).

##### Subfamily *Rubulavirinae*

Genus *Orthorubulavirus* was expanded by one new species, *Orthorubulavirus rhinolophi*, for Wǔfēng Rhinolophus sinicus rubulavirus 1 (here decided to be abbreviated WfRsRV1) discovered by HTS in Chinese rufous horseshoe bats (rhinolophid *Rhinolophus sinicus* K. Andersen, 1905) sampled in Wǔfēng (五峰镇), Húběi Province (湖北省), China [37]. Species *Orthorubulavirus alstonvillense* was abolished.

Genus *Pararubulavirus* was expanded by one new species, *Pararubulavirus eidoli*, for Achimota pararubulavirus 3 (AchPV3) isolated from straw-coloured fruit bats (pteropodid *Eidolon helvum* Kerr, 1792) sampled in Accra, Ghana [50].

### Family *Rhabdoviridae*

A new subfamily, *Deltarhabdovirinae*, was created to contain four new genera:

Genus *Gammahymrhavirus* was created for three species (TaxoProp 2023.021M.Rhabdoviridae_24nsp_5ngen_1nsf):

- *Gammahymrhavirus cerana* for Apis rhabdovirus 4 (ApRV4) discovered by HTS in Western honey bees (apid *Apis mellifera* Linnaeus, 1758) sampled in Hēilóngjiāng Province (黑龙江省), China (unpublished; GenBank #MZ822106);
- *Gammahymrhavirus longicaudata* for Diachasmimorpha longicaudata rhabdovirus (DlonRV) discovered by HTS in parasitoid wasps [braconid *Diachasmimorpha longicaudata* (Ashmead, 1905)] of tephritid fruit flies sampled in FL, USA (unpublished; GenBank #KP735609); and
- *Gammahymrhavirus mellifera* for Apis rhabdovirus 5 (ApRV5) discovered by HTS in Asian honey bees (apid *Apis cerana* Fabricius, 1793) sampled in Zhèjiāng Province (浙江省), China (unpublished; GenBank #MZ822105).

Genus *Primrhavirus* was created for three reassigned species (TaxoProp 2023.021M.Rhabdoviridae_24nsp_5ngen_1nsf):

- *Primrhavirus atrato* for Atrato rhabdo-like virus 3 (AtRLV3) discovered by HTS in mosquitoes (culicid *Culex* sp.) sampled in Colombia (unpublished; GenBank #MN661034);
- *Primrhavirus gabriel* for San Gabriel mononegavirus (SGMNV) discovered in an RNA-Seq library from a common Asian tiger mosquito [culicid *Aedes albopictus* (Skuse, 1894)] colony maintained in San Gabriel Valley, CA, USA [51]; and
- *Primrhavirus primus* for Primus virus (PRIMV) discovered by HTS in inland floodwater mosquitoes [culicid *Aedes vexans* (Meigen, 1830)] sampled in Younoufere, Matam Region, Senegal [52];

Genus *Stangrhavirus* was created for four new species (TaxoProp 2023.021M.Rhabdoviridae_24nsp_5ngen_1nsf):

- *Stangrhavirus elisy* for Elisy virus (ELSYV) discovered by HTS in western encephalitis mosquitoes (culicid *Culex tarsalis* Coquillett, 1896) sampled in Alameda County, CA, USA [53];
- *Stangrhavirus guadeloupe* for Guadeloupe Culex rhabdovirus (GCRV; sample 2017-PB-CQM-1-3) discovered by HTS in southern house mosquitoes (culicid *Culex quinquefasciatus* Say, 1823) sampled in Guadeloupe [54];
- *Stangrhavirus stang* for Stang virus (STNGV) discovered by HTS in tule mosquitoes (culicid *Culex erythrothorax* Dyar, 1907) sampled in the Placer Valley, CA [53]; and
- *Stangrhavirus wuhan* for Wuhan mosquito virus 9 (WhMV9) discovered by HTS in mosquitoes (culicid *Culex tritaeniorhynchus* Giles, 1901) sampled in Wǔhàn (武汉市), Húběi Province (湖北省), China [55].

Genus *Gammaricinrhavirus* was created for two species (TaxoProp 2023.021M.Rhabdoviridae_24nsp_5ngen_1nsf):

- *Gammaricinrhavirus fuyun* for Fuyun tick rhabdovirus (FyTRV) discovered by HTS in ticks (ixodid *Hyalomma* sp.) sampled in Fùyùn County (富蕴县), Xīnjiāng Uygur Autonomous Region (新疆维吾尔自治区), China [15] and
- *Gammaricinrhavirus tacheng* for Tacheng tick virus 7 (TcTV7) discovered by HTS in ticks (argasid *Argas miniatus* Koch, 1844) sampled in Tǎchéng, Xīnjiāng Uygur Autonomous Region (新疆维吾尔自治区), China [55].

The established genera *Alphacrustrhavirus*, *Alphadrosrhavirus*, *Alphahymrhavirus*, *Betahymrhavirus*, *Betanemrhavirus*, *Betapaprhavirus* and *Betaricinrhavirus*, previously unassigned to subfamilies, were moved into subfamily *Deltarhabdovirinae* (TaxoProp 2023.021M.Rhabdoviridae_24nsp_5ngen_1nsf).

Genus *Alphahymrhavirus* was expanded by two new species:

- *Alphahymrhavirus distinguendus* for Lariophagus distinguendus negative-strand RNA virus 1 (LdNSRV1) discovered by HTS in parasitoid wasps [pteromalid *Lariophagus distinguendus* (Förster, 1841)] of rice weevil larvae and pupae sampled in Hángzhōu (杭州市), Zhèjiāng Province (浙江省), China [56] and
- *Alphahymrhavirus xiangshan* for Xiangshan rhabdo-like virus 3 (XsRLV3) discovered by HTS in a mixed sample of dipteran, hymenopteran and lepidopteran insects sampled in Beijing, China (unpublished, GenBank #OK491501).

Genus *Betahymrhavirus* was expanded by one new species, *Betahymrhavirus xiangshan*, for Xiangshan rhabdo-like virus 4 (XsRLV4; sample Novel_26) discovered by HTS in a mixed sample of insects (Hymenoptera, Diptera and Lepidoptera) sampled in Beijing, China (unpublished; GenBank #OK491502).

Genus *Betaricinrhavirus* was expanded by three new species (TaxoProp 2023.021M.Rhabdoviridae_24nsp_5ngen_1nsf):

- *Betaricinrhavirus mudanjiang* for Mudanjiang rhabd tick virus 1 (MjRTV1) discovered by HTS in ticks [ixodid *Ixodes persulcatus* (Schulze, 1930)] collected from the field in Jílín Province (吉林省), China (unpublished; GenBank #ON746525);
- *Betaricinrhavirus tongren* for Tongren rhabd tick virus 1 (TrRTV1) discovered by HTS in East Asian mountain haemaphysalids (ixodid *Haemaphysalis hystricis* Supino, 1897) collected from swine in Guìzhōu Province (贵州省), China (unpublished; GenBank #ON746523); and
- *Betaricinrhavirus yanbian* for Yanbian rhabd tick virus 3 (YbRTV3) discovered by HTS in ticks (ixodid *Haemaphysalis japonica* Warburton, 1908) collected from the field in Jílín Province (吉林省), China (unpublished; GenBank #ON746524).

#### Subfamily *Alpharhabdovirinae*

Subfamily *Alpharhabdovirinae* was expanded by one new genus, *Uniorhavirus*, including one new species, *Uniorhavirus killamcar*, for killamcar virus 1 (KILLV1) discovered by HTS in a plain pocketbook (unionid *Lampsilis cardium* Rafinesque, 1820) sampled in Kilmore Creek, IN, USA [57] (2023.002 M.A.Alpharhabdovirinae_2ngen_2nsp).

In genus *Almendravirus*, species name *Almendravirus xianshan* was corrected to *Almendravirus xiangshan* (TaxoProp 2023.001M.A.Almendravirus_1sp_rename.docx). The genus was expanded by one new species, *Almendravirus shanxi*, for Shanxi arboretum virus (SxABTV) isolated from a pool of mosquitoes [culicid *Armigeres subalbatus* (Coquillett, 1898)] sampled in Shānxī Province (山西省), China, 2019 [58] (TaxoProp 2023.003M.A.Alpharhabdovirinae_8nsp).

Genus *Alpharicinrhavirus* was expanded by 12 new species (TaxoProp 2023.015M.A.Alpharhabdovirinae_17nsp.docx):

- *Alpharicinrhavirus bancrofti* for Haemaphysalis bancrofti rhabdovirus (HbanRV) discovered by HTS in wallaby ticks (ixodid *Haemaphysalis bancrofti* Nuttall & Warburton, 1915) sampled in Australia (unpublished; GenBank #OK128265);
- *Alpharicinrhavirus huanggang* for Huanggang rhabd tick virus 1 (HgRTV1) discovered by HTS in ticks [ixodid *Rhipicephalus microplus* (Canestrini, 1888)] collected from cattle in Húběi Province (湖北省), China (unpublished; GenBank #ON74651);
- *Alpharicinrhavirus huangpi* for Huangpi tick virus 3 (HpTV3) discovered by HTS in Doenitz’ Oriental-Australian bird haemaphysalids (ixodid *Haemaphysalis doenitzi* Warburton & Nuttall, 1909) sampled in Wǔhàn (武汉市), Húběi Province (湖北省), China [59];
- *Alpharicinrhavirus jilin* for Yanbian rhabd tick virus 1 (YbRTV1) discovered by HTS in taiga ticks [ixodid *Ixodes persulcatus* (Schulze, 1930)] sampled in Jílín Province (吉林省), China (unpublished; GenBank #ON746520);
- *Alpharicinrhavirus nanning* for Nanning rhabd tick virus 1 (NnRTV1) discovered by HTS in Asian monitor lizard ticks (ixodid *Amblyomma varanense* Supino, 1897) sampled in Guǎngxī Zhuàng Autonomous Region (广西壮族自治区), China (unpublished; GenBank #ON746519);
- *Alpharicinrhavirus ningxia* for Guyuan rhabd tick virus 1 (GyRTV1) discovered by HTS in ticks (ixodid *Haemaphysalis japonica* Warburton, 1908) collected from sheep in Níngxià Hui Autonomous Region (宁夏回族自治区), China (unpublished; GenBank #PP945241);
- *Alpharicinrhavirus qinghai* for Yushu rhabd tick virus 2 (YsRTV2) discovered by HTS in ticks (ixodid *Dermacentor everestianus* Hirst, 1926) collected from cattle in Qīnghǎi Province (青海省), China (unpublished; GenBank #ON746530);
- *Alpharicinrhavirus reticulatus* for Dermacentor reticulatus rhabdovirus 1 (DretRV1) discovered by HTS in ornate cow ticks (ixodid *Dermacentor reticulatus* Fabricius, 1794) sampled in Croatia [23];
- *Alpharicinrhavirus skanevik* for Norway mononegavirus 1 (NWMV1) discovered by HTS in ticks [ixodid *Ixodes ricinus* (Linnaeus, 1758)] sampled in the region of former Skånevik municipality, Norway [60];
- *Alpharicinrhavirus tahe* for Tahe rhabdovirus 1 (ThRV1) discovered by HTS in relict ticks (ixodid *Haemaphysalis concinna* C. L. Koch, 1844) sampled in Tǎhé County (塔河县), Hēilóngjiāng Province (黑龙江省), China [61]. Yanbian rhabd tick viruses 4 (YbRTV4) and 5 (YbRTV4) (unpublished; GenBank #ON746526 and #ON746537, respectively) are considered to be isolates of ThRV1, and their names and abbreviations should not be used anymore;
- *Alpharicinrhavirus taishun* for Taishun tick virus (TsTV) discovered by HTS in East Asian mountain haemaphysalids (ixodid *Haemaphysalis hystricis* Supino, 1897) sampled in Tàishùn County (泰顺县), Zhèjiāng Province (浙江省), China [59]; and
- *Alpharicinrhavirus zhangjiakou* for Zhangjiakou rhabd tick virus 1 (ZjRTV1) discovered by HTS in ticks (ixodid *Dermacentor silvarum* Olenev, 1931) sampled in Inner Mongolia Autonomous Region (内蒙古自治区), China (unpublished; GenBank #ON746531).

Genus *Amplylivirus* was expanded by one new species, *Amplylivirus pugnax*, for Boana pugnax lyssa-like virus 1 (BpugLLV1) discovered by HTS in Chirique-Flusse tree frogs [hylid *Boana pugnax* (Spix, 1824)] sampled in Colombia (unpublished; GenBank #MZ682616) (TaxoProp 2023.015M.Alpharhabdovirinae_17nsp.docx).

Genus *Ephemerovirus* was expanded by one new species, *Ephemerovirus huanggang*, for Huanggang rhabd tick virus 2 (HgRTV2) discovered by HTS in ticks [ixodid *Rhipicephalus microplus* (Canestrini, 1888)] collected from cattle in Húběi Province (湖北省), China (unpublished; GenBank #ON746527) (TaxoProp 2023.010M.A.Ephemerovirus_1nsp).

Genus *Ledantevirus* was expanded by one new species, *Ledantevirus tongren*, for Tongren rhabd tick virus 2 (TrRTV2) discovered by HTS in ticks (ixodid *Haemaphysalis* sp.) collected from domestic pigs in Guìzhōu Province (贵州省), China (unpublished; GenBank #ON746534) (TaxoProp 2023.003M.A.Alpharhabdovirinae_8nsp).

Genus *Lostrhavirus* was expanded by one new species, *Lostrhavirus alxa*, for Alxa tick rhabdovirus (ATRV) discovered by HTS in ticks (ixodid *Hyalomma* sp.) collected from herbivore livestock in Xīnjiāng Uygur Autonomous Region (新疆维吾尔自治区) and Inner Mongolia Autonomous Region (内蒙古自治区), China [15] (TaxoProp 2023.003M.A.Alpharhabdovirinae_8nsp).

Genus *Lyssavirus* was expanded by one new species, *Lyssavirus kotalahti*, for Kotalahti bat lyssavirus (KBLV) discovered by HTS in Brandt’s myotis [vespertilionid *Myotis brandtii* (Eversmann, 1845)] sampled near Kotalahti, Finland [62,63] (TaxoProp 2023.012M.A.Lyssavirus_1nsp).

Genus *Merhavirus* was expanded by three new species (TaxoProp 2023.015M.Alpharhabdovirinae_17nsp.docx):

- *Merhavirus formosus* for Formosus virus (FORMV) discovered by HTS in yellow fever mosquitoes [culicid *Aedes aegypt*i (Linnaeus in Hasselquist, 1762)] from a laboratory colony originating from Bundibugyo, Western Region, Uganda [51];
- *Merhavirus hattula* for Hattula rhabdovirus (HTTRV) discovered by HTS in mosquitoes [culicid *Ochlerotatus communis* (de Geer, 1776)] sampled in Ilomantsi, North Karelia (Pohjois-Karjala/Norra Karelen), Finland [64]; and
- *Merhavirus inari* for Inari rhabdovirus (INARV) discovered by HTS in mosquitoes [culicid *Ochlerotatus communis* (de Geer, 1776)] sampled in Lapland, Finland [64].

Genus *Ohlsrhavirus* was expanded by one new species, *Ohlsrhavirus adumi*, for Adumi virus (ADUMV) discovered by HTS in unspecified mosquitoes sampled in Uganda (unpublished; GenBank #OQ077989) (TaxoProp 2023.015M.A.Alpharhabdovirinae_17nsp.docx).

Genus *Sigmavirus* was expanded by two new species (TaxoProp 2023.003M.A.Alpharhabdovirinae_8nsp):

- *Sigmavirus jopcycgri* for jopcycgri virus 1 (JPCGV1) discovered by HTS in bat flies (hippoboscid *Cyclopodia greeffi* Karsch, 1884) sampled in Nigeria [65] and
- *Sigmavirus tryoni* for Bactrocera tryoni rhabdovirus 1 (BtryRV1) discovered by HTS in Queensland fruit flies [tephritid *Bactrocera tryoni* (Froggatt, 1897)] sampled in Australia [66].

Genus *Tupavirus* was expanded by three new species (TaxoProp 2023.003M.A.Alpharhabdovirinae_8nsp):

- *Tupavirus incomtus* for tupavirus SB8301 (TUPVSB) discovered by HTS in common dasmys [murid *Dasymys incomtus* (Sundevall, 1847)] sampled in Kenya (unpublished; GenBank #OL774829);
- *Tupavirus stheno* for bat tupavirus BS1 (BtTVBS1) discovered by HTS in lesser brown horseshoe bats (rhinolophid *Rhinolophus stheno* K. Andersen, 1905) sampled in Yúnnán Province (云南省), China (unpublished; GenBank #OQ709183); and
- *Tupavirus stoliczkanus* for bat tupavirus BS2 (BtTVBS2) discovered by HTS in Stoliczka’s Asian trident bats [hipposiderid *Aselliscus stoliczkanus* (Dobson, 1871)] sampled in Yúnnán Province (云南省), China [67].

#### Subfamily *Betarhabdovirinae*

Subfamily *Betarhabdovirinae* was expanded by three new genera (2023.004 M.A.Betarhabdovirinae_2ngen_9nsp_move1sp):

Genus *Alphagymnorhavirus* for eight novel species:

- *Alphagymnorhavirus abietis* for Abies virus 1 (AbiV1) discovered in transcriptome data of Nordmann fir [pinaceaen *Abies nordmanniana* (Steven) Spach] from CT, USA [68];
- *Alphagymnorhavirus amentotaxi* for Amentotaxus virus 1 (AmeV1) discovered in transcriptome data of catkin yew [taxaceaen *Amentotaxus argotaenia* (Hance) Pilg.] from Chengdu, China [68];
- *Alphagymnorhavirus cupressi* for Cupressus virus 1 (CupV1) discovered in transcriptome data of Cheng cypress (cupressaceaen *Cupressus chengiana* S.Y.Hu) from Sìchuān Province (四川省), China [68];
- *Alphagymnorhavirus piceae* for Picea virus 1 (PicV1) discovered in transcriptome data of Qinghai spruce (pinacaean *Picea crassifolia* Kom.) from Qīnghǎi Province (青海省), China [68];
- *Alphagymnorhavirus pinibanksiae* for Pinus banksiana virus 1 (PiBanV1) discovered in transcriptome data of jack pine (pinaceaen *Pinus banksiana* Lamb.) from Alberta, Canada [68];
- *Alphagymnorhavirus piniyunannensis* for Pinis yunnanensis virus 1 (PiYunV1) discovered in transcriptome data of Yunnan pine (pinaceaen *Pinus yunnanensis* Franch.) from Yúnnán Province (云南省), China [68];
- *Alphagymnorhavirus sciadopitysis* for Sciadopitys virus 1 (SciV1) discovered in transcriptome data of Japanese umbrella pine [sciadopityaceaen *Sciadopitys verticillata* (Thunb.) Siebold and Zucc.] [68]; and
- *Alphagymnorhavirus taxi* for Taxus virus 1 (TaxV1) discovered in transcriptome data of hybrid yew (taxaceaen *Taxus*×*media* Rehder) from Hángzhōu (杭州市), Zhèjiāng Province (浙江省), China [68].

Species *Varicosavirus pini* was moved into genus *Alphagymnorhavirus* and renamed *Alphagymnorhavirus piniflexilis*.

Genus *Betagymnorhavirus* was established to include one new species, *Betagymnorhavirus torreyae*, for Torreya virus 1 (TorV1) discovered in transcriptome data of Chinese nutmeg yew (taxaceaen *Torreya grandis* Fortune ex Lindl.) sampled in Chéngdū (成都市), Sìchuān Province (四川省), China [68].

Genus *Deltanucleorhabdovirus* was established to include two new species (TaxoProp 2023.017M.A.Betarhabdovirinae_1 ng_7nsp):

- *Deltanucleorhabdovirus fragariae* for strawberry virus 3 (StrV3) discovered by HTS in strawberry (rosaceaen *Fragaria*×*ananassa* Duchesne) sampled in the USA [69] and
- *Deltanucleorhabdovirus medicagonis* for Medicago sativa virus 1 (MSV1) discovered by HTS in alfalfa (fabaceaen *Medicago sativa* L.) sampled in China [70].

Genus *Alphanucleorhabdovirus* was expanded by two new species (TaxoProp 2023.017M.A.Betarhabdovirinae_1 ng_7nsp):

- *Alphanucleorhabdovirus lycopersici* for tomato alphanucleorhabdovirus 1 (TARV1) discovered by HTS in tomato (solanaceaen *Solanum lycopersicum* L.) sampled in Strunjan, Coastal-Karst Statistical Region (Obalno-kraška statistična regija), Slovenia [71] and
- *Alphanucleorhabdovirus xinjianensis* for Xinjiang alphanucleorhabdovirus (XARV) discovered by HTS in horse faeces sampled in Xīnjiāng Uygur Autonomous Region (新疆维吾尔自治区), China [72].

Genus *Betanucleorhabdovirus* was expanded by four new species (TaxoProp 2023.017M.A.Betarhabdovirinae_1 ng_7nsp):

- *Betanucleorhabdovirus alphalycopersici* for tomato betanucleorhabdovirus 1 (TBRV1) discovered by HTS in tomato (solanaceaen *Solanum lycopersicum* L.) sampled in Beričevo, Central Slovenia Statistical Region (Osrednjeslovenska statistična regija), Slovenia [71];
- *Betanucleorhabdovirus betalycopersici* for tomato betanucleorhabdovirus 2 (TBRV2) discovered by HTS in tomato (solanaceaen *Solanum lycopersicum* L.) sampled in Beričevo, Central Slovenia Statistical Region (Osrednjeslovenska statistična regija), Slovenia [71];
- *Betanucleorhabdovirus picridis* for Picris betanucleorhabdvirus 1 (PBRV1) was discovered by HTS in bristly oxtongue [asteraceaen *Helminthotheca echioides* (L.) Holub)] sampled in Slovenia [71]; and
- *Betanucleorhabdovirus taraxi* for Taraxacum betanucleorhabdvirus 1 (TarBRV1) discovered by HTS in dandelion [asteraceaen *Taraxacum officinale* (L.) Weber ex F.H.Wigg.] sampled in Slovenia [71].

Genus *Cytorhabdovirus* was expanded by 12 new species (TaxoProp 2023.008M.A.Cytorhabdovirus_12nsp.docx):

- *Cytorhabdovirus betafragariae* for strawberry virus 2 (StrV2) discovered by HTS in strawberry (rosaceaen *Fragaria*×*ananassa* Duchesne) sampled in the USA [73];
- *Cytorhabdovirus betarosae* for rose-associated cytorhabdovirus (RaCV) discovered by HTS in sweetbriar rose (rosaceaen *Rosa rubiginosa* L.) sampled in Chóngqìng (重庆), China [74];
- *Cytorhabdovirus daphnis* for daphne virus 1 (DV1) discovered by HTS in winter daphne (thymelaeaceaen *Daphne odora* Thunb.) sampled in South Korea [75];
- *Cytorhabdovirus glycinis* for soybean blotchy mosaic virus (SbBMV) discovered by HTS in soybean [fabaceaen *Glycine max* (L.) Merr.] sampled in South Africa [76];
- *Cytorhabdovirus hyptisis* for Hyptis latent virus (HpLV) discovered by HTS in bushmint [lamiaceaen *Hyptis pectinata* (L.) Poit.] sampled in Ecuador [77];
- *Cytorhabdovirus pastinacae* for Pastinaca cytorhabdovirus 1 (PaCRV1) discovered by HTS in wild parsnip (apiaceaen *Pastinaca sativa* L.) sampled in Slovenia [71];
- *Cytorhabdovirus pogostemi* for Patchouly chlorosis-associated cytorhabdovirus (PcaCV) discovered by HTS in patchouly [lamiaceaen *Pogostemon cablin* (Blanco) Benth.] sampled in Pará, Brazil [78];
- *Cytorhabdovirus ribes* for blackcurrant rhabdovirus 2 (BCRV2) discovered by HTS in black currant (grossulariaceaen *Ribes nigrum* L.) sampled in the Czech Republic [79];
- *Cytorhabdovirus rudbeckiae* for Rudbeckia virus 1 (RudV1) discovered by HTS in coneflower (asteraceaen *Rudbeckia* sp.) sampled in China [80];
- *Cytorhabdovirus sambuci* for Sambucus virus 1 (SaV1) discovered by HTS in elderberry (adoxaceaen *Sambucus nigra* L.) sampled in the Czech Republic [81];
- *Cytorhabdovirus taraxaci* for Taraxacum cytorhabdovirus 1 (TCRV1) discovered by HTS in dandelion [asteraceaen *Taraxacum officinale* (L.) Weber ex F.H.Wigg.] sampled in Slovenia [71]; and
- *Cytorhabdovirus tiliae* for Tilia cytorhabdovirus 1 (TiCRV1) discovered by HTS in small-leaved lime (malvaceaen *Tilia cordata* Mill.) sampled in Germany [82].

Genus *Dichorhavirus* was expanded by one new species, *Dichorhavirus australis*, for citrus bright spot virus (CiBSV) discovered by HTS in sweet orange [rutacean *Citrus*×*sinensis* (L.) Osbeck] sampled in South Region (Região Sul), Brazil [83] (TaxoProp 2023.009M.A.Dichorhavirus_1nsp.docx).

Genus *Gammanucleorhabdovirus* was expanded by one new species, *Gammanucleorhabdovirus cerealis*, for cereal chlorotic mottle virus (CCMoV) discovered by HTS in oats (poaceaen *Avena sativa* L.) sampled in Morocco [84] (TaxoProp 2023.017M.A.Betarhabdovirinae_1 ng_7nsp).

Genus *Varicosavirus* was expanded by 32 new plant-infecting species (TaxoProp 2023.022M.Varicosavirus_32nsp):

- *Varicosavirus aconiti* for Aconitum virus 1 (AcoV1) discovered in transcriptome data of Kusnezoff monkshood (ranunculaceaen *Aconitum kusnezoffii* Rchb.) tissues from Jílín Province (吉林省), China [68];
- *Varicosavirus aperae* for Apera virus 1 (ApeV1) discovered in transcriptome data of common windgrass [poaceaen *Apera spica-venti* (L.) P.Beauv.] tissues from Denmark [68];
- *Varicosavirus aponogeti* for Aponogeton virus 1 (ApoV1) discovered in transcriptome data of lace plant [aponogetonaceaen *Aponogeton madagascariensis* (Mirb.) H. Bruggen] tissues from Canada [68];
- *Varicosavirus arceuthobii* for Arceuthobium virus 8 (ArcV8) discovered in transcriptome data of dwarf mistletoe [santalaceaen *Arceuthobium sichuanense* (H. S. Kiu) Hawksw. and Wiens] tissues from Qīnghǎi Province (青海省), China [68];
- *Varicosavirus artemisiae* for Artemisia virus 1 (ArtV1) discovered in transcriptome data of common wormwood (asteracean *Artemisia absinthium* L.) tissues from the UK [68];
- *Varicosavirus asclepiadis* for Asclepias syriaca virus 3 (AscSyV3) discovered in transcriptome data of common milkweed (apocynaceaen *Asclepias syriaca* L.) tissues from IL, USA [68];
- *Varicosavirus betabrassicae* for Brassica virus 2 (BrV2) discovered in transcriptome data of shortpod mustard [brassicacean *Hirschfeldia incana* (L.) Lagr.-Foss.], Indian mustard [brassicacean *Brassica juncea* (L.) Czern. *var*. *rugosa*] and Chinese kale (*Brassica oleracea* L. *var*. *alboglabra*) tissues from the USA, India and China, respectively [68];
- *Varicosavirus caladeniae* for Caladenia virus 1 (CalV1) discovered in transcriptome data of crab-lipped spider orchid (orchidaeceaen *Caladenia plicata* Fitzg.) tissues from Western Australia [68];
- *Varicosavirus centaureae* for Centaurea virus 1 (CenV1) discovered in transcriptome data of cornflower (asteraceaen *Centaurea cyanus* L.) tissues from New South Wales, Australia [68];
- *Varicosavirus cucumis* for Cucumis virus 1 (CucV1) discovered in transcriptome data of melon (cucurbitaceaen *Cucumis melo* L.) tissues from China [68];
- *Varicosavirus didymochlaenae* for Didymochlaena virus 1 (DidV1) discovered in transcriptome data of tree maidenhair fern [didymochlaenaceaen *Didymochlaena truncatula* (Sw.) J.Sm.] tissues from Indonesia [68];
- *Varicosavirus erysimi* for Erysimum virus 1 (EryV1) discovered in transcriptome data of wallflower [brassicaceaen *Erysimum bastetanum* (Blanca and C.Morales) Lorite, Perfectti and J.M.Gómez] tissues from Spain [68];
- *Varicosavirus frullaniae* for Frullania virus 1 (FruV1) discovered in transcriptome data of the liverwort (frullaniaceaen *Frullania orientalis* Sande Lacoste, 1855) from Sìchuān Province (四川省), China [68];
- *Varicosavirus guizotiae* for Guizotia virus 1 (GuiV1) discovered in transcriptome data of noog [asteraceaen *Guizotia abyssinica* (L.f.) Cass.] tissues from Australia [68];
- *Varicosavirus holci* for Holcus virus 1 (HolV1) discovered in transcriptome data of common velvet grass (poaceaen *Holcus lanatus* L.) tissues from Northern Ireland, UK [68];
- *Varicosavirus leucanthemi* for Leucanthemum virus 1 (LeuV1) discovered in transcriptome data of oxeye daisy (asteracean *Leucanthemum vulgare* Lam.) tissues from New South Wales, Australia [68];
- *Varicosavirus luffae* for Luffa virus 1 (LufV1) discovered in transcriptome data of sponge gourd (cucurbitaceaen *Luffa aegyptiaca* Mill.) tissues from Australia [68];
- *Varicosavirus meliloti* for Melilotus virus 1 (MelV1) discovered in transcriptome data of honey clover (fabacean *Melilotus albus* Medik.) and yellow sweet clover [*Melilotus officinalis* (L.) Lam.] tissues from China and Canada, respectively [68];
- *Varicosavirus monocleae* for Monoclea gottschei varicosa-like virus (MgVV) discovered in transcriptome data of the liverwort (monocleacean *Monoclea gottschei* Lindb., 1886) (unpublished; GenBank #OX380363–4);
- *Varicosavirus penniseti* for Pennisetum virus 1 (PenV1) discovered in transcriptome data of purple grass [poacean *Pennisetum violaceum* (Lam.) Rich.] tissues from India [68];
- *Varicosavirus primulae* for Primula virus 1 (PriV1) discovered in transcriptome data of splendour primrose (primulacean *Primula oreodoxa* Franch.) tissues from Sìchuān Province (四川省), China [68];
- *Varicosavirus ranunculi* for Ranunculus virus 1 (RanV1) discovered in transcriptome data of goldilocks buttercup (ranunculaceaen *Ranunculus auricomus* L.) tissues from Slovakia [68];
- *Varicosavirus raphani* for Raphanus virus 1 (RapV1) discovered in transcriptome data of radish (brassicacean *Raphanus raphanistrum sativus* L.) tissues from China [68];
- *Varicosavirus ribes* for Ribes virus 1 (RibV1) discovered in transcriptome data of Siberian currant (grossulariaceaen *Ribes diacanthum* Pall. 1776) tissues from China [68];
- *Varicosavirus silenis* for Silene virus 1 (SilV1) discovered in transcriptome data of bladder campion [caryophyllaceaen *Silene vulgaris* (Moench) Garcke] tissues from Slovakia [68];
- *Varicosavirus streptoglossae* for Streptoglossa virus 1 (StrV1) discovered in transcriptome data of broadhead daisy [asteracean *Streptoglossa macrocephala* (F.Muell.) Dunlop] tissues from Western Australia [68];
- *Varicosavirus tanaceti* for Tanacetum virus 1 (TanV1) discovered in transcriptome data of common tansy (asteracean *Tanacetum vulgare* L.) tissues from Germany [68];
- *Varicosavirus thrysopteris* for tree fern varicosa-like virus (TfVV) discovered in transcriptome data of tree fern (thyrsopteridaceaen *Thyrsopteris elegans* Kunze) tissues (unpublished; GenBank #OX380482–3);
- *Varicosavirus treubiae* for Treubia virus 1 (TreV1) discovered in transcriptome data of the liverwort [treubiaceaen *Treubia lacunosa* (Colenso) Prosk.] from China [68];
- *Varicosavirus tritici* for Triticum virus 1 (TriV1) discovered in transcriptome data of wheat (poaceaen *Triticum aestivum* L.) tissues from OH, USA [68];
- *Varicosavirus vincetoxici* for Vincetoxicum virus 1 (VinV1) discovered in transcriptome data of variegated swallow-wort (apocynaceaen *Cynanchum versicolor* Bunge) tissues from China [68]; and
- *Varicosavirus zeae* for Zea virus 1 (ZeaV1) discovered in transcriptome data of corn (poaceaen *Zea mays* L.) tissues from Germany [68].

#### Subfamily *Gammarhabdovirinae*

Subfamily *Gammarhabdovirinae* was expanded by one new genus, *Margarhavirus*, for one new species, *Margarhavirus chemarfal*, for chemarfal virus 1 (CHMFV1) discovered by HTS in a western pearlshell (margaritiferid *Margaritifera falcata* Gould, 1850) sampled in the Chehalis River, WA, USA [57] (2023.002 M.A.Alpharhabdovirinae_2ngen_2nsp).

#### Unassigned to a subfamily

A genus unassigned to a subfamily, *Platrhavirus*, was created for six new species (TaxoProp 2023.021M.Rhabdoviridae_24nsp_5ngen_1nsf):

- *Platrhavirus microphallus* for microrhabdovirus 1 (MicRV1) discovered in an RNA-Seq library from a fluke (plagiorchiid *Microphallus* sp.) [22];
- *Platrhavirus nodulosis* for triaenorhabdovirus 1 (TriRV1) discovered in an RNA-Seq library from a pike tapeworm [triaenophorid *Triaenophorus nodulosus* (Pallas, 1781) Rudolphi, 1793] [22];
- *Platrhavirus orientalis* for metorhabdovirus 2 (MetRV2) discovered in an RNA-Seq library from a liver fluke (plagiorchiid *Metorchis orientalis* Tanabe, 1920) [22];
- *Platrhavirus pseudoglobulus* for sphaeridiorhabdovirus 1 (SphRV1) discovered in an RNA-Seq library from a waterfowl fluke [psilostomatid *Sphaeridiotrema pseudoglobulus* (Rudolphi, 1814)] [22];
- *Platrhavirus turkestanicum* for schistorhabdovirus 1 (SchRV1) discovered in an RNA-Seq library from a blood fluke (schistosomatid *Schistosoma turkestanicum* Skrjabin, 191) [22]; and
- *Platrhavirus vulpes* for fox faecal rhabdovirus (FFRV) discovered by HTS in a red fox [canid *Vulpes vulpes* (Linnaeus, 1758)] sampled in Spain [85].

### Family *Xinmoviridae*

Family *Xinmoviridae* was expanded to include 10 new genera and 11 new species (TaxoProp 2023.023M.Xinmoviridae_10 ng_11nsp):

Genus *Corulvirus* for one new species, *Corulvirus hangzhouense*, for Hángzhōu Zicrona caerulea xinmovirus 1 (HaZCV1) discovered by HTS in blue shieldbugs (pentatomid *Zicrona caerulea* Linnaeus, 1758) sampled in Hángzhōu (杭州市), Zhèjiāng Province (浙江省), China [unpublished; GenBank #MZ209710);

Genus *Culivirus* for two new species:

- *Culivirus belgradiense* for Serbia mononega-like virus 1 (SerMV1) discovered by HTS in common house mosquitoes (culicid *Culex pipiens* Linnaeus, 1758) sampled in Belgrade, Serbia [86], and
- *Culivirus dunyae* for Aedes albopictus anphevirus (AealbAV) discovered by HTS in common Asian tiger mosquitoes [culicid *Aedes albopictus* (Skuse, 1894)[ sampled in the USA, Japan, Italy, China, Thailand and Switzerland [87];

Genus *Gordisvirus* for one new species, *Gordisvirus californiense*, for Gordis virus (GorV) discovered by HTS in mosquitoes [culicid *Culiseta particeps* (Adams, 1903)] sampled in San Diego County, CA, USA [53];

Genus *Gudgevirus* for one new species, *Gudgevirus namadgii*, for Gudgenby Calliphora mononega-like virus (GuCalMV) discovered by HTS in bluebodied blowflies [calliphorid *Calliphora augur* (Fabricius, 1775)] sampled in Gudgenby Valley, Namadgi National Park, Australian Capital Territory, Australia [88];

Genus *Laumovirus* for one new species, *Laumovirus liaoningense*, for Fǔshùn Monolepta lauta xinmovirus 2 (FuMLV2) discovered by HTS in leaf beetles (chrysomelid *Monolepta lauta* Gressitt & Kimoto, 1963) sampled in Fǔshùn (抚顺市), Liáoníng Province (辽宁省), China (unpublished; GenBank #MZ210031);

Genus *Molauvirus* for one new species, *Molauvirus liaoningense*, for Fǔshùn Monolepta lauta xinmovirus 1 (FuMLV1) discovered by HTS in leaf beetles (chrysomelid *Monolepta lauta* Gressitt & Kimoto, 1963) sampled in Fǔshùn (抚顺市), Liáoníng Province (辽宁省), China (unpublished; GenBank #MZ210030);

Genus *Nurnegvirus* for one new species, *Nurnegvirus hainanense*, for Sānyà Ischnura senegalensis xinmovirus 1 (SISeV1) discovered by HTS in common bluetails [coenagrionid *Ischnura senegalensis* (Rambur, 1842)] sampled in Sānyà (三亚市), Hǎinán Province (海南省), China (unpublished; GenBank #MZ209914);

Genus *Puclevirus* for one new species, *Puclevirus hangzhouense*, for Hángzhōu Cletus punctiger xinmovirus 1 (HaCPV1) discovered by HTS in rice stinkbugs [coreid *Cletus punctiger* (Dallas, 1852)] sampled in Hángzhōu (杭州市), Zhèjiāng Province (浙江省), China (unpublished; GenBank #MZ209673);

Genus *Tecephavirus* for one new species, *Tecephavirus sydneyense*, for bat faecal-associated anphe-like virus 1 (BtFAV1) discovered by HTS in the gut metagenome of grey-headed flying foxes (pteropodid *Pteropus poliocephalus* Temminck, 1825) sampled in Centennial Park, Sydney, Australia [89]; and

Genus *Trocevirus* for one new species, *Trocevirus haikouense*, for Bactrocera dorsalis xinmovirus 2 (BDorV) discovered by HTS in oriental fruit flies [tephritid *Bactrocera dorsalis* (Hendel, 1912)] in Hǎikǒu (海口市), Hǎinán Province (海南省), China (unpublished; GenBank #HG994135).

## Taxonomic changes in subphylum *Polyploviricotina*

### Class *Ellioviricetes*

Class *Ellioviricetes* was renamed *Bunyaviricetes*. One new order, *Hareavirales*, was established in the class, and families *Arenaviridae*, *Discoviridae*, *Leishbuviridae*, *Mypoviridae*, *Nairoviridae*, *Phenuiviridae* and *Wupedeviridae* were moved into this order from order *Bunyavirales* (TaxoProp 2023.024M.Bunyaviricetes) [90]. Family *Konkoviridae* was established in order *Hareavirales* (2023.006M.A.Bunyavirales_1nfam_1ngen_1nsp) [90] to include one new genus, *Olpivirus*, including one new species, *Olpivirus tulipae*, for tulip streak virus (TuSV) discovered by metagenomics in tulips (liliaceaen *Tulipa gesneriana* L.) [91].

#### Family *Arenaviridae*

Genus *Mammarenavirus* was expanded by nine species:

- *Mammarenavirus abaense* for Ābà-Miányáng virus (AMYV) discovered by HTS in plateau pikas [ochotonid *Ochotona curzoniae* (Hodgson, 1858)] sampled in Ngawa Tibetan and Qiang Autonomous Prefecture [阿坝藏族羌族自治州; abbreviated as Ābà (阿坝州)]/Miányáng (绵阳市), China [41] (TaxoProp 2023.027M.Mammarenavirus_6nsp);
- *Mammarenavirus batangense* for Bātáng virus [BTTV; formerly plateau pika virus (PPV)] discovered by HTS in plateau pikas [ochotonid *Ochotona curzoniae* (Hodgson, 1858)] sampled in Yùshù (玉树市), Qīnghǎi Province (青海省), China [92] (TaxoProp 2023.027M.Mammarenavirus_6nsp);
- *Mammarenavirus beregaense*, for Berega virus (BEGV) discovered by reverse transcription polymerase chain reaction (RT-PCR) screening in a woodland grammomys [murid *Grammomys surdaster* (Thomas and Wroughton, 1908; sensu Musser & Carleton 2005 and Bryja *et al*. 2017)] in Berega, Morogoro Region (Mkoa wa Morogoro), Tanzania [93] (TaxoProp 2023.014M.A.Mammarenavirus_2nsp);
- *Mammarenavirus ganziense* for Gānzī virus (GNZV) discovered by HTS in plateau pikas [ochotonid *Ochotona curzoniae* (Hodgson, 1858)] sampled in Garzê Tibetan Autonomous Prefecture [甘孜藏族自治州; abbreviated as Gānzī Prefecture (甘孜藏族自治州)], China [41] (TaxoProp 2023.027M.Mammarenavirus_6nsp);
- *Mammarenavirus mafigaense* for Mafiga virus (MAFV) discovered by RT-PCR screening in a single-striped lemniscomys [murid *Lemniscomys rosalia* (Thomas, 1904)] in Mafiga, Morogoro Region (Mkoa wa Morogoro), Tanzania [94,95] (TaxoProp 2023.013M.A.Mammarenavirus_1nsp);
- *Mammarenavirus mecsekense* for Mecsek Mountains virus (MEMV) discovered by RT-PCR in northern white-breasted hedgehogs (erinaceid *Erinaceus roumanicus* Barrett-Hamilton, 1900) sampled in Baranya County (Baranya vármegye), Hungary [96] (TaxoProp 2023.027M.Mammarenavirus_6nsp);
- *Mammarenavirus ngerengerense* for Ngerengere virus (NGEV) discovered by RT-PCR screening of southern African pygmy mice [murid *Mus* (*Nannomys*) *minutoides* (A Smith, 1834)] sampled in Morogoro Region (Mkoa wa Morogoro), Tanzania [93,95] (TaxoProp 2023.027M.Mammarenavirus_6nsp);
- *Mammarenavirus songeaense* for Songea virus (SOGV) discovered by RT-PCR screening in a woodland grammomys [murid *Grammomys surdaster* (Thomas and Wroughton, 1908; sensu Musser & Carleton 2005 and Bryja *et al*. 2017)] in Lilondo, Ruvuma Region (Mkoa wa Ruvuma), Tanzania [93] (TaxoProp 2023.014M.A.Mammarenavirus_2nsp); and
- *Mammarenavirus tietense* for Tietê virus (TIEV), discovered by RT-PCR in Seba’s short-tailed bats [phyllostomid *Carollia perspicillata* (Linnaeus, 1758)] sampled in southeastern Brazil [97] (TaxoProp 2023.027M.Mammarenavirus_6nsp).

#### Family *Nairoviridae*

Family *Nairoviridae* was expanded by one new genus, *Sitinavirus*, to include new species *Sitinavirus sichuanense* for Sìchuān tick nairovirus (ScTNV) discovered by HTS in unspecified ixodid ticks feeding on giant pandas in Sìchuān Province (四川省), China [98] (TaxoProp 2023.028M.Nairoviridae_1 ng_9nsp).

Genus *Orthonairovirus* was expanded by eight new species (TaxoProp 2023.028M.Nairoviridae_1 ng_9nsp):

- *Orthonairovirus antuense* for Āntú virus (ANTV) discovered by HTS in ticks (ixodid *Dermacentor silvarum* Olenev, 1931) sampled in Āntú County (安图县), Yánbiān Korean Autonomous Prefecture (延边朝鲜族自治州), Jílín Province (吉林省), China [99];
- *Orthonairovirus crocidurae* for Wǔfēng Crocidura attenuata orthonairovirus 1 (WfCAV1) discovered by HTS in Asian grey shrews (soricid *Crocidura attenuata* Milne-Edwards, 1872) sampled in Wǔfēng (五峰镇), Húběi Province (湖北省), China (unpublished; GenBank #OM030321–3);
- *Orthonairovirus gubboense* for Gubbo nairovirus (GUBV) discovered by HTS in ticks (argasid *Carios vespertilionis* Latreille, 1796) in Snesslinge, Uppsala County (Uppsala län), Sweden [100];
- *Orthonairovirus lambarenense* for lamusara virus (LMSV), discovered by RT-PCR in goliath shrews (soricid *Crocidura goliath* Thomas, 1906) sampled around Lambaréné, Moyen-Ogooué, Gabon [101];
- *Orthonairovirus manidae* for pangolin orthonairovirus (PONV) discovered by HTS in unspecified pangolins sampled in China (unpublished; GenBank #ON024087–9);
- *Orthonairovirus meihuashanense* for Méihuā Mountain virus (MHMV) discovered by HTS in ticks [ixodid *Haemaphysalis hystricis* Supino, 1897, *Haemaphysalis formosensis* Neumann, 1913, *Dermacentor taiwanensis* Sugimoto, 1935, and *Rhipicephalus microplus* (Canestrini, 1888)] sampled in the Méihuā Mountains (梅花山), Fújiàn Province (福建省), China [102];
- *Orthonairovirus sinense* for ‘orthonairovirus sp. isolate YS’ discovered by HTS in faeces of an unspecified shrew sampled in China (unpublished; GenBank #MW391927–9) – here renamed wawsin virus (WAWV);
- *Orthonairovirus sunci* for cencurut virus (CENV) discovered by HTS in Asian house shrews [soricid *Suncus murinus* (Linnaeus, 1766)] and in a nymphal tick (ixodid *Amblyomma helvolum* Koch, 1844) sampled in Singapore [103].

#### Family *Phenuiviridae*

The family was expanded by one new genus, *Bocivirus*, including three new species:

- *Bocivirus botrytidis* for Botrytis cinerea bocivirus 1 (BcBV1) discovered by HTS in grey mould [sclerotiniaceaen *Botrytis cinerea* Pers. (1794)] sampled in Spain [104];
- *Bocivirus fusarii* for Fusarium sibiricum coguvirus 1 (FsCV1) discovered by HTS in ascomycete fungi (nectriaceaen *Fusarium sibiricum* Gagkaeva, Burkin, Kononenko, Gavrilova, O'Donnell, T. Aoki, & Yli-Mattila, 2016) sampled in China [105]; and
- *Bocivirus trichodermae* for Trichoderma gamsii cogu-like virus 1 (TgClV1) discovered by HTS in ascomycete fungi [hypocreaceaen *Trichoderma gamsii* Samuels & Druzhinina (2006)] sampled in Italy [17].

Established species *Coguvirus viticulum* was renamed *Bocivirus viticulum* and moved into genus *Bocivirus* (TaxoProps 2023.019M.A.Phenuiviridae_1 ng_1nsp_1spmr and 2023.024 M.A.Bunyaviricetes).

Genus *Coguvirus* was expanded by one new species, *Coguvirus chrysanthae*, for Edgeworthia chrysantha mosaic-associated virus (ECMaV) discovered by HTS in Oriental paperbush (thymelaeaceaen *Edgeworthia chrysantha* Lindl.) sampled in China [106] (TaxoProp 2023.007M.A.Coguvirus_1nsp).

Genus *Mechlorovirus* was expanded by one new species, *Mechlorovirus ramuense*, for Ramu stunt virus (RmSV) discovered by HTS in sugarcane (panicoideaen *Saccharum officinarum* L.) sampled in Madang Province (Madang Provins), Papua New Guinea [107] (TaxoProp 2023.020M.Phenuiviridae_4nsp).

In genus *Phlebovirus*, species *Frijoles phlebovirus* was renamed *Phlebovirus limboense* (TaxoProp 2023.032M.Phlebovirus_1nsp_rename).

Genus *Tenuivirus* was expanded by three new species (TaxoProp 2023.020M.Phenuiviridae_4nsp):

- *Tenuivirus festucae* for Festuca stripe-associated virus (FSaV) discovered by HTS in meadow fescue (poaceaen *Festuca pratensis* Huds.) sampled in Saxony-Anhalt (Sachsen-Anhalt), Germany [108];
- *Tenuivirus kwazuluense* for wheat yellows virus (WhYV) discovered by HTS in common wheat (poaceaen *Triticum aestivum* L.) sampled in KwaZulu-Natal, South Africa [109]; and
- *Tenuivirus pontaense* for wheat white spike virus (WWSV) discovered by HTS in common wheat (poaceaen *Triticum aestivum* L.) sampled in Ponta Grossa, Paraná State, Brazil [110].

### Order *Bunyavirales*

Order *Bunyavirales* was renamed *Elliovirales* (TaxoProp 2023.024M.Bunyaviricetes) [90].

#### Family *Fimoviridae*

Genus *Emaravirus* was expanded by three new species:

- *Emaravirus ailanthi* for Ailanthus crinkle leaf-associated virus (ACrLaV) discovered by RNA-Seq and RT-PCR in Chinese sumac [simaroubaceaen *Ailanthus altissima* (Mill.) Swingle] sampled in Gàizhōu (盖州市), Liáoníng Province (辽宁省), China [111] (TaxoProp 2023.011P.A.Emaravirus_1nsp);
- *Emaravirus artemisiae* for Artemisia fimovirus 1 (ArtV1) discovered by HTS in Chinese mugwort (asteraceaen *Artemisia verlotiorum* Lamotte) sampled in Slovenia [71] (TaxoProp 2023.010P.A.Emaravirus_1nsp); and
- *Emaravirus kudzu* for Pueraria lobata-associated emaravirus (PloaEV) discovered by HTS in kudzu vine [fabaceaen *Pueraria montana var*. lobata (Willd.) Maesen and S.M.Almeida ex Sanjappa and Predeep] sampled in Běibèi (北碚), Chóngqìng (重庆), China [112] (TaxoProp 2023.009P.A.Emaravirus_1ns).

### Family *Hantaviridae*

Genus *Percilovirus* was created to include one new species, *Percilovirus gobii*, for Luposicya lupus actinovirus (LlAV) discovered by HTS in wolfsnout goby (gobiid *Luposicya lupus* J. L. B. Smith, 1959) sampled in Orpheus Island, North Queensland, Australia [113]. Species *Actinovirus lophii* was renamed *Percilovirus lophii* and moved from genus *Actinovirus* into genus *Percilovirus* (TaxoProp 2023.035M.Hantaviridae_reorg).

Genus *Mobatvirus* was expanded by one new species, *Mobatvirus dakrongense*, for Đakrông virus discovered by HTS in Stoliczka’s Asian trident bats [hipposiderid *Aselliscus stoliczkanus* (Dobson, 1871)] sampled in Đakrông Nature Reserve, Quảng Trị Province, Vietnam [114]. *Orthohantavirus robinaense* was renamed *Mobatvirus robinaense* and moved from genus *Orthohantavirus* into genus *Mobatvirus* (TaxoProp 2023.035M.Hantaviridae_reorg).

Genus *Orthohantavirus* was expanded to include three species (TaxoProp 2023.035M.Hantaviridae_reorg):

- *Orthohantavirus artybashense* for Artybash virus (ARTV) discovered by HTS in Laxmann’s shrews (soricid *Sorex caecutiens* Laxmann, 1788) sampled near Lake Teletskoye/Teleckoe (Телецкое озеро), Altai/Altaj Republic (Алтай Республика), Russia [115];
- *Orthohantavirus lankaense* for Lanka virus (LNKV) discovered by HTS in little Indian field mice [murid *Mus booduga* (Gray, 1837)] sampled in Polonnaruwa District (පොළොන්නරුව දිස්ත්‍රික්කය), Sri Lanka [116]; and
- *Orthohantavirus wufengense* for Wǔfēng Chodsigoa smithii orthohantavirus 1 (WfCsOHV1) discovered by HTS in Smith’s shrews [soricid *Chodsigoa smithii* (Thomas, 1911)] sampled in Wǔfēng (五峰镇), Húběi Province (湖北省), China (unpublished; GenBank #OM030311–3).

Species *Orthohantavirus moroense* and *Orthohantavirus negraense* were renamed *Orthohantavirus carrizalense* and *Orthohantavirus mamorense*, respectively. Species *Orthohantavirus fusongense*, *Orthohantavirus necocliense*, *Orthohantavirus oxbowense*, *Orthohantavirus seewisense* and *Orthohantavirus yakeshiense* were abolished. Adler virus (ADLV), Castelo dos Sonhos virus (CASV), El Moro Canyon virus (ELMCV), gōu virus, Laguna Negra virus (LANV), Lechiguanas virus (LECV; LECHV), Liánghé virus (LHEV), Orán virus (ORNV) and Serang virus (SERV) were removed from established species and now count as unclassified (TaxoProp 2023.035M.Hantaviridae_reorg).

#### Family *Peribunyaviridae*

Genus *Gryffinivirus* was created for two new species (TaxoProp 2023.031M.Peribunyaviridae_1 ng_7nsp):

- *Gryffinivirus alamedaense* for Udune virus (UDNV) discovered by HTS in a single mosquito (culicid *Culex pipiens*, Linnaeus, 1758] sampled in CA, USA [53] and
- *Gryffinivirus hedwigae* for Hedwig virus (HEDV) discovered by HTS in captive snowy owls [strigid *Bubo scandiacus* (Linnaeus, 1758)] sampled in Germany [117].

Genus *Orthobunyavirus* was expanded by five new species (TaxoProp 2023.031M.Peribunyaviridae_1 ng_7nsp):

- *Orthobunyavirus barritaense* for Barrita virus (BITV) isolated from mosquitoes [culicid *Psorophora varipes* (Coquillett, 1904)] sampled near Isla Morelos, Chiapas, Mexico [118];
- *Orthobunyavirus belmontense* for Belmont virus (BELV) isolated from common banded mosquitoes (culicid *Culex annulirostris* Skuse, 1889) sampled in Belmont, Australia [119];
- *Orthobunyavirus parkeri* for Parker’s Farm virus (PFV) isolated from common banded mosquitoes (culicid *Culex annulirostris* Skuse, 1889) sampled in Queensland, Australia [120];
- *Orthobunyavirus sussexense* for Little Sussex virus (LTLSV) isolated from common banded mosquitoes (culicid *Culex annulirostris* Skuse, 1889) sampled near Sussex Inlet, Australia [120]; and
- *Orthobunyavirus yacaabaense* for Yacaaba virus (YACV) isolated from mosquitoes [culicid *Ochlerotatus vigilax* (Skuse, 1889)] sampled near Mount Yacaaba, New South Wales, Australia [121].

Pahayokee virus (PAHV) was identified as an isolate of Shark River virus (SRV; species *Orthobunyavirus squalofluvii*). Thus, the name Pahayokee virus (PAHV) should not be used anymore (TaxoProp 2023.031M.Peribunyaviridae_1 ng_7nsp).

In genus *Pacuvirus*, Santarém virus (STMV) was identified as an isolate of Tapirapé virus (TAPV; species *Pacuvirus tapirapeense*). Thus, the name Santarém virus (STMV) should not be used anymore (TaxoProp 2023.031M.Peribunyaviridae_1 ng_7nsp).

#### Family *Phasmaviridae*

In genus *Orthophasmavirus*, an error was corrected by switching the assignment of two viruses: *Orthophasmavirus fushunense* now harbours Fǔshùn phasmavirus 2 (FsnPV2) instead of Fǔshùn phasmavirus 1 (FsnPV1) and *Orthophasmavirus sogatellae* now harbours FsnPV1 instead of FsnPV2 (TaxoProp 2023.030M.A.Orthophasmavirus_2sp_name_switch).

## Conclusions

A summary of the current ICTV-accepted taxonomy of the phylum *Negarnaviricota* is presented in Tables1^9^ and in the form of two posters (Fig. S1, available in the online Supplementary Material).

**Table 1.** Taxonomy of the order *Muvirales* (*Negarnaviricota: Haploviricotina: Chunqiuviricetes*) as of April 2024

Genus	Species*	Virus (abbreviation)†
**Family *Qinviridae*** [122]		
*Yingvirus*	*Yingvirus beihaiense*	Běihǎi sesarmid crab virus 4 (BhSCV4)
	*Yingvirus charybdis*	Wēnzhōu qinvirus-like virus 2 (WzQLV2)
	*Yingvirus hubeiense*	Húběi qinvirus-like virus 1 (HbQLV1)
	*Yingvirus sanxiaense*	Sānxiá qinvirus-like virus 1 (SxQLV1)
	*Yingvirus shaheense*	Shāhé qinvirus-like virus 1 (ShQLV1)
	*Yingvirus wenzhouense*	Wēnzhōu qinvirus-like virus 1 (WzQLV1)
	*Yingvirus wuhanense*	Wǔhàn insect virus 15 (WhIV15)
	*Yingvirus xinzhouense*	Xīnzhōu nematode virus 3 (XzNV3)

Note that viruses are real objects that are assigned to concepts that are called taxa. Species, genera, families and orders are taxa.

* Taxon names are always italicized and always begin with a capital letter.

† Virus names are not italicized and are not capitalized, except if the name or a name component is a proper noun. This column lists the virus names with their correct (lack of) capitalization.

**Table 2.** Taxonomy of the order *Naedrevirales* (*Negarnaviricota: Haploviricotina: Milneviricetes*) as of April 2024

Genus	Species*	Virus (abbreviation)†
**Family *Aspiviridae*** [123,124]		
*Ophiovirus*	*Ophiovirus capsici*	pepper chlorosis associated virus (PepCaV)
	*Ophiovirus citri*	citrus psorosis virus (CPsV)
	*Ophiovirus freesiae*	freesia sneak virus (FreSV)
	*Ophiovirus lactucae*	lettuce ring necrosis virus (LRNV)
	*Ophiovirus mirafioriense*	Mirafiori lettuce big-vein virus (MLBVV)
	*Ophiovirus ranunculi*	ranunculus white mottle virus (RWMV)
	*Ophiovirus tulipae*	tulip mild mottle mosaic virus (TMMMV)
	*Ophiovirus vaccinii*	blueberry mosaic-associated virus (BlMaV)

Note that viruses are real objects that are assigned to concepts that are called taxa. Species, genera, families and orders are taxa.

* Taxon names are always italicized and always begin with a capital letter.

† Virus names are not italicized and are not capitalized, except if the name or a name component is a proper noun. This column lists the virus names with their correct (lack of) capitalization.

**Table 3.** Taxonomy of the order *Jingchuvirales *[125] (*Negarnaviricota: Haploviricotina: Monjiviricetes*) as of April 2024

Genus	Species*	Virus (abbreviation)†
**Family** ***Aliusviridae***		
*Obscuruvirus*	*Obscuruvirus quintum*	Atrato chu-like virus 5 (AClV5)
*Ollusvirus*	*Ollusvirus coleopteri*	Húběi coleoptera virus 3 (HbCV3)
	*Ollusvirus culvertonense*	Culverton virus (CvV)
	*Ollusvirus hanchengense*	Hánchéng leafhopper mivirus (HLMV)
	*Ollusvirus hymenopteri*	hymenopteran chu-related virus 123 (HCrV123)
	*Ollusvirus insectii*	hymenopteran chu-related virus 126 (HCrV126)
	*Ollusvirus nomadae*	Nomada lathburiana mononega-like virus (NLmlV)
	*Ollusvirus oropsyllae*	Oropsylla silantiewi mononega-like virus 2 (OSmlV2)
	*Ollusvirus rhagoveliae*	Rhagovelia obesa mononega-like virus (ROmlV)
	*Ollusvirus scaldisense*	Scaldis River bee virus (SRBV)
	*Ollusvirus shayangense*	Shāyáng fly virus 1 (SyFV1)
	*Ollusvirus taiyuanense*	Tàiyuán leafhopper virus (TYLeV)
**Family *Chuviridae***		
*Boscovirus*	*Boscovirus hippoboscidae*	Wǔhàn louse fly virus 7 (WhLFV7)
	*Boscovirus hypoboscidae*	Wǔhàn louse fly virus 6 (WhLFV6)
*Chuvivirus*	*Chuvivirus brunnichi*	Wēnlǐng crustacean virus 14 (WlCV14)
	*Chuvivirus canceris*	Wēnzhōu crab virus 2 (WzCV2)
*Culicidavirus*	*Culicidavirus culicidae*	Wǔhàn mosquito virus 8 (WhMV8)
	*Culicidavirus culicis*	Culex mosquito virus 5 (ClMV5)
	*Culicidavirus imjinense*	Imjin River virus 1 (IjRV1)
	*Culicidavirus quitotaense*	Culex mosquito virus 4 (ClMV4)
*Demapteravirus*	*Demapteravirus dermapteri*	dermapteran chu-related virus 142 (DCrV142)
*Doliuvirus*	*Doliuvirus culisetae*	Mos8Chu0 chuvirus (MoCV)
*Mivirus*	*Mivirus amblyommae*	lone star tick chuvirus 1 (LSTCV1)
	*Mivirus belostomatis*	Belostoma flumineum mononega-like virus (BFmlV)
	*Mivirus boleense*	Bólè tick virus 3 (BTV3)
	*Mivirus changpingense*	Chāngpíng tick virus 2 (CpTV2)
	*Mivirus dermacentoris*	Chāngpíng tick virus 3 (CpTV3)
		Tǎchéng tick virus 5 (TcTV5)
	*Mivirus genovaense*	Genoa virus (GeV)
	*Mivirus karukeraense*	Karukera tick virus (KtV)
	*Mivirus rhipicephali*	brown dog tick mivirus 1 (BDTMV1)
	*Mivirus suffolkense*	Suffolk virus (SFKV)
	*Mivirus wuhanense*	Wǔhàn tick virus 2 (WhTV2)
*Morsusvirus*	*Morsusvirus argatis*	Tǎchéng tick virus 4 (TcTV4)
*Nigecruvirus*	*Nigecruvirus ixodes*	blacklegged tick chuvirus 2 (BlTCV2)
	*Nigecruvirus periplanetae*	Periplaneta americana mononega-like virus (PAmlV)
*Odonatavirus*	*Odonatavirus draconis*	odonatan chu-related virus 137 (OCrV137)
	*Odonatavirus fabricii*	Húběi odonate virus 11 (HbOV11)
	*Odonatavirus odontis*	odonatan chu-related virus 136 (OCrV136)
*Pediavirus*	*Pediavirus cirripedis*	Běihǎi barnacle virus 9 (BhBV9)
*Piscichuvirus*	*Piscichuvirus craterocephali*	hardyhead chuvirus (HhCV)
	*Piscichuvirus franki*	Herr Frank virus 1 (HFrV1)
	*Piscichuvirus lycodontis*	Guǎngdōng red-banded snake chuvirus-like virus (GRSCV)
	*Piscichuvirus sanxiaense*	Sānxiá atyid shrimp virus 4 (SxASC4)
	*Piscichuvirus wenlingense*	Wēnlǐng fish chu-like virus (WFClV)
*Pterovirus*	*Pterovirus chulinense*	hymenopteran chu-related virus OKIAV147 (HCrV147)
*Rochuvirus*	*Rochuvirus chalcocoris*	Chalcocoris rutilans mononega-like virus 2 (CRmlV2)
*Scarabeuvirus*	*Scarabeuvirus blattae*	Wǔchāng cockroach virus 3 (WcLFV3)
	*Scarabeuvirus dentati*	Húběi chuvirus-like virus 3 (HbCLV3)
	*Scarabeuvirus hubeiense*	Húběi chuvirus-like virus 1 (HbCLV1)
	*Scarabeuvirus lampyris*	Lampyris noctiluca chuvirus-like virus 1 (LNClV1)
	*Scarabeuvirus lishiense*	Lĭshì spider virus 1 (LsSV1)
	*Scarabeuvirus supellae*	Supella longipalpa mononega-like virus 2 (SLmlV2)
	*Scarabeuvirus trichopriae*	Trichopria drosophilae mononega-like virus (TDmlV)
*Taceavirus*	*Taceavirus wenlingense*	Wēnlǐng crustacean virus 15 (WlCV15)
*Vapochuvirus*	*Vapochuvirus trialeurodis*	Trialeurodes vaporariorum mononega-like virus 2 (TVmlV2)
**Family *Crepuscuviridae***		
*Aqualaruvirus*	*Aqualaruvirus sialis*	megalopteran chu-related virus 119 (MCrV119)
**Family *Myriaviridae***		
*Myriavirus*	*Myriavirus myriapedis*	Húběi myriapoda virus 8 (HbMV8)
**Family *Natareviridae***		
*Charybdivirus*	*Charybdivirus charybdis*	Wēnzhōu crab virus 3 (WzCV3)

Note that viruses are real objects that are assigned to concepts that are called taxa. Species, genera, families and orders are taxa.

* Taxon names are always italicized and always begin with a capital letter.

† Virus names are not italicized and are not capitalized, except if the name or a name component is a proper noun. This column lists the virus names with their correct (lack of) capitalization. Lists of viruses within a given species are provisional at this point and will likely be amended in the near future.

**Table 4.** Taxonomy of the order *Mononegavirales* (*Negarnaviricota: Haploviricotina: Monjiviricetes*) as of April 2024

Genus	Species*	Virus (abbreviation)†
**Family *Artoviridae*** [126]		
*Hexartovirus*	*Hexartovirus cirripedis*	Běihǎi barnacle virus 8 (BhBV8)
	*Hexartovirus lepeophtheiri*	Lepeophtheirus salmonis negative-stranded RNA virus 1 (LsNSRV1)
*Peropuvirus*	*Peropuvirus beihaiense*	Běihǎi rhabdo-like virus 1 (BhRLV1)
	*Peropuvirus dentati*	Húběi rhabdo-like virus 8 (HbRLV8)
	*Peropuvirus hubeiense*	Húběi rhabdo-like virus 6 (HbRLV6)
	*Peropuvirus juli*	Húběi rhabdo-like virus 5 (HbRLV5)
	*Peropuvirus lignarii*	Běihǎi rhabdo-like virus 2 (BhRLV2)
	*Peropuvirus melongenae*	Solanum melongena rhabdo-like virus (SmRLV)
	*Peropuvirus pteromali*	Pteromalus puparum negative-strand RNA virus 1 (PpNSRV1)
**Family *Bornaviridae*** [127]		
*Carbovirus*	*Carbovirus queenslandense*	jungle carpet python virus (JCPV)
	*Carbovirus tapeti*	southwest carpet python virus (SWCPV)
*Cartilovirus*	*Cartilovirus plani*	little skate bornavirus (LSBV)
*Cultervirus*	*Cultervirus electrophori*	electric eel bornavirus (EEBV)
	*Cultervirus hemicultri*	Murray-Darling carp bornavirus (MDCBV)
		Wǔhàn sharpbelly bornavirus (WhSBV)
	*Cultervirus inflati*	finepatterned puffer bornavirus (FPBV)
*Orthobornavirus*	*Orthobornavirus alphapsittaciforme*	parrot bornavirus 1 (PaBV1)
		parrot bornavirus 2 (PaBV2)
		parrot bornavirus 3 (PaBV3)
		parrot bornavirus 4 (PaBV4)
		parrot bornavirus 7 (PaBV7)
	*Orthobornavirus avisaquaticae*	aquatic bird bornavirus 1 (ABBV1)
		aquatic bird bornavirus 2 (ABBV2)
	*Orthobornavirus betapsittaciforme*	parrot bornavirus 5 (PaBV5)
	*Orthobornavirus bornaense*	Borna disease virus 1 (BoDV1)
		Borna disease virus 2 (BoDV2)
	*Orthobornavirus caenophidiae*	Caribbean watersnake bornavirus (CWBV)
		Mexican black-tailed rattlesnake bornavirus (MRBV)
	*Orthobornavirus elapsoideae*	Loveridge’s garter snake virus 1 (LGSV1)
	*Orthobornavirus estrildidae*	estrildid finch bornavirus 1 (EsBV1)
	*Orthobornavirus sciuri*	variegated squirrel bornavirus 1 (VSBV1)
	*Orthobornavirus serini*	canary bornavirus 1 (CnBV1)
		canary bornavirus 2 (CnBV2)
		canary bornavirus 3 (CnBV3)
**Family *Filoviridae*** [128]		
*Cuevavirus*	*Cuevavirus lloviuense*	Lloviu virus (LLOV)
*Dianlovirus*	*Dianlovirus menglaense*	Měnglà virus (MLAV)
*Loebevirus*	*Loebevirus percae*	Lötschberg virus (LTBV)
*Oblavirus*	*Oblavirus percae*	Oberland virus (OBLV)
*Orthoebolavirus*	*Orthoebolavirus bombaliense*	Bombali virus (BOMV)
	*Orthoebolavirus bundibugyoense*	Bundibugyo virus (BDBV)
	*Orthoebolavirus restonense*	Reston virus (RESTV)
	*Orthoebolavirus sudanense*	Sudan virus (SUDV)
	*Orthoebolavirus taiense*	Taï Forest virus (TAFV)
	*Orthoebolavirus zairense*	Ebola virus (EBOV)
*Orthomarburgvirus*	*Orthomarburgvirus marburgense*	Marburg virus (MARV)
		Ravn virus (RAVV)
*Striavirus*	*Striavirus antennarii*	Xīlǎng virus (XILV)
*Tapjovirus*	*Tapjovirus bothropis*	Tapajós virus (TAPV)
*Thamnovirus*	*Thamnovirus kanderense*	Kander virus (KNDV)
	*Thamnovirus percae*	Fiwi virus (FIWIV)
	*Thamnovirus thamnaconi*	Huángjiāo virus (HUJV)
**Family *Lispiviridae*** [129]		
*Acridvirus*	*Acridvirus hangzhouense*	Hángzhōu acrida cinerea lispivirus 1 (HzACLV1)
*Aleyavirus*	*Aleyavirus fuyangense*	Bemisia tabaci arlivirus 1 (BtAV1)
*Aleybvirus*	*Aleybvirus fuyangense*	Bemisia tabaci arlivirus 2 (BtAV2)
*Anicalvirus*	*Anicalvirus hangzhouense*	Anisopteromalus calandrae negative-strand RNA virus 2 (AcNSRV2)
	*Anicalvirus hesdarense*	hymenopteran arli-related virus OKIAV100 (HARV100)
*Anidravirus*	*Anidravirus hangzhouense*	Anisopteromalus calandrae negative-strand RNA virus 1 (AcNSRV1)
*Aranavirus*	*Aranavirus guiyangense*	Guìyáng lispivirus 1 (GyLV1)
*Aranbvirus*	*Aranbvirus guiyangense*	Guìyáng lispivirus 2 (GyLV2)
*Arlivirus*	*Arlivirus arachnae*	Lĭshì spider virus 2 (LsSV2)
	*Arlivirus hangzhouense*	Hángzhōu Scotinophara lurida lispivirus 1 (HzSLLV1)
	*Arlivirus ningboense*	Nbu stink bug virus 1 (NbuSBV1)
*Avesvirus*	*Avesvirus sinense*	Arlivirus sp. virus (ALV)
*Birfecvirus*	*Birfecvirus tibetense*	arlivirus sp. XZN142933 (ALV33)
*Copasivirus*	*Copasivirus cattienense*	Cát Tiên Hospitalitermes lispi-like virus (CHLV)
	*Copasivirus ivindoense*	isopteran arli-related virus OKIAV103 (IARV103)
	*Copasivirus manlyvaleense*	Jimsystermes virus (JIMV)
*Cybitervirus*	*Cybitervirus niederense*	coleopteran arli-related virus OKIAV107 (CARV107)
*Damravirus*	*Damravirus dentatis*	Húběi odonate virus 10 (HbOV10)
	*Damravirus fushunense*	Fǔshùn Ischnura senegalensis lispivirus 1 (FsISLV1)
*Ganiavirus*	*Ganiavirus fuyunense*	Fùyùn tick virus 1 (FTV1)
	*Ganiavirus tachengense*	Tǎchéng tick virus 6 (TcTV6)
*Hemipvirus*	*Hemipvirus scuti*	Hángzhōu Eysarcoris guttigerus lispivirus 1 (HzEGLV1)
	*Hemipvirus veri*	Hángzhōu Cletus punctiger lispivirus 1 (HzCPLV1)
*Leocovirus*	*Leocovirus coleopteris*	Húběi rhabdo-like virus 3 (HbRLV3)
*Nematovirus*	*Nematovirus wuchangense*	Wǔchāng romanomermis nematode virus 2 (WcRNV2)
*Phelinovirus*	*Phelinovirus aphidis*	hymenopteran arli-related virus OKIAV99 (HARV99)
*Rivapovirus*	*Rivapovirus aleyrodidae*	hemipteran arli-related virus OKIAV94 (HARV94)
*Sanstrivirus*	*Sanstrivirus gerridis*	Sānxiá water strider virus 4 (SxWSV4)
*Stylovirus*	*Stylovirus niederense*	strepsipteran arli-related virus OKIAV104 (SARV104)
*Supelovirus*	*Supelovirus thailandense*	blattodean arli-related virus OKIAV102 (BARV102)
*Synelinevirus*	*Synelinevirus bonnense*	hymenopteran arli-related virus OKIAV98 (HARV98)
	*Synelinevirus paranaense*	Linepithema humile rhabdo-like virus 1 (LhuRLV1)
*Usmuvirus*	*Usmuvirus newyorkense*	Amsterdam virus (AMSV)
*Xenophyvirus*	*Xenophyvirus mathesonense*	hemipteran arli-related virus OKIAV95 (HARV95)
**Family *Mymonaviridae*** [130]		
*Auricularimonavirus*	*Auricularimonavirus armillariae*	Armillaria mellea negative strand RNA virus 2 (AmNSRV2)
	*Auricularimonavirus auriculariae*	Auricularia heimuer negative-stranded RNA virus 1 (AhNRSV1)
	*Auricularimonavirus bondarzewiae*	Bondarzewia berkeleyi negative-strand RNA virus 1 (BbNSRV1)
*Botrytimonavirus*	*Botrytimonavirus alphabotrytidis*	Botrytis cinerea negative-stranded RNA virus 5 (BcNSRV5)
		Sclerotinia sclerotiorum negative-stranded RNA virus 10 (SsNSRV10)
	*Botrytimonavirus botrytidis*	Botrytis cinerea negative-stranded RNA virus 7 (BcNSRV7)
		Sclerotinia sclerotiorum negative-stranded RNA virus 11 (SsNSRV11)
	*Botrytimonavirus glycinis*	soybean leaf-associated negative-stranded RNA virus 3 (SLaNSRV3)
	*Botrytimonavirus sclerotiniae*	Sclerotinia sclerotiorum negative-stranded RNA virus 2 (SsNSRV2)
		Sclerotinia sclerotiorum negative-stranded RNA virus 2A (SsNSRV2A)
		Sclerotinia sclerotiorum negative-stranded RNA virus 4 (SsNSRV4)
		Sclerotinia sclerotiorum negative-stranded RNA virus 4A (SsNSRV4A)
*Hubramonavirus*	*Hubramonavirus fusarii*	Fusarium oxysporum mymonavirus 1 (FoMyV1)
	*Hubramonavirus golovinomycesae*	Golovinomyces cichoracearum negative-stranded RNA virus (GcNSRV1)
	*Hubramonavirus hubeiense*	Húběi rhabdo-like virus 4 (HbRLV4)
	*Hubramonavirus terrae*	H2BulkLitter1223 virus (H2BLV)
	*Hubramonavirus vitis*	grapevine-associated mononega-like virus 3 (GaMLV3)
*Lentimonavirus*	*Lentimonavirus armillariae*	Armillaria mellea negative-stranded RNA virus 1 (AmNSRV1)
	*Lentimonavirus lentinulae*	Lentinula edodes negative-stranded RNA virus 1 (LeNSRV1)
*Penicillimonavirus*	*Penicillimonavirus alphaerysiphe*	Erysiphe necator-associated negative-stranded RNA virus 2 (EnaNSRV2)
	*Penicillimonavirus alphapenicillii*	Penicillium adametzioides negative-stranded RNA virus 1 (PaNSRV1)
	*Penicillimonavirus alphaplasmoparae*	Plasmopara viticola lesion-associated mononegaambi virus 1 (PvaMV1)
	*Penicillimonavirus betaerysiphe*	Erysiphe necator-associated negative-stranded RNA virus 5 (EnaNSRV5)
	*Penicillimonavirus betapenicillii*	Penicillium glabrum negative-stranded RNA virus 1 (PgRlV1)
	*Penicillimonavirus betaplasmoparae*	Plasmopara viticola lesion-associated mononegaambi virus 2 (PvaMV2)
		Plasmopara viticola lesion-associated mononegaambi virus 4 (PvaMV4)
	*Penicillimonavirus cercosporae*	Cercospora beticola negative-stranded virus 3 (CbNSV3)
	*Penicillimonavirus deltaplasmoparae*	Plasmopara viticola lesion-associated mononegaambi virus 5 (PvaMV5)
	*Penicillimonavirus epsilonplasmoparae*	Plasmopara viticola lesion-associated mononegaambi virus 6 (PvaMV6)
	*Penicillimonavirus etaplasmoparae*	Plasmopara viticola lesion-associated mononegaambi virus 9 (PvaMV9)
	*Penicillimonavirus gammaerysiphe*	Erysiphe necator-associated negative-stranded RNA virus 6 (EnaNSRV6)
	*Penicillimonavirus gammaplasmopara*	Plasmopara viticola lesion-associated mononegaambi virus 3 (PvaMV3)
	*Penicillimonavirus kilnbarnense*	Kiln Barn virus (KBV)
	*Penicillimonavirus magnaporthe*	Magnaporthe oryzae mononegaambi virus 1 (MoMV1)
	*Penicillimonavirus zetaplasmoparae*	Plasmopara viticola lesion-associated mononegaambi virus 7 (PvaMV7)
*Phyllomonavirus*	*Phyllomonavirus culicis*	XiangYun mono-chu-like virus 7 (XYmclV7)
	*Phyllomonavirus gysingense*	Gysinge virus (GYSV)
	*Phyllomonavirus phyllospherae*	soybean leaf-associated negative-stranded RNA virus 4 (SLaNSRV4)
*Plasmopamonavirus*	*Plasmopamonavirus alphatrichodermae*	Trichoderma harzianum mononegavirus 1 (ThMV1)
	*Plasmopamonavirus betatrichodermae*	Trichoderma harzianum mononegavirus 2 (ThMV2)
	*Plasmopamonavirus erysiphe*	Erysiphe necator-associated negative-stranded RNA virus 23 (EnaNSRV23)
	*Plasmopamonavirus plasmoparae*	Plasmopara viticola lesion-associated mononegaambi virus 8 (PvaMV8)
*Rhizomonavirus*	*Rhizomonavirus mali*	apple virus B (APPVB)
*Sclerotimonavirus*	*Sclerotimonavirus alphaalternariae*	Alternaria dianthicola negative-stranded RNA virus 1 (AdNSRV1)
	*Sclerotimonavirus alphabotrytidis*	Botrytis cinerea negative-stranded RNA virus 3 (BcNSRV3)
		Sclerotinia sclerotiorum negative-stranded RNA virus 9 (SsNSRV9)
	*Sclerotimonavirus alphaclarireediae*	Sclerotinia homoeocarpa TSA contig 1 (ShTSA1)
		Sclerotinia homoeocarpa TSA contig 2 (ShTSA2)
	*Sclerotimonavirus alphaplasmoparae*	Plasmopara viticola lesion-associated mymonavirus 1 (PvaMV1)
	*Sclerotimonavirus alpharhipicephali*	Zhāngzhōu mymon tick virus 3 (ZzMtV3)
	*Sclerotimonavirus alternariae*	Alternaria tenuissima negative-stranded RNA virus 1 (AtNSRV1)
	*Sclerotimonavirus asiafusarii*	Fusarium asiaticum negative-stranded RNA virus 1 (FaNSRV1)
	*Sclerotimonavirus betaalternariae*	Alternaria tenuissima negative-stranded RNA virus 2 (AtNSRV2)
	*Sclerotimonavirus betabotrytidis*	Botrytis cinerea negative-stranded RNA virus 4 (BcNSRV4)
	*Sclerotimonavirus betaclarireediae*	Sclerotinia homoeocarpa TSA contig 3 (ShTSA3)
	*Sclerotimonavirus betaplasmoparae*	Plasmopara viticola lesion-associated mononega virus 2 (PvaMV2)
	*Sclerotimonavirus betarhipicephali*	Sānyà mymon tick virus 1 (SyMtV1)
	*Sclerotimonavirus botrytidis*	Botrytis cinerea mymonavirus 1 (BcMyV1)
		Sclerotinia sclerotiorum negative-stranded RNA virus 7 (SsNSRV7)
	*Sclerotimonavirus ceratocystis*	Sclerotimonavirus cacaofunestae (SVc)
	*Sclerotimonavirus cryphonectriae*	Cryphonectria parasitica sclerotimonavirus 1 (CpSV1)
	*Sclerotimonavirus fusarii*	Fusarium graminearum negative-stranded RNA virus 1 (FgNSRV1)
		soybean leaf-associated negative-stranded RNA virus 1 (SLaNSRV1)
	*Sclerotimonavirus illinoisense*	soybean leaf-associated negative-stranded RNA virus 2 (SLaNSRV2)
	*Sclerotimonavirus penicillii*	Penicillium cairnsense negative-stranded RNA virus 1 (PcNSRV1)
	*Sclerotimonavirus prolifusarii*	Fusarium proliferatum mymonavirus 1 (FpMV1)
	*Sclerotimonavirus sclerotiniae*	Sclerotinia sclerotiorum negative-stranded RNA virus 1 (SsNSRV1)
		Sclerotinia sclerotiorum negative-stranded RNA virus 1A (SsNSRV1A)
		Sclerotinia sclerotiorum negative-stranded RNA virus 3 (SsNSRV3)
		Sclerotinia sclerotiorum negative-stranded RNA virus 3A (SsNSRV3A)
	*Sclerotimonavirus terrae*	H4BulkLitter234 virus (H4BLV)
	*Sclerotimonavirus trichodermae*	Trichoderma harzianum negative-stranded virus 2 (ThNV2)
	*Sclerotimonavirus xinjiangense*	Xīnjiāng mymona-like virus 2 (XjMLV2)
	*Sclerotimonavirus yunnanense*	Yúnnán mymona-like virus 1 (YnMLV1)
**Family *Nyamiviridae*** [131]		
*Berhavirus*	*Berhavirus alphagirardiae*	girado virus 1 (GiV1)
	*Berhavirus beihaiense*	Běihǎi rhabdo-like virus 4 (BhRLV4)
	*Berhavirus betagirardiae*	girado virus 2 (GiV2)
	*Berhavirus gammagirardiae*	girado virus 3 (GiV3)
	*Berhavirus macrostomi*	macroli virus (MacrV)
	*Berhavirus radialis*	Běihǎi rhabdo-like virus 5 (BhRLV5)
	*Berhavirus sipunculi*	Běihǎi rhabdo-like virus 3 (BhRLV3)
*Crustavirus*	*Crustavirus beihaiense*	Běihǎi rhabdo-like virus 6 (BhRLV6)
	*Crustavirus wenlingense*	Wēnlǐng crustacean virus 12 (WlCV12)
	*Crustavirus wenzhouense*	Wēnzhōu crab virus 1 (WzCV1)
*Formivirus*	*Formivirus angliae*	Formica fusca virus 1 (FfusV1)
	*Formivirus chalybii*	hymenopteran orino-related virus OKIAV87 (HORV87)
	*Formivirus finnoniae*	Formica exsecta virus 4 (FeV4)
	*Formivirus gorytis*	hymenopteran orino-related virus OKIAV85 (HORV85)
	*Formivirus ixodis*	Ixodes ricinus orinovirus-like virus 1 (IROV1)
	*Formivirus pollinis*	Xiāngshān nyami-like virus (XnyV)
	*Formivirus solenopsi*	Solenopsis invicta virus 15 (SoINV15)
*Nyavirus*	*Nyavirus argatis*	Sekira virus (SEKRV)
	*Nyavirus gerbillisci*	Toure virus (TOUV)
	*Nyavirus midwayense*	Midway virus (MIDWV)
	*Nyavirus nyamaniniense*	Nyamanini virus (NYMV)
	*Nyavirus sanjacintoense*	San Jacinto virus (SJCV)
	*Nyavirus sierranevadaense*	Sierra Nevada virus (SNVV)
	*Nyavirus somateriae*	Jeremy Point nyavirus (JPNV)
*Orinovirus*	*Orinovirus pasiphilae*	Orinoco virus (ONCV)
	*Orinovirus sanyae*	Sānyà nyamivirus 1 (SaNyV1)
*Socyvirus*	*Socyvirus heteroderae*	soybean cyst nematode virus 1 (SbCNV1)
*Tapwovirus*	*Tapwovirus cesti*	Wēnzhōu tapeworm virus 1 (WzTWV1)
	*Tapwovirus taeniae*	taeniapi virus (TNPV)
**Family *Paramyxoviridae*** [132]		
**Subfamily *Avulavirinae***		
*Metaavulavirus*	*Metaavulavirus bangorense*	avian paramyxovirus 26 (APMV26)
	*Metaavulavirus calidris*	avian paramyxovirus 24 (APMV24)
	*Metaavulavirus delawarense*	avian paramyxovirus 8 (APMV8)
	*Metaavulavirus falklandense*	avian paramyxovirus 10 (APMV10)
	*Metaavulavirus galliense*	avian paramyxovirus 11 (APMV11)
	*Metaavulavirus hongkongense*	avian paramyxovirus 6 (APMV6)
	*Metaavulavirus japanense*	avian paramyxovirus 14 (APMV14)
	*Metaavulavirus kazakhstanense*	avian paramyxovirus 20 (APMV20)
	*Metaavulavirus kunitachiense*	avian paramyxovirus 5 (APMV5)
	*Metaavulavirus peixense*	avian paramyxovirus 15 (APMV15)
	*Metaavulavirus procarduelis*	avian paramyxovirus 27 (APMV27)
	*Metaavulavirus taiwanense*	avian paramyxovirus 22 (APMV22)
	*Metaavulavirus tennessense*	avian paramyxovirus 7 (APMV7)
	*Metaavulavirus yucaipaense*	avian paramyxovirus 2 (APMV2)
*Orthoavulavirus*	*Orthoavulavirus arenariae*	avian paramyxovirus 23 (APMV23)
	*Orthoavulavirus borisense*	Antarctic penguin virus A (APVA)
	*Orthoavulavirus italiense*	avian paramyxovirus 12 (APMV12)
	*Orthoavulavirus japanense*	avian paramyxovirus 13 (APMV13)
	*Orthoavulavirus javaense*	avian paramyxovirus 1 (APMV1)‡
	*Orthoavulavirus kopaiticense*	Antarctic penguin virus B (APVB)
	*Orthoavulavirus koreaense*	avian paramyxovirus 21 (APMV21)
	*Orthoavulavirus newyorkense*	avian paramyxovirus 9 (APMV9)
	*Orthoavulavirus oneillense*	Antarctic penguin virus C (APVC)
	*Orthoavulavirus taiwanense*	avian paramyxovirus 25 (APMV25)
	*Orthoavulavirus upoense*	avian paramyxovirus 16 (APMV16)
*Paraavulavirus*	*Paraavulavirus hongkongense*	avian paramyxovirus 4 (APMV4)
	*Paraavulavirus neophemae*	avian paramyxovirus 28 (APMV28)
	*Paraavulavirus wisconsinense*	avian paramyxovirus 3 (APMV3)
**Subfamily *Feraresvirinae***		
*Aquaparamyxovirus*	*Aquaparamyxovirus oregonense*	Pacific salmon paramyxovirus (PSPV)
	*Aquaparamyxovirus salmonis*	Atlantic salmon paramyxovirus (AsaPV)
*Ferlavirus*	*Ferlavirus reptilis*	fer-de-lance virus (FDLV)
*Respirovirus*	*Respirovirus bovis*	bovine parainfluenza virus 3 (BPIV3)
	*Respirovirus caprae*	caprine parainfluenza virus 3 (CPIV3)
	*Respirovirus henanense*	porcine parainfluenza virus 2 (PPIV2)
	*Respirovirus laryngotracheitidis*	human parainfluenza virus 1 (HPIV1)
	*Respirovirus muris*	Sendai virus (SeV)
	*Respirovirus pneumoniae*	human parainfluenza virus 3 (HPIV3)
	*Respirovirus ratufae*	giant squirrel virus (GSqV)
	*Respirovirus rupicaprae*	chamois respirovirus (ChRPV)
	*Respirovirus suis*	porcine parainfluenza virus 1 (PPIV1)
**Subfamily *Glossavirinae***		
*Cynoglossusvirus*	*Cynoglossusvirus cynoglossi*	Wēnlǐng tonguesole paramyxovirus (WTSPV)
**Subfamily *Ichthysvirinae***		
*Hoplichthysvirus*	*Hoplichthysvirus hoplichthysis*	Wēnlǐng hoplichthys paramyxovirus (WHPV)
**Subfamily *Kamposvirinae***		
*Hippocavirus*	*Hippocavirus hippocampi*	Hippocampus erectus paramyxovirus 1 (HePmV1)
**Subfamily *Metaparamyxovirinae***		
*Synodonvirus*	*Synodonvirus synodi*	Wēnlǐng triplecross lizardfish paramyxovirus (WTLPV)
**Subfamily *Orthoparamyxovirinae***		
*Bovinavirus*	*Bovinavirus bovis*	Bulang virus (BulV)
*Henipavirus*	*Henipavirus angavokelyense*	Angavokely virus (AngV)
	*Henipavirus cedarense*	Cedar virus (CedV)
	*Henipavirus ghanaense*	Okum tree virus (OTrV)
	*Henipavirus hendraense*	Hendra virus (HeV)
	*Henipavirus nipahense*	Nipah virus (NiV)
*Jeilongvirus*	*Jeilongvirus apodemi*	rodent paramyxovirus (RoPV)
	*Jeilongvirus beilongi*	Beilong virus (BeiV)
	*Jeilongvirus chaetodipodis*	chaeba virus (ChaBV)
	*Jeilongvirus eothenomysis*	Wǔfēng Eothenomys melanogaster jeilongvirus 1 (WfEmJV1)
	*Jeilongvirus gueckedouense*	memana virus (MemaV)
	*Jeilongvirus lishuiense*	Lóngquán Niviventer fulvescens jeilongvirus 2 (LqNfJV2)
	*Jeilongvirus longquanense*	Lóngquán Niviventer fulvescens jeilongvirus 1 (LqNfJV1)
	*Jeilongvirus lophuromysis*	Mount Mabu Lophuromys virus 1 (MMLV1)
	*Jeilongvirus mabuense*	Mount Mabu Lophuromys virus 2 (MMLV2)
	*Jeilongvirus mastomysis*	ninomys virus (NinYsV)
	*Jeilongvirus meliandouense*	Méliandou lophuromys virus (MeLoV)
	*Jeilongvirus merionis*	gerbil paramyxovirus (GePV)
	*Jeilongvirus microti*	Ninove microtus virus (NiMiV)
	*Jeilongvirus myodesis*	Pohorje Myodes paramyxovirus 1 (PMPV1)
	*Jeilongvirus neotomae*	neotal virus (NeTV)
	*Jeilongvirus ninovense*	ninapo virus (NinaV)
	*Jeilongvirus niviventris*	Lóngquán Niviventer niviventer jeilongvirus 1 (LqNnJV1)
	*Jeilongvirus nzerekorense*	Méliandou mastomys virus (MeMV)
	*Jeilongvirus ochotonae*	Ochotona cansus jeilongvirus (OcJV)
	*Jeilongvirus oujiangense*	Wēnzhōu Rattus norvegicus jeilongvirus 1 (WzRnJV1)
	*Jeilongvirus pajuense*	Paju Apodemus paramyxovirus 1 (PAPV1)
	*Jeilongvirus praomysis*	Méliandou praomys virus (MePV)
	*Jeilongvirus queenslandense*	J virus (JV)
	*Jeilongvirus ratti*	Wēnzhōu Rattus losea jeilongvirus 2 (WzRlJV2)
	*Jeilongvirus rungweense*	ruloma virus (RulV)
	*Jeilongvirus sichuanense*	Eothenomys eva jeilongvirus (EeJV)
	*Jeilongvirus taichungense*	Wǔfēng Rattus nitidus jeilongvirus 1 (WfRnJV1)
	*Jeilongvirus tailamense*	Tailam virus (TaiV)
	*Jeilongvirus typhlomysis*	Wǔfēng Typhlomys cinereus jeilongvirus 1 (WfTcJV1)
	*Jeilongvirus winnikense*	denotus virus (DenoV)
	*Jeilongvirus wufengense*	Wǔfēng Apodemus chevrieri jeilongvirus 1 (WfAcJV1)
	*Jeilongvirus yeoncheonense*	Paju Apodemus paramyxovirus 2 (PAPV2)
*Morbillivirus*	*Morbillivirus canis*	canine distemper virus (CDV)
	*Morbillivirus caprinae*	peste-des-petits-ruminants virus (PPRV)
	*Morbillivirus ceti*	cetacean morbillivirus (CeMV)
	*Morbillivirus felis*	feline morbillivirus (FeMV)
	*Morbillivirus hominis*	measles virus (MeV)
	*Morbillivirus myotis*	Myotis bat morbillivirus (MBaMV)
	*Morbillivirus pecoris*	rinderpest virus (RPV)
	*Morbillivirus phocae*	phocine distemper virus (PDV)
	*Morbillivirus phyllostomi*	Phyllostomus bat morbillivirus (PBaMV)
	*Morbillivirus suis*	porcine morbillivirus (PMOV)
*Narmovirus*	*Narmovirus meliandouense*	meleucus virus (meleV)
	*Narmovirus microti*	denalis virus (DenaV)
	*Narmovirus mossmanense*	Mossman virus (MossV)
	*Narmovirus myodesis*	bank vole virus 1 (BaV1)
	*Narmovirus narivaense*	Nariva virus (NarV)
	*Narmovirus ninovense*	denestis virus (DeneV)
*Parahenipavirus*	*Parahenipavirus chodsigoae*	Wǔfēng Chodsigoa smithii henipavirus 1 (WfCsHV1)
	*Parahenipavirus crocidurae*	Wǔfēng Crocidura attenuata henipavirus 1 (WfCaHV1)
	*Parahenipavirus daeryongense*	Daeryong virus (DARV)
	*Parahenipavirus gamakense*	Gamak virus (GAKV)
	*Parahenipavirus jingmenense*	Jīngmén Crocidura shantungensis henipavirus 1 (JmCsHV1)
	*Parahenipavirus langyaense*	Lángyá virus (LayV)
	*Parahenipavirus meliandouense*	melian virus (MeliV)
	*Parahenipavirus mojiangense*	Mòjiāng virus (MojV)
	*Parahenipavirus soricis*	ninorex virus (NinExV)
	*Parahenipavirus wenzhouense*	Wēnzhōu Apodemus agrarius henipavirus 1 (WzAaHV1)
	*Parahenipavirus winnikense*	denwin virus (DewV)
*Parajeilongvirus*	*Parajeilongvirus brazilense*	Diaemus bat paramyxovirus 1 (DBPV1)
	*Parajeilongvirus carolliae*	Carollia bat paramyxovirus (CBPV)
	*Parajeilongvirus comorosense*	bat paramyxovirus 16797 (BatPV1)
	*Parajeilongvirus desmodi*	Boe virus (BOEV)
	*Parajeilongvirus diaemi*	Diaemus bat paramyxovirus 2 (DBPV2)
	*Parajeilongvirus erinacei*	belerina virus (BeV)
	*Parajeilongvirus hainanense*	Hipposideros armiger paramyxovirus (HaParaV)
	*Parajeilongvirus hipposideri*	Hipposideros bat paramyxovirus (HBPV)
	*Parajeilongvirus hubeiense*	Jīngmén Miniopterus schreibersii paramyxovirus 1 (JmMsPV1)
	*Parajeilongvirus jingmenense*	Jīngmén Myotis davidii paramyxovirus 1 (JmMdPV1)
	*Parajeilongvirus madagascarense*	bat paramyxovirus 17770 (BatPV2)
	*Parajeilongvirus miniopteri*	Shaan virus (ShaV)
	*Parajeilongvirus myotis*	Wǔfēng Myotis altarium paramyxovirus 1 (WfMaPV1)
	*Parajeilongvirus pipistrelli*	piparella virus (PipaV)
	*Parajeilongvirus plecoti*	plecomyxo virus (PlecoV)
	*Parajeilongvirus rhinolophi*	Wǔfēng Rhinolophus pearsonii paramyxovirus 1 (WfRpPV1)
	*Parajeilongvirus wenzhouense*	Wēnzhōu Myotis davidii paramyxovirus 1 (WzMdPV1)
	*Parajeilongvirus zhejiangense*	Wēnzhōu Myotis laniger paramyxovirus 2 (WzMlPV2)
*Paramorbillivirus*	*Paramorbillivirus berylmysis*	Lóngquán Berylmys bowersi morbillivirus 1 (LqBbMV1)
	*Paramorbillivirus gierlense*	Gierle apodemus virus (GapV)
	*Paramorbillivirus niviventris*	Wǔfēng Niviventer fulvescens morbillivirus 1 (WfNfMV1)
	*Paramorbillivirus pueyrredonense*	Ratón oliváceo morbillivirus (RoMV)
*Salemvirus*	*Salemvirus salemense*	Salem virus (SalV)
*Tupaivirus*	*Tupaivirus tupaiae*	Tupaia paramyxovirus (TupV)
**Subfamily *Rubulavirinae***		
*Orthorubulavirus*	*Orthorubulavirus hominis*	human parainfluenza virus 4 a (HPIV4a)
		human parainfluenza virus 4b (HPIV4b)
	*Orthorubulavirus laryngotracheitidis*	human parainfluenza virus 2 (HPIV2)
	*Orthorubulavirus mammalis*	parainfluenza virus 5 (PIV5)
	*Orthorubulavirus mapueraense*	Mapuera virus (MapV)
	*Orthorubulavirus parotitidis*	mumps virus (MuV)
	*Orthorubulavirus rhinolophi*	Wǔfēng Rhinolophus sinicus rubulavirus 1 (WfRsRV1)
	*Orthorubulavirus simiae*	simian virus 41 (SV41)
	*Orthorubulavirus suis*	La Piedad Michoacán Mexico virus (LPMV)
*Pararubulavirus*	*Pararubulavirus accraense*	Achimota virus 2 (AchPV2)
	*Pararubulavirus achimotaense*	Achimota virus 1 (AchPV1)
	*Pararubulavirus cantonense*	Tuhoko virus 2 (ThkPV2)
	*Pararubulavirus eidoli*	Achimota pararubulavirus 3 (AchPV3)
	*Pararubulavirus guangdongense*	Tuhoko virus 1 (ThkPV1)
	*Pararubulavirus herveyense*	Hervey virus (HerV)
	*Pararubulavirus hongkongi*	Tuhoko virus 3 (ThkPV3)
	*Pararubulavirus menangleense*	Menangle virus (MenPV)
	*Pararubulavirus sosugaense*	Sosuga virus (SOSV)
	*Pararubulavirus teviotense*	Teviot virus (TevPV)
	*Pararubulavirus tiomanense*	Tioman virus (TioPV)
**Subfamily *Skoliovirinae***		
*Scoliodonvirus*	*Scoliodonvirus scoliodontis*	Wēnzhōu pacific spadenose shark paramyxovirus (WPSSPV)
**Family *Pneumoviridae***		
*Metapneumovirus*	*Metapneumovirus avis*	avian metapneumovirus (AMPV)
	*Metapneumovirus hominis*	human metapneumovirus (HMPV)
*Orthopneumovirus*	*Orthopneumovirus bovis*	bovine respiratory syncytial virus (BRSV)
	*Orthopneumovirus hominis*	human respiratory syncytial virus (HRSV)
	*Orthopneumovirus muris*	murine pneumonia virus (MPV)
**Family *Rhabdoviridae*** [133]		
**Subfamily *Alpharhabdovirinae***		
*Almendravirus*	*Almendravirus almendras*	Puerto Almendras virus (PTAMV)
	*Almendravirus arboretum*	Arboretum virus (ABTV)
	*Almendravirus balsa*	Balsa virus (BALV)
	*Almendravirus chico*	Rio Chico virus (RCHV)
	*Almendravirus cootbay*	Coot Bay virus (CBV)
	*Almendravirus menghai*	Menghai virus (MRV)
	*Almendravirus shanxi*	Shanxi arboretum virus (SxABTV)
	*Almendravirus xiangshan*	Xiangshan rhabdo-like virus 1 (XsRLV1)
*Alphanemrhavirus*	*Alphanemrhavirus bangkok*	Rattus tanezumi rhabdovirus 1 (RtaRV1)
	*Alphanemrhavirus sodak*	Sodak rhabdovirus 1 (SDRV1)
	*Alphanemrhavirus xingshan*	Xingshan nematode virus 4 (XsNV4)
	*Alphanemrhavirus xinzhou*	Xinzhou nematode virus 4 (XzNV4)
*Alphapaprhavirus*	*Alphapaprhavirus hubei*	Hubei lepidoptera virus 2 (HbLV2)
	*Alphapaprhavirus pararge*	Pararge aegeria rhabdovirus (PaeRV)
*Alpharicinrhavirus*	*Alpharicinrhavirus bancrofti*	Haemaphysalis bancrofti rhabdovirus (HbanRV)
	*Alpharicinrhavirus blanchseco*	Blanchseco virus (BCOV)
	*Alpharicinrhavirus bole*	Bole tick virus 2 (BlTV2)
	*Alpharicinrhavirus huanggang*	Huanggang rhabd tick virus 1 (HgRTV1)
	*Alpharicinrhavirus huangpi*	Huangpi tick virus 3 (HpTV3)
	*Alpharicinrhavirus hubei*	Hubei tick rhabdovirus 1 (HbTRV1)
	*Alpharicinrhavirus jilin*	Yanbian rhabd tick virus 1 (YbRTV1)
	*Alpharicinrhavirus nanning*	Nanning rhabd tick virus 1 (NnRTV1)
	*Alpharicinrhavirus ningxia*	Guyuan rhabd tick virus 1 (GyRTV1)
	*Alpharicinrhavirus qinghai*	Yushu rhabd tick virus 2 (YsRTV2)
	*Alpharicinrhavirus reticulatus*	Dermacentor reticulatus rhabdovirus 1 (DretRV1)
	*Alpharicinrhavirus skanevik*	Norway mononegavirus 1 (NWMV1)
	*Alpharicinrhavirus tahe*	Tahe rhabdovirus 1 (ThRV1)
	*Alpharicinrhavirus taishun*	Taishun tick virus (TsTV)
	*Alpharicinrhavirus wuhan*	Wuhan tick virus 1 (WhTV1)
	*Alpharicinrhavirus zhangjiakou*	Zhangjiakou rhabd tick virus 1 (ZjRTV1)
*Amplylivirus*	*Amplylivirus cinereus*	frog lyssa-like virus 1 (FLLV1)
	*Amplylivirus pugnax*	Boana pugnax lyssa-like virus 1 (BpugLLV1)
*Arurhavirus*	*Arurhavirus aruac*	Aruac virus (ARUV)
	*Arurhavirus inhangapi*	Inhangapi virus (INHV)
	*Arurhavirus santabarbara*	Santa Barbara virus (SBAV)
	*Arurhavirus xiburema*	Xiburema virus (XIBV)
*Barhavirus*	*Barhavirus bahia*	Bahia Grande virus (BGV)
		Harlingen virus (HARV)
	*Barhavirus muir*	Muir Springs virus (MSV)
*Caligrhavirus*	*Caligrhavirus caligus*	Caligus rogercresseyi rhabdovirus (CrogRV)
	*Caligrhavirus lepeophtheirus*	Lepeophtheirus salmonis rhabdovirus 127 (LsalRV127)
	*Caligrhavirus salmonlouse*	Lepeophtheirus salmonis rhabdovirus 9 (LsalRV9)
*Cetarhavirus*	*Cetarhavirus laganorhynchus*	dolphin rhabdovirus (DRV)
	*Cetarhavirus phocoena*	harbour porpoise rhabdovirus (HPRV)
*Curiovirus*	*Curiovirus curionopolis*	Curionopolis virus (CURV)
	*Curiovirus iriri*	Iriri virus (IRIRV)
	*Curiovirus itacaiunas*	Itacaiunas virus (ITAV)
	*Curiovirus rochambeau*	Rochambeau virus (RBUV)
*Ephemerovirus*	*Ephemerovirus adelaide*	Adelaide River virus (ARV)
	*Ephemerovirus berrimah*	Berrimah virus (BRMV)
	*Ephemerovirus febris*	bovine ephemeral fever virus (BEFV)
	*Ephemerovirus guangdong*	porcine ephemerovirus 2 (PoEV2)
	*Ephemerovirus hayes*	Hayes Yard virus (HYV)
	*Ephemerovirus henan*	porcine ephemerovirus 1 (PoEV1)
	*Ephemerovirus huanggang*	Huanggang rhabd tick virus 2 (HgRTV2)
	*Ephemerovirus kent*	New Kent County virus (NKCV)
	*Ephemerovirus kimberley*	Kimberley virus (KIMV)
		Malakal virus (MALV)
	*Ephemerovirus koolpinyah*	Koolpinyah virus (KOOLV)
	*Ephemerovirus kotonkan*	kotonkan virus (KOTV)
	*Ephemerovirus obodhiang*	Obodhiang virus (OBOV)
	*Ephemerovirus puchong*	Puchong virus (PUCV)
	*Ephemerovirus yata*	Yata virus (YATV)
*Hapavirus*	*Hapavirus bangoran*	Bangoran virus (BGNV)
	*Hapavirus flanders*	Flanders virus (FLAV)
	*Hapavirus graylodge*	Grey Lodge virus (GLOV)
	*Hapavirus hartpark*	Hart Park virus (HPV)
	*Hapavirus holmes*	Holmes Jungle virus (HOJV)
	*Hapavirus joinjakaka*	Joinjakaka virus (JOIV)
	*Hapavirus kamese*	Kamese virus (KAMV)
	*Hapavirus lajoya*	La Joya virus (LJV)
	*Hapavirus landjia*	Landjia virus (LANV; LJAV)
	*Hapavirus manitoba*	Manitoba virus (MANV; MNTBV)
	*Hapavirus marco*	Marco virus (MCOV)
	*Hapavirus mosqueiro*	Mosqueiro virus (MQOV)
	*Hapavirus mossuril*	Mossuril virus (MOSV)
	*Hapavirus ngaingan*	Ngaingan virus (NGAV)
	*Hapavirus ord*	Ord River virus (ORV)
	*Hapavirus parry*	Parry Creek virus (PCV)
	*Hapavirus porton*	Porton virus (PORV)
	*Hapavirus wongabel*	Wongabel virus (WONV)
*Ledantevirus*	*Ledantevirus barur*	Barur virus (BARV)
	*Ledantevirus bughendera*	Bughendera virus (BUGV)
	*Ledantevirus elgon*	Mount Elgon bat virus (MEBV)
	*Ledantevirus fikirini*	Fikirini virus (FKRV)
	*Ledantevirus fukuoka*	Fukuoka virus (FUKV)
	*Ledantevirus kanyawara*	Kanyawara virus (KYAV)
	*Ledantevirus kern*	Kern Canyon virus (KCV)
	*Ledantevirus keuraliba*	Keuraliba virus (KEUV)
	*Ledantevirus kolente*	Kolente virus (KOLEV)
	*Ledantevirus kumasi*	Kumasi rhabdovirus (KRV)
	*Ledantevirus ledantec*	Le Dantec virus (LDV)
	*Ledantevirus longquan*	Longquan Niviventer coninga ledantevirus 1 (LNcoLV1)
	*Ledantevirus nishimuro*	Nishimuro virus (NISV)
	*Ledantevirus nkolbisson*	Nkolbisson virus (NKOV)
	*Ledantevirus oita*	Oita virus (OITAV)
	*Ledantevirus taiyi*	Taiyi bat virus (TYBV)
	*Ledantevirus tongren*	Tongren rhabd tick virus 2 (TrRTV2)
	*Ledantevirus vaprio*	Vaprio virus (VAPV)
	*Ledantevirus wenzhou*	Wenzhou Rhinolophus pusillus ledantevirus 1 (WRpuLV1)
	*Ledantevirus wuhan*	Wuhan louse fly virus 5 (WLFV5)
	*Ledantevirus yongjia*	Yongjia tick virus 2 (YTV2)
*Lostrhavirus*	*Lostrhavirus alxa*	Alxa tick rhabdovirus (ATRV)
	*Lostrhavirus hyalomma*	Xinjiang tick rhabdovirus (XjTRV)
	*Lostrhavirus lonestar*	lone star tick rhabdovirus (LITRV)
*Lyssavirus*	*Lyssavirus aravan*	Aravan virus (ARAV)
	*Lyssavirus australis*	Australian bat lyssavirus (ABLV)
	*Lyssavirus bokeloh*	Bokeloh bat lyssavirus (BBLV)
	*Lyssavirus caucasicus*	West Caucasian bat virus (WCBV)
	*Lyssavirus duvenhage*	Duvenhage virus (DUVV)
	*Lyssavirus formosa*	Taiwan bat lyssavirus (TWBLV)
	*Lyssavirus gannoruwa*	Gannoruwa bat lyssavirus (GBLV)
	*Lyssavirus hamburg*	European bat lyssavirus 1 (EBLV1)
	*Lyssavirus helsinki*	European bat lyssavirus 2 (EBLV2)
	*Lyssavirus ikoma*	Ikoma lyssavirus (IKOV)
	*Lyssavirus irkut*	Irkut virus (IRKV)
	*Lyssavirus khujand*	Khujand virus (KHUV)
	*Lyssavirus kotalahti*	Kotalahti bat lyssavirus (KBLV)
	*Lyssavirus lagos*	Lagos bat virus (LBV)
	*Lyssavirus lleida*	Lleida bat lyssavirus (LLEBV)
	*Lyssavirus mokola*	Mokola virus (MOKV)
	*Lyssavirus rabies*	rabies virus (RABV)
	*Lyssavirus shimoni*	Shimoni bat virus (SHIBV)
*Merhavirus*	*Merhavirus formosus*	Formosus virus (FORMV)
	*Merhavirus hattula*	Hattula rhabdovirus (HTTRV)
	*Merhavirus inari*	Inari rhabdovirus (INARV)
	*Merhavirus merida*	Merida virus (MERDV)
	*Merhavirus tritaeniorhynchus*	Culex tritaeniorhynchus rhabdovirus (CTRV)
*Mousrhavirus*	*Mousrhavirus moussa*	Moussa virus (MOUV)
*Ohlsrhavirus*	*Ohlsrhavirus adumi*	Adumi virus (ADUMV)
	*Ohlsrhavirus angeles*	Culex rhabdo-like virus Los Angeles (CRLVLA)
	*Ohlsrhavirus culex*	Culex rhabdo-like virus (CRLV)
	*Ohlsrhavirus lobeira*	Lobeira virus (LOBV)
	*Ohlsrhavirus northcreek*	North Creek virus (NORCV)
	*Ohlsrhavirus ohlsdorf*	Ohlsdorf virus (OHLDV)
	*Ohlsrhavirus pseudovishnui*	Culex pseudovishnui rhabdo-like virus (CpRLV)
	*Ohlsrhavirus riverside*	riverside virus (RISV)
	*Ohlsrhavirus tongilchon*	Tongilchon virus 1 (TCHV1)
*Perhabdovirus*	*Perhabdovirus anguilla*	eel virus American (EVA)
		eel virus European X (EVEX)
	*Perhabdovirus leman*	Leman virus (LeRV)
	*Perhabdovirus perca*	perch rhabdovirus (PRV)
	*Perhabdovirus trutta*	lake trout rhabdovirus (LTRV)
*Replylivirus*	*Replylivirus allogus*	anole lyssa-like virus 1 (ALLV1)
*Sawgrhavirus*	*Sawgrhavirus connecticut*	Connecticut virus (CNTV)
	*Sawgrhavirus longisland*	Long Island tick rhabdovirus (LITRV)
	*Sawgrhavirus minto*	New Minto virus (NMV)
	*Sawgrhavirus sawgrass*	Sawgrass virus (SAWV)
*Scophrhavirus*	*Scophrhavirus chanodychthys*	Wuhan redfin culter dimarhabdovirus (WhRFCRV)
	*Scophrhavirus maximus*	Scophthalmus maximus rhabdovirus (SMRV)
*Sigmavirus*	*Sigmavirus affinis*	Drosophila affinis sigmavirus (DaffSV)
	*Sigmavirus ananassae*	Drosophila ananassae sigmavirus (DanaSV)
	*Sigmavirus capitata*	Ceratitis capitata sigmavirus (CcapSV)
	*Sigmavirus domestica*	Wuhan fly virus 2 (WhFV2)
	*Sigmavirus hippoboscid*	Wuhan louse fly virus 9 (WhLFV9)
	*Sigmavirus hubei*	Hubei diptera virus 9 (HbDV9)
	*Sigmavirus immigrans*	Drosophila immigrans sigmavirus (DimmSV)
	*Sigmavirus jopcycgri*	Jopcycgri virus 1 (JPCGV1)
	*Sigmavirus lousefly*	Wuhan louse fly virus 10 (WhLFV10)
	*Sigmavirus melanogaster*	Drosophila melanogaster sigmavirus (DmelSV)
	*Sigmavirus muscina*	Muscina stabulans sigmavirus (MstaSV)
	*Sigmavirus myga*	Hubei diptera virus 10 (HbDV10)
	*Sigmavirus obscura*	Drosophila obscura sigmavirus (DobsSV)
	*Sigmavirus shayang*	Shayang fly virus 2 (SyFV2)
	*Sigmavirus sichuan*	Apis rhabdovirus 3 (ApRV3)
	*Sigmavirus sturtevanti*	Drosophila sturtevanti sigmavirus (DstuSV)
	*Sigmavirus tristis*	Drosophila tristis sigmavirus (DtriSV)
	*Sigmavirus tryoni*	Bactrocera tryoni rhabdovirus 1 (BtryRV1)
	*Sigmavirus tuva*	Aksy-Durug Melophagus sigmavirus (ADMSV)
	*Sigmavirus wuhan*	Wuhan house fly virus 1 (WhHFV1)
	*Sigmavirus ying*	Hubei dimarhabdovirus 1 (HbDRV1)
	*Sigmavirus yushu*	Yushu rhabdovirus (YsRV)
*Siniperhavirus*	*Siniperhavirus chuatsi*	Chinese rice-field eel rhabdovirus (CrERV)
		hybrid snakehead rhabdovirus (HSHRV)
		Micropterus salmoides rhabdovirus (MSRV)
		Siniperca chuatsi rhabdovirus (SCRV)
	*Siniperhavirus zoarces*	eelpout rhabdovirus (EPRV)
*Sprivivirus*	*Sprivivirus cyprinus*	spring viremia of carp virus (SVCV)
	*Sprivivirus esox*	grass carp rhabdovirus (GrCRV)
		pike fry rhabdovirus (PFRV)
		tench rhabdovirus (TenRV)
*Sripuvirus*	*Sripuvirus almpiwar*	Almpiwar virus (ALMV)
	*Sripuvirus chaco*	Chaco virus (CHOV)
	*Sripuvirus charleville*	Charleville virus (CHVV)
	*Sripuvirus cuiaba*	Cuiaba virus (CUIV)
	*Sripuvirus hainan*	Hainan black-spectacled toad rhabdovirus (HnBSTRV)
	*Sripuvirus madureira*	Sena Madureira virus (SMV)
	*Sripuvirus niakha*	Niakha virus (NIAV)
	*Sripuvirus sripur*	Sripur virus (SRIV)
*Sunrhavirus*	*Sunrhavirus alexandria*	Burg el Arab virus (BEAV)
	*Sunrhavirus bimbo*	Bimbo virus (BBOV)
	*Sunrhavirus boteke*	Boteke virus (BOTV)
	*Sunrhavirus dillard*	Dillard’s Draw virus (DDRV)
	*Sunrhavirus garba*	Garba virus (GARV)
	*Sunrhavirus harrison*	Harrison Dam virus (HARDV)
	*Sunrhavirus kolongo*	Kolongo virus (KOLV)
	*Sunrhavirus kwatta*	Kwatta virus (KWAV)
	*Sunrhavirus matariya*	Matariya virus (MTYV)
	*Sunrhavirus nasoule*	Nasoule virus (NASV)
	*Sunrhavirus oakvale*	Oak Vale virus (OVV)
	*Sunrhavirus ouango*	Ouango virus (OUAV)
	*Sunrhavirus sandjimba*	Sandjimba virus (SJAV)
	*Sunrhavirus sunguru*	Sunguru virus (SUNV)
	*Sunrhavirus walkabout*	Walkabout Creek virus (WACV)
*Thriprhavirus*	*Thriprhavirus intonsa*	Hangzhou Frankliniella intonsa rhabdovirus 1 (HFinRV1)
	*Thriprhavirus tabaci*	Thrips tabaci-associated dimarhabdovirus 1 (TtaDRV1)
*Tibrovirus*	*Tibrovirus alphaekpoma*	Ekpoma virus 1 (EKV1)
	*Tibrovirus beatrice*	Beatrice Hill virus (BHV)
	*Tibrovirus betaekpoma*	Ekpoma virus 2 (EKV2)
	*Tibrovirus coastal*	Coastal Plains virus (CPV)
	*Tibrovirus congo*	Bas-Congo virus (BASV)
	*Tibrovirus mundri*	Mundri virus (MUNV)
	*Tibrovirus sweetwater*	Sweetwater Branch virus (SWBV)
	*Tibrovirus tibrogargan*	Bivens Arm virus (BAV)
		Tibrogargan virus (TIBV)
*Tupavirus*	*Tupavirus durham*	Durham virus (DURV)
	*Tupavirus incomtus*	tupavirus SB8301 (TUPVSB)
	*Tupavirus klamath*	Klamath virus (KLAV)
	*Tupavirus laniger*	Wenzhou Myotis laniger tupavirus 1 (WMlaTV1)
	*Tupavirus pearsonii*	Wufeng Rhinolophus pearsonii tupavirus 1 (WRpeTV1)
	*Tupavirus stheno*	bat tupavirus BS1 (BtTVBS1)
	*Tupavirus stoliczkanus*	bat tupavirus BS2 (BtTVBS2)
	*Tupavirus tupaia*	tupaia rhabdovirus (TUPV)
*Uniorhavirus*	*Uniorhavirus killamcar*	killamcar virus 1 (KILLV1)
*Vesiculovirus*	*Vesiculovirus alagoas*	vesicular stomatitis Alagoas virus (VSAV)
	*Vesiculovirus bogdanovac*	Yug Bogdanovac virus (YBV)
	*Vesiculovirus carajas*	Carajás virus (CJSV)
	*Vesiculovirus chandipura*	Chandipura virus (CHPV)
	*Vesiculovirus cocal*	Cocal virus (COCV)
	*Vesiculovirus eptesicus*	American bat vesiculovirus (ABVV)
	*Vesiculovirus indiana*	vesicular stomatitis Indiana virus (VSIV)
	*Vesiculovirus isfahan*	Isfahan virus (ISFV)
	*Vesiculovirus jurona*	Jurona virus (JURV)
	*Vesiculovirus malpais*	Malpais Spring virus (MSPV)
	*Vesiculovirus maraba*	Maraba virus (MARAV)
	*Vesiculovirus mediterranean*	Mediterranean bat virus (MBV)
	*Vesiculovirus mejal*	Mejal virus (MEJV)
	*Vesiculovirus morreton*	Morreton virus (MORV)
	*Vesiculovirus newjersey*	vesicular stomatitis New Jersey virus (VSNJV)
	*Vesiculovirus perinet*	Perinet virus (PERV)
	*Vesiculovirus piry*	Piry virus (PIRYV)
	*Vesiculovirus radi*	Radi virus (RADV)
	*Vesiculovirus rhinolophus*	Jinghong bat virus (JhBV)
		Qiongzhong bat virus (QZBV)
	*Vesiculovirus wufeng*	Wufeng Myotis altarium vesiculovirus 1 (WMalVV1)
	*Vesiculovirus yinshui*	Yinshui bat virus (YSBV)
*Zarhavirus*	*Zarhavirus zahedan*	Zahedan rhabdovirus (ZARV)
**Subfamily *Betarhabdovirinae***		
*Alphagymnorhavirus*	*Alphagymnorhavirus abietis*	Abies virus 1 (AbiV1)
	*Alphagymnorhavirus amentotaxi*	Amentotaxus virus 1 (AmeV1)
	*Alphagymnorhavirus cupressi*	Cupressus virus 1 (CupV1)
	*Alphagymnorhavirus piceae*	Picea virus 1 (PicV1)
	*Alphagymnorhavirus pinibanksiae*	Pinus banksiana virus 1 (PiBanV1)
	*Alphagymnorhavirus piniflexilis*	Pinus flexilis virus 1 (PiFleV1)
	*Alphagymnorhavirus piniyunannensis*	Pinis yunnanensis virus 1 (PiYunV1)
	*Alphagymnorhavirus sciadopitysis*	Sciadopitys virus 1 (SciV1)
	*Alphagymnorhavirus taxi*	Taxus virus 1 (TaxV1)
*Alphanucleorhabdovirus*	*Alphanucleorhabdovirus agavis*	Agave tequilana virus 1 (ATV1)
	*Alphanucleorhabdovirus artemisiae*	Artemisia capillaris nucleorhabdovirus 1 (ArtCaNV1)
	*Alphanucleorhabdovirus colocasiae*	taro vein chlorosis virus (TaVCV)
	*Alphanucleorhabdovirus constrictae*	constricta yellow dwarf virus (CYDV)
	*Alphanucleorhabdovirus joa*	joa yellow blotch-associated virus (JYBaV)
	*Alphanucleorhabdovirus lycopersici*	tomato alphanucleorhabdovirus 1 (TARV1)
	*Alphanucleorhabdovirus maydis*	maize mosaic virus (MMV)
	*Alphanucleorhabdovirus melongenae*	eggplant mottled dwarf virus (EMDV)
	*Alphanucleorhabdovirus morogoromaydis*	Morogoro maize-associated virus (MmaV)
	*Alphanucleorhabdovirus oryzae*	rice transitory yellowing virus (RTYV)
		rice yellow stunt virus (RYSV)
	*Alphanucleorhabdovirus physostegiae*	Physostegia chlorotic mottle virus (PhCMoV)
	*Alphanucleorhabdovirus pruni*	peach virus 1 (PeV1)
	*Alphanucleorhabdovirus tritici*	wheat yellow striate virus (WYSV)
	*Alphanucleorhabdovirus tuberosum*	potato yellow dwarf virus (PYDV)
	*Alphanucleorhabdovirus xinjianensis*	Xinjiang alphanucleorhabdovirus (XARV)
	*Alphanucleorhabdovirus zeairanense*	maize Iranian mosaic virus (MIMV)
*Betagymnorhavirus*	*Betagymnorhavirus torreyae*	Torreya virus 1 (TorV1)
*Betanucleorhabdovirus*	*Betanucleorhabdovirus alphalycopersici*	tomato betanucleorhabdovirus 1 (TBRV1)
	*Betanucleorhabdovirus asclepiadis*	Asclepias syriaca virus 2 (AscSyV2)
	*Betanucleorhabdovirus bacopae*	Bacopa monnieri virus 2 (BmV2)
	*Betanucleorhabdovirus betalycopersici*	tomato betanucleorhabdovirus 2 (TBRV2)
	*Betanucleorhabdovirus cardamomi*	cardamom vein clearing virus (CdVCV)
	*Betanucleorhabdovirus cnidii*	Cnidium virus 1 (CnV1)
	*Betanucleorhabdovirus daturae*	Datura yellow vein virus (DYVV)
	*Betanucleorhabdovirus loti*	birds-foot trefoil-associated virus (BFTV)
	*Betanucleorhabdovirus mali*	apple rootstock virus A (ApRVA)
	*Betanucleorhabdovirus medicagonis*	alfalfa-associated nucleorhabdovirus (AaNV)
	*Betanucleorhabdovirus picridis*	Picris betanucleorhabdvirus 1 (PBRV1)
	*Betanucleorhabdovirus plectranthi*	Plectranthus aromaticus virus 1 (PleArV1)
	*Betanucleorhabdovirus retesonchi*	Sonchus yellow net virus (SYNV)
	*Betanucleorhabdovirus rhododendri*	Rhododendon delavayi virus 1 (RhoDeV1)
	*Betanucleorhabdovirus ribes*	blackcurrant-associated rhabdovirus (BcaRV)
	*Betanucleorhabdovirus taraxi*	Taraxacum betanucleorhabdvirus 1 (TarBRV1)
	*Betanucleorhabdovirus venasonchi*	sowthistle yellow vein virus (SYVV)
	*Betanucleorhabdovirus zanthoxyli*	Zhuye pepper nucleorhabdovirus (ZPNRV)
*Cytorhabdovirus*	*Cytorhabdovirus actinidiae*	Actinidia virus D (AcVD)
	*Cytorhabdovirus alphatrifolii*	Trifolium pratense virus A (TpVA)
	*Cytorhabdovirus alphawuhaninsectum*	Wuhan insect virus 4 (WuIV4)
	*Cytorhabdovirus anthurii*	Anthurium amnicola virus 1 (AntAmV1)
	*Cytorhabdovirus asclepiadis*	Asclepias syriaca virus 1 (AscSyV1)
	*Cytorhabdovirus bacopae*	Bacopa monnieri virus 1 (BmV1)
	*Cytorhabdovirus bemisiae*	Bemisia tabaci-associated virus 1 (BeTaV1)
	*Cytorhabdovirus betafragariae*	strawberry virus 2 (StrV2)
	*Cytorhabdovirus betarosae*	rose-associated cytorhabdovirus (RaCV)
	*Cytorhabdovirus betatrifolii*	Trifolium pratense virus B (TpVB)
	*Cytorhabdovirus betawuhaninsectum*	Wuhan insect virus 5 (WuIV5)
	*Cytorhabdovirus brassicae*	broccoli necrotic yellows virus (BNYV)
	*Cytorhabdovirus brassicicolae*	cabbage cytorhabdovirus 1 (CcyV1)
	*Cytorhabdovirus broussonetiae*	paper mulberry mosaic-associated virus (PmuMaV)
	*Cytorhabdovirus caricae*	papaya virus E (PpVE)
	*Cytorhabdovirus chrysanthemi*	chrysanthemum yellow dwarf-associated virus (CYDaV)
	*Cytorhabdovirus colocasiae*	Colocasia bobone disease-associated virus (CBDaV)
	*Cytorhabdovirus cucurbitae*	cucurbit cytorhabdovirus 1 (CuCV1)
	*Cytorhabdovirus daphnis*	daphne virus 1 (DV1)
	*Cytorhabdovirus festucae*	Festuca leaf streak virus (FLSV)
	*Cytorhabdovirus flaviyerbamate*	yerba mate chlorosis-associated virus (YmCaV)
	*Cytorhabdovirus fragariae*	strawberry-associated virus 1 (SaV1)
		strawberry virus 1 (StrV1)
	*Cytorhabdovirus fragariarugosus*	strawberry crinkle virus (SCV)
	*Cytorhabdovirus gammawuhaninsectum*	Wuhan insect virus 6 (WuIV6)
	*Cytorhabdovirus glehniae*	Glehnia littoralis virus 1 (GlLV1)
	*Cytorhabdovirus glycinis*	soybean blotchy mosaic virus (SbBMV)
	*Cytorhabdovirus gramineae*	northern cereal mosaic virus (NCMV)
	*Cytorhabdovirus hordei*	barley yellow striate mosaic virus (BYSMV)
	*Cytorhabdovirus hyptisis*	Hyptis latent virus (HpLV)
	*Cytorhabdovirus kenyatuberosum*	Kenyan potato cytorhabdovirus (KePCyV)
	*Cytorhabdovirus lactucamaculante*	lettuce yellow mottle virus (LYMoV)
	*Cytorhabdovirus lactucanecante*	lettuce necrotic yellows virus (LNYV)
	*Cytorhabdovirus lycopersici*	tomato yellow mottle-associated virus (TYMaV)
	*Cytorhabdovirus maydis*	maize-associated cytorhabdovirus (MaCV)
	*Cytorhabdovirus maysflavostriatis*	maize yellow striate virus (MYSV)
	*Cytorhabdovirus medicagonis*	alfalfa dwarf virus (ADV)
	*Cytorhabdovirus nymphaeae*	Nymphaea alba virus 1 (NymAV1)
	*Cytorhabdovirus orchidaceae*	Gymnadenia densiflora virus 1 (GymDenV1)
	*Cytorhabdovirus oryzae*	rice stripe mosaic virus (RSMV)
	*Cytorhabdovirus pastinacae*	Pastinaca cytorhabdovirus 1 (PaCRV1)
	*Cytorhabdovirus persimmon*	persimmon virus A (PeVA)
	*Cytorhabdovirus pogostemi*	Patchouly chlorosis-associated cytorhabdovirus (PcaCV)
	*Cytorhabdovirus ribes*	blackcurrant rhabdovirus 2 (BCRV2)
	*Cytorhabdovirus rosae*	rose virus R (RVR)
	*Cytorhabdovirus rubus*	raspberry vein chlorosis virus (RVCV)
	*Cytorhabdovirus rudbeckiae*	Rudbeckia virus 1 (RudV1)
	*Cytorhabdovirus sambuci*	Sambucus virus 1 (SaV1)
	*Cytorhabdovirus sonchi*	Sonchus virus (SonV)
	*Cytorhabdovirus tagetis*	Tagetes erecta virus 1 (TaEV1)
	*Cytorhabdovirus taraxaci*	Taraxacum cytorhabdovirus 1 (TCRV1)
	*Cytorhabdovirus tiliae*	Tilia cytorhabdovirus 1 (TiCRV1)
	*Cytorhabdovirus trachyspermi*	Trachyspermum ammi virus 1 (TrAV1)
	*Cytorhabdovirus trichosanthei*	Trichosanthes-associated rhabdovirus 1 (TrARV1)
	*Cytorhabdovirus tritici*	wheat American striate mosaic virus (WASMV)
	*Cytorhabdovirus yerbamate*	yerba mate virus A (YmVA)
*Deltanucleorhabdovirus*	*Deltanucleorhabdovirus fragariae*	strawberry virus 3 (StrV3)
	*Deltanucleorhabdovirus medicagonis*	Medicago sativa virus 1 (MSV1)
*Dichorhavirus*	*Dichorhavirus australis*	citrus bright spot virus (CiBSV)
	*Dichorhavirus citri*	citrus chlorotic spot virus (CiCSV)
	*Dichorhavirus clerodendri*	Clerodendrum chlorotic spot virus (ClCSV)
	*Dichorhavirus coffeae*	coffee ringspot virus (CoRSV)
	*Dichorhavirus leprosis*	citrus leprosis virus N (CiLVN)
	*Dichorhavirus orchidaceae*	orchid fleck virus (OFV)
*Gammanucleorhabdovirus*	*Gammanucleorhabdovirus cerealis*	cereal chlorotic mottle virus (CCMoV)
	*Gammanucleorhabdovirus maydis*	maize fine streak virus (MFSV)
*Varicosavirus*	*Varicosavirus aconiti*	Aconitum virus 1 (AcoV1)
	*Varicosavirus allii*	Allium angulosum virus 1 (AanV1)
	*Varicosavirus alopecuri*	Alopecurus myosuroides varicosavirus 1 (AMVV1)
	*Varicosavirus alphabrassicae*	Brassica virus 1 (BrV1)
	*Varicosavirus aperae*	Apera virus 1 (ApeV1)
	*Varicosavirus aponogeti*	Aponogeton virus 1 (ApoV1)
	*Varicosavirus arceuthobii*	Arceuthobium virus 8 (ArcV8)
	*Varicosavirus artemisiae*	Artemisia virus 1 (ArtV1)
	*Varicosavirus asclepiadis*	Asclepias syriaca virus 3 (AscSyV3)
	*Varicosavirus betabrassicae*	Brassica virus 2 (BrV2)
	*Varicosavirus caladeniae*	Caladenia virus 1 (CalV1)
	*Varicosavirus centaurea*	Centaurea virus 1 (CenV1)
	*Varicosavirus cucumis*	Cucumis virus 1 (CucV1)
	*Varicosavirus didymochlaenae*	Didymochlaena virus 1 (DidV1)
	*Varicosavirus erysimi*	Erysimum virus 1 (EryV1)
	*Varicosavirus frullaniae*	Frullania virus 1 (FruV1)
	*Varicosavirus guizotiae*	Guizotia virus 1 (GuiV1)
	*Varicosavirus holci*	Holcus virus 1 (HolV1)
	*Varicosavirus ipomoeae*	morning glory varicosavirus (MGVV)
	*Varicosavirus lactucae*	lettuce big-vein-associated virus (LBVaV)
	*Varicosavirus leucanthemi*	Leucanthemum virus 1 (LeuV1)
	*Varicosavirus lolii*	Lolium perenne virus 1 (LoPV1)
	*Varicosavirus luffae*	Luffa virus 1 (LufV1)
	*Varicosavirus melampyri*	Melampyrum roseum virus 1 (MelRoV1)
	*Varicosavirus meliloti*	Melilotus virus 1 (MelV1)
	*Varicosavirus monocleae*	Monoclea gottschei varicosa-like virus (MgVV)
	*Varicosavirus penniseti*	Pennisetum virus 1 (PenV1)
	*Varicosavirus primulae*	Primula virus 1 (PriV1)
	*Varicosavirus ranunculi*	Ranunculus virus 1 (RanV1)
	*Varicosavirus raphani*	Raphanus virus 1 (RapV1)
	*Varicosavirus ribes*	Ribes virus 1 (RibV1)
	*Varicosavirus silenis*	Silene virus 1 (SilV1)
	*Varicosavirus streptoglossae*	Streptoglossa virus 1 (StrV1)
	*Varicosavirus tanaceti*	Tanacetum virus 1 (TanV1)
	*Varicosavirus thrysopteris*	tree fern varicosa-like virus (TfVV)
	*Varicosavirus treubiae*	Treubia virus 1 (TreV1)
	*Varicosavirus trifolii*	red clover-associated varicosavirus (RcaVV)
	*Varicosavirus tritici*	Triticum virus 1 (TriV1)
	*Varicosavirus vincetoxici*	Vincetoxicum virus 1 (VinV1)
	*Varicosavirus vitis*	Vitis varicosavirus (VVV)
	*Varicosavirus xinjiangense*	Xinjiang varicosavirus (XVV)
	*Varicosavirus zea*	Zea virus 1 (ZeaV1)
	*Varicosavirus zosterae*	Zostera-associated varicosavirus 1 (ZaVV1)
**Subfamily *Deltarhabdovirinae***		
*Alphacrustrhavirus*	*Alphacrustrhavirus wenling*	Wenling crustacean virus 10 (WlCV10)
	*Alphacrustrhavirus zhejiang*	Wenling crustacean virus 11 (WlCV11)
*Alphadrosrhavirus*	*Alphadrosrhavirus hubei*	Wuhan house fly virus 2 (WhHFV2)
	*Alphadrosrhavirus shayang*	Shayang fly virus 3 (SyFV3)
*Alphahymrhavirus*	*Alphahymrhavirus cinereus*	hymenopteran rhabdo-related virus 38 (HyRRV38)
	*Alphahymrhavirus distinguendus*	Lariophagus distinguendus negative-strand RNA virus 1 (LdNSRV1)
	*Alphahymrhavirus hirtum*	hymenopteran rhabdo-related virus 109 (HyRRV109)
	*Alphahymrhavirus neglectus*	Lasius neglectus virus 2 (LnegV2)
	*Alphahymrhavirus radians*	hymenopteran rhabdo-related virus 46 (HyRRV46)
	*Alphahymrhavirus xiangshan*	Xiangshan rhabdo-like virus 3 (XsRLV3)
*Betahymrhavirus*	*Betahymrhavirus austriaca*	hymenopteran rhabdo-related virus 23 (HyRRV23)
	*Betahymrhavirus heterodontonyx*	hymenopteran rhabdo-related virus 24 (HyRRV24)
	*Betahymrhavirus xiangshan*	Xiangshan rhabdo-like virus 4 (XsRLV4)
*Betanemrhavirus*	*Betanemrhavirus hubei*	Hubei rhabdo-like virus 9 (HbRLV9)
	*Betanemrhavirus shayang*	Shayang ascaridia galli virus 2 (SyAGV2)
*Betapaprhavirus*	*Betapaprhavirus frugiperda*	Spodoptera frugiperda rhabdovirus (SfruRV)
	*Betapaprhavirus sylvina*	lepidopteran rhabdo-related virus 34 (LeRRV34)
*Betaricinrhavirus*	*Betaricinrhavirus chimay*	Chimay rhabdovirus (CRV)
	*Betaricinrhavirus mudanjiang*	Mudanjiang rhabd tick virus 1 (MjRTV1)
	*Betaricinrhavirus scapularis*	blacklegged tick rhabdovirus 1 (BLTRV1)
	*Betaricinrhavirus tongren*	Tongren rhabd tick virus 1 (TrRTV1)
	*Betaricinrhavirus yanbian*	Yanbian rhabd tick virus 3 (YbRTV3)
*Gammahymrhavirus*	*Gammahymrhavirus cerana*	Apis rhabdovirus 4 (ApRV4)
	*Gammahymrhavirus longicaudata*	Diachasmimorpha longicaudata rhabdovirus (DlonRV)
	*Gammahymrhavirus mellifera*	Apis rhabdovirus 5 (ApRV5)
*Gammaricinrhavirus*	*Gammaricinrhavirus fuyun*	Fuyun tick rhabdovirus (FyTRV)
	*Gammaricinrhavirus tacheng*	Tacheng tick virus 7 (TcTV7)
*Primrhavirus*	*Primrhavirus atrato*	Atrato rhabdo-like virus 3 (AtRLV3)
	*Primrhavirus gabriel*	San Gabriel mononegavirus (SGMNV)
	*Primrhavirus primus*	Primus virus (PRIMV)
*Stangrhavirus*	*Stangrhavirus elisy*	Elisy virus (ELSYV)
	*Stangrhavirus guadeloupe*	Guadeloupe Culex rhabdovirus (GCRV)
	*Stangrhavirus stang*	Stang virus (STNGV)
	*Stangrhavirus wuhan*	Wuhan mosquito virus 9 (WhMV9)
**Subfamily *Gammarhabdovirinae***		
*Margarhavirus*	*Margarhavirus chemarfal*	chemarfal virus 1 (CHMFV1)
*Novirhabdovirus*	*Novirhabdovirus hirame*	hirame rhabdovirus (HIRRV; HIRV)
	*Novirhabdovirus piscine*	viral haemorrhagic septicemia virus (VHSV)
	*Novirhabdovirus salmonid*	infectious haematopoietic necrosis virus (IHNV)
	*Novirhabdovirus snakehead*	snakehead rhabdovirus (SHRV)
**Unassigned (to subfamilies)**		
*Platrhavirus*	*Platrhavirus microphallus*	microrhabdovirus 1 (MicRV1)
	*Platrhavirus nodulosis*	triaenorhabdovirus 1 (TriRV1)
	*Platrhavirus orientalis*	metorhabdovirus 2 (MetRV2)
	*Platrhavirus pseudoglobulus*	sphaeridiorhabdovirus 1 (SphRV1)
	*Platrhavirus turkestanicum*	schistorhabdovirus 1 (SchRV1)
	*Platrhavirus vulpes*	fox faecal rhabdovirus (FFRV)
**Family *Sunviridae*** [134]		
*Sunshinevirus*	*Sunshinevirus reptilis*	Sunshine Coast virus (SunCV)
**Family *Xinmoviridae*** [135]		
*Alasvirus*	*Alasvirus muscae*	Húběi diptera virus 11 (HbDV11)
*Anphevirus*	*Anphevirus xinchengense*	Xīnchéng mosquito virus (XcMV)
*Corulvirus*	*Corulvirus hangzhouense*	Hángzhōu Zicrona caerulea xinmovirus 1 (HaZCV1)
*Culivirus*	*Culivirus belgradiense*	Serbia mononega-like virus 1 (SerMV1)
	*Culivirus dunyae*	Aedes albopictus anphevirus (AealbAV)
*Doupovirus*	*Doupovirus australiaense*	Culex mononega-like virus 2 (CMLV2)
*Draselvirus*	*Draselvirus dentati*	Húběi rhabdo-like virus 7 (HbRLV7)
*Drunivirus*	*Drunivirus chambonense*	Drosophila unispina virus 1 (DuniV1)
*Gambievirus*	*Gambievirus bolahunense*	Bolahun virus (BLHV)
	*Gambievirus senegalense*	Gambie virus (GAMV)
*Gordisvirus*	*Gordisvirus californiense*	Gordis virus (GorV)
*Gudgevirus*	*Gudgevirus namadgii*	Gudgenby Calliphora mononega-like virus (GuCalMV)
*Gylbovirus*	*Gylbovirus aagae*	Aedes anphevirus (AeAV)
*Hoptevirus*	*Hoptevirus orthopteris*	Húběi orthoptera virus 5 (HbOV5)
*Laumovirus*	*Laumovirus liaoningense*	Fǔshùn Monolepta lauta xinmovirus 2 (FuMLV2)
*Madalivirus*	*Madalivirus amapaense*	Anopheles marajoara virus (AnMV)
	*Madalivirus amazonaense*	Anopheles darlingi virus (AnDV)
*Molauvirus*	*Molauvirus liaoningense*	Fǔshùn Monolepta lauta xinmovirus 1 (FuMLV1)
*Nurnegvirus*	*Nurnegvirus hainanense*	Sānyà Ischnura senegalensis xinmovirus 1 (SISeV1)
*Pelmivirus*	*Pelmivirus eymattense*	hymenopteran anphe-related virus OKIAV71 (HARV71)
*Puclevirus*	*Puclevirus hangzhouense*	Hángzhōu Cletus punctiger xinmovirus 1 (HaCPV1)
*Tecephavirus*	*Tecephavirus sydneyense*	bat faecal-associated anphe-like virus 1 (BtFAV1)
*Triniovirus*	*Triniovirus yonagoense*	Culex tritaeniorhynchus anphevirus (CtAV)
*Trocevirus*	*Trocevirus haikouense*	Bactrocera dorsalis xinmovirus 2 (BDorV)
*Ulegvirus*	*Ulegvirus freckenfeldense*	odonatan anphe-related virus OKIAV59 (OARV59)

Note that viruses are real objects that are assigned to concepts that are called taxa. Species, genera, subfamilies, families and orders are taxa.

* Taxon names are always italicized and always begin with a capital letter.

† Virus names are not italicized and are not capitalized, except if the name or a name component is a proper noun. This column lists the virus names with their correct (lack of) capitalization. Lists of viruses within a given species are provisional at this point and will likely be amended in the near future.

‡ Includes: Newcastle disease virus (NDV).

**Table 5.** Taxonomy of the order *Goujianvirales* (*Negarnaviricota: Haploviricotina: Yunchangviricetes*) as of April 2024

Genus	Species*	Virus (abbreviation)†
**Family *Yueviridae*** [136]		
*Yuyuevirus*	*Yuyuevirus beihaiense*	Běihǎi sesarmid crab virus 3 (BhSCV3)
	*Yuyuevirus shaheense*	Shāhé yuèvirus-like virus 1 (ShYLV1)

Note that viruses are real objects that are assigned to concepts that are called taxa. Species, genera, families and orders are taxa.

* Taxon names are always italicized and always begin with a capital letter.

† Virus names are not italicized and are not capitalized, except if the name or a name component is a proper noun. This column lists the virus names with their correct (lack of) capitalization.

**Table 6.** Taxonomy of the order *Elliovirales* (*Negarnaviricota: Polyploviricotina: Bunyaviricetes*) as of April 2024

Genus	Species*	Virus (abbreviation)†
**Family *Cruliviridae*** [137]		
*Lincruvirus*	*Lincruvirus braziliense*	Callinectes danae portunibunyavirus 1 (CdPBV1)
	*Lincruvirus europense*	European shore crab virus 1 (EscV1)
	*Lincruvirus sinense*	Chinese mitten crab virus 1 (CmcV1)
	*Lincruvirus wenlingense*	Wēnlǐng crustacean virus 9 (WlCV9)
**Family *Fimoviridae*** [138,139]		
*Emaravirus*	*Emaravirus aceris*	maple mottle-associated virus (MaMaV)
	*Emaravirus actinidiae*	Actinidia chlorotic ringspot-associated virus (AcCRaV)
	*Emaravirus ailanthi*	Ailanthus crinkle leaf-associated virus (ACrLaV)
	*Emaravirus artemisiae*	Artemisia fimovirus 1 (ArtV1)
	*Emaravirus cajani*	pigeonpea sterility mosaic virus 1 (PPSMV1)
	*Emaravirus camelliae*	Camellia japonica-associated virus 1 (CjaV1)
	*Emaravirus cercidis*	redbud yellow ringspot-associated virus (RYRaV)
	*Emaravirus chrysanthemi*	chrysanthemum mosaic-associated virus (ChMaV)
	*Emaravirus cordylinae*	ti ringspot-associated virus (TiRSaV)
	*Emaravirus corynocarpi*	karaka Ōkahu purepure emaravirus (KOPV)
	*Emaravirus fici*	fig mosaic virus (FMV)
	*Emaravirus fraxini*	ash shoestring-associated virus (ASaV)
	*Emaravirus idaeobati*	raspberry leaf blotch virus (RLBV)
	*Emaravirus illicii*	Japanese star anise ringspot-associated virus (JSARaV)
	*Emaravirus kiwii*	Actinidia virus 2 (AcV2)
	*Emaravirus kudzu*	Pueraria lobata-associated emaravirus (PloaEV)
	*Emaravirus parkinsoniae*	palo verde broom virus (PVBV)
	*Emaravirus perillae*	Perilla mosaic virus (PerMV)
	*Emaravirus pistaciae*	Pistacia virus B (PiVB)
	*Emaravirus populi*	aspen mosaic-associated virus (AsMaV)
	*Emaravirus pyri*	pear chlorotic leaf spot-associated virus (PCLSaV)
	*Emaravirus quercus*	common oak ringspot-associated virus (CORaV)
	*Emaravirus rosae*	rose rosette virus (RRV)
	*Emaravirus rubi*	blackberry leaf mottle-associated virus (BLMaV)
	*Emaravirus sorbi*	European mountain ash ringspot-associated virus (EMARaV)
	*Emaravirus syringae*	lilac chlorotic ringspot-associated virus (LiCRaV)
	*Emaravirus toordali*	pigeonpea sterility mosaic virus 2 (PPSMV2)
	*Emaravirus tritici*	High Plains wheat mosaic virus (HPWMoV)
	*Emaravirus verbanni*	Camellia japonica-associated virus 2 (CjaV2)
	*Emaravirus visci*	Arceuthobium sichuanense-associated virus 1 (ArSaV1)
	*Emaravirus vitis*	Vitis emaravirus (VEV)
	*Emaravirus ziziphi*	jujube yellow mottle-associated virus (JYMaV)
**Family *Hantaviridae*** [140]		
**Subfamily *Actantavirinae***		
*Actinovirus*	*Actinovirus bernense*	Bern perch virus (BRPV)
	*Actinovirus halieutaeae*	Wēnlǐng minipizza batfish virus (WEMBV)
	*Actinovirus triacanthodis*	Wēnlǐng red spikefish virus (WERSV)
*Percilovirus*	*Percilovirus gobii*	Luposicya lupus actinovirus (LlAV)
	*Percilovirus lophii*	Wēnlǐng yellow goosefish virus (WEYGV)
**Subfamily *Agantavirinae***		
*Agnathovirus*	*Agnathovirus eptatreti*	Wēnlǐng hagfish virus (WEHV)
**Subfamily *Mammantavirinae***		
*Loanvirus*	*Loanvirus brunaense*	Brno virus (BRNV)
	*Loanvirus longquanense*	Lóngquán virus (LQUV)
*Mobatvirus*	*Mobatvirus dakrongense*	Đakrông virus (DKGV)
	*Mobatvirus laibinense*	Láibīn virus (LAIV)
	*Mobatvirus lenaense*	Lena virus (LENV)
	*Mobatvirus novaense*	Nova virus (NVAV)
	*Mobatvirus quezonense*	Quezon virus (QZNV)
	*Mobatvirus robinaense*	Robina virus (ROBV)
	*Mobatvirus xuansonense*	Xuân Sơn virus (XSV)
*Orthohantavirus*	*Orthohantavirus andesense*	Andes virus (ANDV)
	*Orthohantavirus artybashense*	Artybash virus (ARTV)
	*Orthohantavirus asamaense*	Asama virus (ASAV)
	*Orthohantavirus asikkalaense*	Asikkala virus (ASIV)
	*Orthohantavirus bayoui*	bayou virus (BAYV)
		Catacamas virus (CATV)
	*Orthohantavirus boweense*	Bowé virus (BOWV)
	*Orthohantavirus brugesense*	Bruges virus (BRGV)
	*Orthohantavirus caobangense*	Cao Bằng virus (CBNV)
	*Orthohantavirus carrizalense*	Carrizal virus (CARV)
		Huitzilac virus (HUIV)
	*Orthohantavirus chocloense*	Choclo virus (CHOV)
	*Orthohantavirus dabieshanense*	Dàbiéshān virus (DBSV)
	*Orthohantavirus delgaditoense*	Caño Delgadito virus (CADV)
	*Orthohantavirus dobravaense*	Dobrava virus (DOBV)
		Kurkino virus (KURV)
		Saaremaa virus (SAAV)
		Sochi virus (SOCV)
	*Orthohantavirus fugongense*	Fúgòng virus (FUGV)
	*Orthohantavirus hantanense*	Amur virus (AMRV)
		Hantaan virus (HTNV)
		Soochong virus (SOOV)
	*Orthohantavirus jejuense*	Jeju virus (JJUV)
	*Orthohantavirus kenkemeense*	Kenkeme virus (KKMV)
	*Orthohantavirus khabarovskense*	Khabarovsk virus (KHAV)
		Topografov virus (TOPV)
	*Orthohantavirus lankaense*	Lanka virus (LNKV)
	*Orthohantavirus luxiense*	Lúxī virus (LUXV)
	*Orthohantavirus mamorense*	Maripa virus (MARV)
		Rio Mamoré virus (RIOMV)
	*Orthohantavirus maporalense*	Maporal virus (MAPV)
	*Orthohantavirus montanoense*	Montaño virus (MTNV)
	*Orthohantavirus nigrorivense*	Black Creek Canal virus (BCCV)
	*Orthohantavirus prospectense*	Prospect Hill virus (PHV)
	*Orthohantavirus puumalaense*	Hokkaido virus (HOKV)
		Muju virus (MUJV)
		Puumala virus (PUUV)
	*Orthohantavirus rockportense*	Rockport virus (RKPV)
	*Orthohantavirus sangassouense*	Sangassou virus (SANGV)
	*Orthohantavirus seoulense*	Seoul virus (SEOV)
	*Orthohantavirus sinnombreense*	New York virus (NYV)
		Sin Nombre virus (SNV)
	*Orthohantavirus tatenalense*	Tatenale virus (TATV)
	*Orthohantavirus thailandense*	Anjozorobe virus (ANJZV)
		Thailand virus (THAIV)
	*Orthohantavirus tigrayense*	Tigray virus (TIGV)
	*Orthohantavirus tulaense*	Tula virus (TULV)
	*Orthohantavirus wufangense*	Wǔfēng Chodsigoa smithii orthohantavirus 1 (WfCsOHV1)
*Thottimvirus*	*Thottimvirus imjinense*	Imjin virus (MJNV)
	*Thottimvirus thottapalayamense*	Thottapalayam virus (TPMV)
**Subfamily *Repantavirinae***		
*Reptillovirus*	*Reptillovirus hemidactyli*	Hǎinán oriental leaf-toed gecko virus (HOLGV)
**Family *Peribunyaviridae*** [141]		
*Gryffinivirus*	*Gryffinivirus alamedaense*	Udune virus (UDNV)
	*Gryffinivirus hedwigae*	Hedwig virus (HEDV)
*Herbevirus*	*Herbevirus herberti*	Herbert virus (HEBV)
	*Herbevirus kibaleense*	Kibale virus (KIBV)
	*Herbevirus taiense*	Taï virus (TAIV)
*Khurdivirus*	*Khurdivirus volgaense*	Khurdun virus (KURV)
*Lakivirus*	*Lakivirus lakamhaense*	Lakamha virus (LAKV)
*Lambavirus*	*Lambavirus wisconsinense*	largemouth bass bunyavirus (LBBV)
*Orthobunyavirus*	*Orthobunyavirus abrasense*	Abras virus (ABRV)
	*Orthobunyavirus acaraense*	Acará virus (ACAV)
		Benevides virus (BVSV; BENV)
	*Orthobunyavirus achiotei*	Achiote virus (ACHOV)
		trivittatus virus (TVTV)
	*Orthobunyavirus ainoense*	Aino virus (AINOV)
		Kaikalur virus (KAIV)
	*Orthobunyavirus akabaneense*	Akabane virus (AKAV)
		Tinaroo virus (TINV)
	*Orthobunyavirus anadyrense*	Anadyr virus (ANADV)
	*Orthobunyavirus ananindeuaense*	Ananindeua virus (ANUV)
	*Orthobunyavirus angeloense*	San Angelo virus (SAV)
	*Orthobunyavirus anhembiense*	Anhembi virus (AMBV)
	*Orthobunyavirus apeuense*	Apeú virus (APEUV)
	*Orthobunyavirus baakalense*	Baakal virus (BKAV)
	*Orthobunyavirus bakauense*	Bakau virus (BAKV)
		Tanjong Rabok virus (TRV)
	*Orthobunyavirus balagoduense*	Balagodu virus (BLGV)
	*Orthobunyavirus barritaense*	Barrita virus (BITV)
	*Orthobunyavirus bataiense*	Batai virus (BATV)
	*Orthobunyavirus batamaense*	Batama virus (BMAV)
	*Orthobunyavirus belemense*	Belém virus (BLMV)
	*Orthobunyavirus bellavistaense*	Antequera virus (ANTV)
		Barranqueras virus (BQSV)
		Bellavista virus (BELLV)
		Resistencia virus (RTAV)
	*Orthobunyavirus belmontense*	Belmont virus (BELV)
	*Orthobunyavirus benficaense*	Benfica virus (BENV; BNFV)
	*Orthobunyavirus bertiogaense*	Bertioga virus (BERV)
		Cananéia virus (CNAV)
	*Orthobunyavirus biraoense*	Birao virus (BIRV)
	*Orthobunyavirus bobayaense*	Bobaya virus (BOBV)
	*Orthobunyavirus boraceiaense*	Boracéia virus (BORV)
	*Orthobunyavirus botambiense*	Botambi virus (BOTV)
	*Orthobunyavirus bozoense*	Bozo virus (BOZOV)
	*Orthobunyavirus brazoriaense*	brazoran virus (BRAZV)
	*Orthobunyavirus bruconhaense*	Bruconha virus (BRUV)
	*Orthobunyavirus buffaloense*	Buffalo Creek virus (BUCV)
		Murrumbidgee virus (MURBV)
		Trubanaman virus (TRUV)
	*Orthobunyavirus bunyamweraense*	Bunyamwera virus (BUNV)
		Germiston virus (GERV)
		Mboké virus (MBOV)
		Ngari virus (NRIV)
		Stanfield virus (STAV)
		Xingu virus (XINV)
	*Orthobunyavirus bushbushense*	Bushbush virus (BSBV)
		Moriche virus (MORV)
	*Orthobunyavirus buttonwillowense*	Buttonwillow virus (BUTV)
	*Orthobunyavirus bwambaense*	Bwamba virus (BWAV)
		Pongola virus (PGAV)
	*Orthobunyavirus cacheense*	Cache Valley virus (CVV)
		Cholul virus (CHLV)
		Playas virus (PLAV)
		Tlacotalpan virus (TLAV)
	*Orthobunyavirus capimense*	Capim virus (CAPV)
	*Orthobunyavirus caraparuense*	Caraparú virus (CARV)
		El Huayo virus (EHUV)
		Itaquí virus (ITQV)
		Itaya virus (ITYV)
		Ossa virus (OSSAV)
	*Orthobunyavirus catqueense*	Cát Quế virus (CQV)
		Oya virus (OYAV)
	*Orthobunyavirus catuense*	Catú virus (CATUV)
		Moju virus (MOJUV)
	*Orthobunyavirus cuchillaense*	Anopheles B virus (ANBV)
	*Orthobunyavirus ebiense*	Ebinur Lake virus (EBIV)
	*Orthobunyavirus encephalitidis*	California encephalitis virus (CEV)
		Morro Bay virus (MBV)
	*Orthobunyavirus enseadaense*	Enseada virus (ENSV)
	*Orthobunyavirus faceyense*	Facey’s paddock virus (FPV)
	*Orthobunyavirus gamboaense*	Alajuela virus (ALJV)
		Brus Laguna virus (BLAV)
		Calchaquí virus (CQIV)
		Gamboa virus (GAMV)
		Soberanía virus (SOBV)
	*Orthobunyavirus ganganense*	Gan Gan virus (GGV)
		Salt Ash virus (SASHV)
	*Orthobunyavirus guajaraense*	Guajará virus (GJAV)
	*Orthobunyavirus guamaense*	Bimiti virus (BIMV)
		Guamá virus (GMAV)
	*Orthobunyavirus guaratubaense*	Guaratuba virus (GTBV)
		Itimirim virus (ITIV)
	*Orthobunyavirus guaroaense*	Guaroa virus (GROV)
	*Orthobunyavirus gumbolimboense*	Gumbo Limbo virus (GLV)
	*Orthobunyavirus heptayabaense*	Yaba-7 virus (Y7V)
	*Orthobunyavirus horizonteense*	Anopheles A virus (ANAV)
		Arumateua virus (ARTV; ARMTV)
		Caraipé virus (CPEV; CRPV)
		Trombetas virus (TRMV)
		Tucuruí virus (TUCV; TUCRV)
	*Orthobunyavirus iacoense*	Iaco virus (IACOV)
	*Orthobunyavirus ileshaense*	Ilesha virus (ILEV)
	*Orthobunyavirus infirmati*	Infirmatus virus (INFV)
	*Orthobunyavirus ingwavumaense*	Ingwavuma virus (INGV)
	*Orthobunyavirus insulae*	kairi virus (KRIV)
	*Orthobunyavirus jamestownense*	Inkoo virus (INKV)
		Jamestown Canyon virus (JCV)
		Jerry Slough virus (JSV)
		South River virus (SORV)
	*Orthobunyavirus jatobalense*	Jatobal virus (JATV)
	*Orthobunyavirus juandiazense*	Juan Díaz virus (JDV)
	*Orthobunyavirus kaengkhoiense*	Kaeng Khoi virus (KKV)
	*Orthobunyavirus kernense*	Lokern virus (LOKV)
		Main Drain virus (MDV)
		Santa Rosa virus (SARV)
	*Orthobunyavirus ketapangense*	Ketapang virus (KETV)
	*Orthobunyavirus keystoneense*	Keystone virus (KEYV)
	*Orthobunyavirus khatangaense*	Khatanga virus (KHATV)
		snowshoe hare virus (SSHV)
	*Orthobunyavirus koongoli*	koongol virus (KOOV)
		wongal virus (WONV)
	*Orthobunyavirus kowanyamaense*	Kowanyama virus (KOWV)
	*Orthobunyavirus lacrosseense*	La Crosse virus (LACV)
	*Orthobunyavirus lasmaloyasense*	Las Maloyas virus (LMV)
	*Orthobunyavirus leanyerense*	Leanyer virus (LEAV)
	*Orthobunyavirus ledniceense*	Lednice virus (LEDV)
	*Orthobunyavirus lukuniense*	Lukuni virus (LUKV)
	*Orthobunyavirus lumboense*	Lumbo virus (LUMV)
	*Orthobunyavirus macauaense*	Macauã virus (MCAV)
	*Orthobunyavirus madridense*	Madrid virus (MADV)
		Vinces virus (VINV)
	*Orthobunyavirus maguariense*	Maguari virus (MAGV)
	*Orthobunyavirus mahoganyense*	Mahogany Hammock virus (MHV)
	*Orthobunyavirus manzanillaense*	Manzanilla virus (MANV)
		Inini virus (INIV)
	*Orthobunyavirus maprikense*	Maprik virus (MPKV)
	*Orthobunyavirus maritubaense*	Marituba virus (MTBV)
		Zungarococha virus (ZUNV)
	*Orthobunyavirus matruhense*	Matruh virus (MTRV)
	*Orthobunyavirus melajoense*	Melao virus (MELV)
	*Orthobunyavirus mermetense*	Mermet virus (MERV)
	*Orthobunyavirus minatitlanense*	Minatitlán virus (MNTV)
		Palestina virus (PLSV)
	*Orthobunyavirus mirimense*	Mirim virus (MIRV)
	*Orthobunyavirus mitchellense*	Mapputta virus (MAPV)
	*Orthobunyavirus mpokoense*	M’Poko virus (MPOV)
		Yaba-1 virus (Y1V)
	*Orthobunyavirus navioense*	Serra do Navio virus (SDNV)
	*Orthobunyavirus nepuyoi*	Nepuyo virus (NEPV)
	*Orthobunyavirus nesszionaense*	Ness Ziona virus (NZV)
	*Orthobunyavirus nolaense*	Nola virus (NOLAV)
	*Orthobunyavirus northwayense*	Northway virus (NORV)
	*Orthobunyavirus nyandoense*	Eretmapodites virus (ERETV)
		Mojuí dos Campos virus (MDCV)
		Nyando virus (NDV)
	*Orthobunyavirus okolaense*	Okola virus (OKOV)
	*Orthobunyavirus olifantsvleiense*	Bobia virus (BIAV)
		Dabakala virus (DABV)
		Olifantsvlei virus (OLIV)
		Oubi virus (OUBIV)
	*Orthobunyavirus oribocaense*	Murutucú virus (MURV)
		Oriboca virus (ORIV)
		Restan virus (RESV)
	*Orthobunyavirus oropoucheense*	Iquitos virus (IQTV)
		Madre de Dios virus (MDDV)
		Oropouche virus (OROV)
		Perdões virus (PDEV)
		Pintupo virus (PINTV)
	*Orthobunyavirus oyoense*	Oyo virus (OYOV)
	*Orthobunyavirus pacoraense*	Pacora virus (PCAV)
	*Orthobunyavirus parkeri*	Parker’s Farm virus (PFV)
	*Orthobunyavirus patoisense*	Babahoya virus (BABV)
		Patois virus (PATV)
	*Orthobunyavirus peachesterense*	peaton virus (PEAV)
	*Orthobunyavirus porteiraense*	Cachoeira Porteira virus (CPOV)
	*Orthobunyavirus potosiense*	Potosi virus (POTV)
	*Orthobunyavirus saboense*	Sabo virus (SABOV)
	*Orthobunyavirus sangoense*	Sango virus (SANV)
	*Orthobunyavirus sanjuanense*	Pueblo Viejo virus (PVV)
		San Juan virus (SJV)
	*Orthobunyavirus schmallenbergense*	Douglas virus (DOUV)
		Sathuperi virus (SATV)
		Schmallenberg virus (SBV)
		Shamonda virus (SHAV)
	*Orthobunyavirus sedlecense*	Sedlec virus (SEDV)
	*Orthobunyavirus shermanense*	Fort Sherman virus (FSV)
		Laguna Larga virus (LLV)
	*Orthobunyavirus shokweense*	Shokwe virus (SHOV)
	*Orthobunyavirus shuniense*	Shuni virus (SHUV)
	*Orthobunyavirus simbuense*	Para virus (PARAV)
		Simbu virus (SIMV)
	*Orthobunyavirus sororocaense*	Sororoca virus (SORV)
	*Orthobunyavirus squalofluvii*	Shark River virus (SRV)
	*Orthobunyavirus sussexense*	Little Sussex virus (LTLSV)
	*Orthobunyavirus tacaiumaense*	CoAr 1071 virus (CA1071V)
		CoAr 3627 virus (CA3626V)
		Tacaiuma virus (TCMV)
		Virgin River virus (VRV)
	*Orthobunyavirus tahynaense*	Ťahyňa virus (TAHV)
	*Orthobunyavirus tangaense*	Tanga virus (TANV)
	*Orthobunyavirus tataguineense*	Tataguine virus (TATV)
	*Orthobunyavirus telokense*	Telok Forest virus (TFV)
	*Orthobunyavirus tensawense*	Tensaw virus (TENV)
	*Orthobunyavirus termeilense*	Termeil virus (TERV)
	*Orthobunyavirus teteense*	Bahig virus (BAHV)
		Tete virus (TETEV)
		Tsuruse virus (TSUV)
		Weldona virus (WELV)
	*Orthobunyavirus thimiriense*	Thimiri virus (THIV)
	*Orthobunyavirus timboteuaense*	Timboteua virus (TBTV)
	*Orthobunyavirus trinitiense*	Triniti virus (TNTV)
	*Orthobunyavirus turlockense*	Turlock virus (TURV)
	*Orthobunyavirus umbreense*	Umbre virus (UMBV)
	*Orthobunyavirus utingaense*	Utinga virus (UTIV)
	*Orthobunyavirus witwatersrandense*	Witwatersrand virus (WITV)
	*Orthobunyavirus wolkbergense*	Wolkberg virus (WBV)
	*Orthobunyavirus wyeomyiae*	Rio Pracupi virus
		Taiassui virus (TAIAV)
		Tucunduba virus (TUCV)
		Wyeomyia virus (WYOV)
	*Orthobunyavirus yacaabaense*	Yacaaba virus (YACV)
*Pacuvirus*	*Pacuvirus caimitoense*	Caimito virus (CAIV)
	*Pacuvirus chilibreense*	Chilibre virus (CHIV)
	*Pacuvirus evaense*	Rio Preto da Eva virus (RPEV)
	*Pacuvirus pacuiense*	Pacui virus (PACV)
	*Pacuvirus tapirapeense*	Tapirapé virus (TAPV)
*Shangavirus*	*Shangavirus shuangaoense*	Shuāngào insect virus 1 (SgIV1)
**Family *Phasmaviridae*** [142]		
*Cicadellivirus*	*Cicadellivirus scaphoidei*	Scaphoideus titanus bunya-like virus 1 (StHV1)
*Feravirus*	*Feravirus ferakinum*	Ferak virus (FRKV)
	*Feravirus guaguaense*	Guagua virus (GUAV)
	*Feravirus hemipterus*	hemipteran phasma-related virus OKIAV247 (HeFV)
	*Feravirus neuropterus*	neuropteran phasma-related virus OKIAV248 (NeFV)
*Hymovirus*	*Hymovirus chrysis*	hymenopteran phasma-related virus OKIAV250 (HyHV2)
	*Hymovirus chrysurae*	hymenopteran phasma-related virus OKIAV252 (HyHV1)
*Jonvirus*	*Jonvirus eboris*	jonchet virus (JONV)
*Orthophasmavirus*	*Orthophasmavirus aedis*	Wǔhàn mosquito virus 2 (WhMV2)
	*Orthophasmavirus anophelae*	Anopheles triannulatus orthophasmavirus (AtOPV)
	*Orthophasmavirus barstukasense*	barstukas virus (BARV)
	*Orthophasmavirus chrysis*	hymenopteran phasma-related virus OKIAV227 (HyOV2)
	*Orthophasmavirus coleopterus*	coleopteran phasma-related virus OKIAV235 (CPRV)
	*Orthophasmavirus coredoense*	Coredo virus (CORV)
	*Orthophasmavirus culicis*	Culex phasma-like virus (CPLV)
	*Orthophasmavirus flenense*	Flen virus (FLNV)
	*Orthophasmavirus fushunense*	Fǔshùn phasmavirus 2 (FsnPV2)
	*Orthophasmavirus gandaense*	Ganda bee virus (GBEEV)
	*Orthophasmavirus hubeiense*	Húběi odonate virus 9 (HbOV9)
	*Orthophasmavirus kigluaikense*	Kigluaik phantom virus (KIGV)
	*Orthophasmavirus miglotasense*	miglotas virus (MIGV)
	*Orthophasmavirus niuklukense*	Niukluk phantom virus (NUKV)
	*Orthophasmavirus odonatus*	Húběi odonate virus 8 (HbOV8)
	*Orthophasmavirus philoctetis*	hymenopteran phasma-related virus OKIAV228 (HyOV1)
	*Orthophasmavirus sogatellae*	Fǔshùn phasmavirus 1 (FsnPV1)
	*Orthophasmavirus wuchangense*	Wǔchāng cockroach virus 1 (WcCV1)
	*Orthophasmavirus wuhanense*	Wǔhàn mosquito virus 1 (WhMV1)
*Sawastrivirus*	*Sawastrivirus sanxiaense*	Sānxiá water strider virus 2 (SxWSV2)
*Wuhivirus*	*Wuhivirus insecti*	Wǔhàn insect virus 2 (WhIV2)
**Family *Tospoviridae***		
*Orthotospovirus*	*Orthotospovirus alstroemeriflavi*	Alstroemeria yellow spot virus (AYSV)
	*Orthotospovirus alstroemerinecrosis*	Alstroemeria necrotic streak virus (ANSV)
	*Orthotospovirus arachianuli*	groundnut ringspot virus (GRSV)
	*Orthotospovirus arachiflavamaculae*	groundnut yellow spot virus (GYSV)
	*Orthotospovirus arachiflavi*	groundnut chlorotic fan-spot virus (GCFSV)
	*Orthotospovirus arachinecrosis*	groundnut bud necrosis virus (GBNV)
	*Orthotospovirus callaflavi*	calla lily chlorotic spot virus (CCSV)
	*Orthotospovirus capsiciflavi*	Capsicum chlorosis virus (CaCV)
	*Orthotospovirus capsicimaculaflavi*	pepper chlorotic spot virus (PCSV)
	*Orthotospovirus chrysanthinecrocaulis*	Chrysanthemum stem necrosis virus (CSNV)
	*Orthotospovirus citrullomaculosi*	watermelon silver mottle virus (WSMoV)
	*Orthotospovirus citrullonecrosis*	watermelon bud necrosis virus (WBNV)
	*Orthotospovirus cucurbichlorosis*	zucchini lethal chlorosis virus (ZLCV)
	*Orthotospovirus glycininecrovenae*	soybean vein necrosis virus (SVNV)
	*Orthotospovirus hippeflavi*	Hippeastrum chlorotic spot virus (HCRV)
	*Orthotospovirus impatiensnecromaculae*	impatiens necrotic spot virus (INSV)
	*Orthotospovirus iridimaculaflavi*	iris yellow spot virus (IYSV)
	*Orthotospovirus meloflavi*	melon yellow spot virus (MYSV)
	*Orthotospovirus melotessellati*	melon severe mosaic virus (MSMV)
	*Orthotospovirus morivenae*	mulberry vein banding-associated virus (MVBaV)
	*Orthotospovirus phaseolinecrotessellati*	bean necrotic mosaic virus (BeNMV)
	*Orthotospovirus polygonianuli*	Polygonum ringspot virus (PolRSV)
	*Orthotospovirus tomatanuli*	tomato yellow ring virus (TYRV)
	*Orthotospovirus tomatoflavi*	tomato chlorotic spot virus (TCSV)
	*Orthotospovirus tomatomaculae*	tomato spotted wilt virus (TSWV)
	*Orthotospovirus tomatozonae*	tomato zonate spot virus (TZSV)
**Family *Tulasviridae*** [143]		
*Orthotulasvirus*	*Orthotulasvirus tulasnellae*	Tulasnella bunyavirales-like virus 1 (TB-LV)
*Paratulasvirus*	*Paratulasvirus agrocybes*	Agrocybe praecox tulasvirus 1 (ApTV1)

Note that viruses are real objects that are assigned to concepts that are called taxa. Species, genera, subfamilies, families and orders are taxa.

* Taxon names are always italicized and always begin with a capital letter.

† Virus names are not italicized and are not capitalized, except if the name or a name component is a proper noun. This column lists the virus names with their correct (lack of) capitalization. Lists of viruses within a given species are provisional at this point and will likely be amended in the near future.

**Table 7.** Taxonomy of the order *Hareavirales* (*Negarnaviricota: Polyploviricotina: Bunyaviricetes*) as of April 2024

Genus	Species*	Virus (abbreviation)†
**Family *Arenaviridae*** [144]		
*Antennavirus*	*Antennavirus hirsutum*	Wēnlǐng frogfish arenavirus 2 (WlFAV2)
	*Antennavirus salmonis*	salmon pescarenavirus 1 (SPAV1)
		salmon pescarenavirus 2 (SPAV2)
	*Antennavirus striale*	Wēnlǐng frogfish arenavirus 1 (WlFAV1)
*Hartmanivirus*	*Hartmanivirus brazilense*	SetPatVet virus 1 (SPVV1)
	*Hartmanivirus haartmani*	Haartman Institute snake virus 1 (HISV1)
		Haartman Institute snake virus 2 (HISV2)
	*Hartmanivirus helvetiae*	Dante Muikkunen virus 1 (DaMV1)
	*Hartmanivirus patriae*	andere Heimat virus 1 (aHeV1)
	*Hartmanivirus quadrati*	big electron-dense squares virus 1 (BESV1)
	*Hartmanivirus scholae*	old schoolhouse virus 1 (OScV1)
		old schoolhouse virus 2 (OScV2)
	*Hartmanivirus turici*	veterinary pathology Zurich virus 1 (VPZV1)
		veterinary pathology Zurich virus 2 (VPZV2)
	*Hartmanivirus unni*	Universidad Nacional virus 1 (UnNV1)
*Innmovirus*	*Innmovirus hailarense*	Hailar virus (HLRV)
*Mammarenavirus*	*Mammarenavirus abaense*	Ābà-Miányáng virus (AMYV)
	*Mammarenavirus alashanense*	Alxa virus (ALXV)
	*Mammarenavirus allpahuayoense*	Allpahuayo virus (ALLV)
	*Mammarenavirus amapariense*	Amaparí virus (AMAV)
	*Mammarenavirus aporeense*	Aporé virus (APOV)
	*Mammarenavirus batangense*	Bātáng virus (BTTV)
	*Mammarenavirus bearense*	Bear Canyon virus (BCNV)
	*Mammarenavirus beregaense*	Berega virus (BEGV)
	*Mammarenavirus bituense*	Bitu virus (BITV)
	*Mammarenavirus brazilense*	Sabiá virus (SBAV)
	*Mammarenavirus caliense*	Pichindé virus (PICHV)
	*Mammarenavirus cameroonense*	souris virus (SOUV)
	*Mammarenavirus chapareense*	Chapare virus (CHAPV)
	*Mammarenavirus choriomeningitidis*	Dandenong virus (DANV)
		lymphocytic choriomeningitis virus (LCMV)
	*Mammarenavirus cupixiense*	Cupixi virus (CUPXV)
	*Mammarenavirus dhati-welelense*	Dhati Welel virus (DHWV)
	*Mammarenavirus flexalense*	Flexal virus (FLEV)
	*Mammarenavirus gairoense*	Gairo virus (GAIV)
	*Mammarenavirus ganziense*	Gānzī virus (GNZV)
	*Mammarenavirus guanaritoense*	Guanarito virus (GTOV)
	*Mammarenavirus ippyense*	Ippy virus (IPPYV)
	*Mammarenavirus juninense*	Junín virus (JUNV)
	*Mammarenavirus kitaleense*	Kitale virus (KTLV)
	*Mammarenavirus kwanzaense*	Kwanza virus (KWAV)
	*Mammarenavirus lassaense*	Lassa virus (LASV)
	*Mammarenavirus latinum*	Latino virus (LATV)
	*Mammarenavirus lijiangense*	Lìjiāng virus (LIJV)
	*Mammarenavirus loeiense*	Loei River virus (LORV)
	*Mammarenavirus lujoense*	Lujo virus (LUJV)
	*Mammarenavirus lunaense*	Luli virus (LULV)
		Luna virus (LUAV)
	*Mammarenavirus lunkense*	Lunk virus (LNKV)
	*Mammarenavirus machupoense*	Machupo virus (MACV)
	*Mammarenavirus mafigaense*	Mafiga virus (MAFV)
	*Mammarenavirus marientalense*	Mariental virus (MRLV)
	*Mammarenavirus mecsekense*	Mecsek Mountains virus (MEMV)
	*Mammarenavirus merinoense*	Merino Walk virus (MRWV)
	*Mammarenavirus mopeiaense*	Mopeia virus (MOPV)
		Morogoro virus (MORV)
	*Mammarenavirus ngerengerense*	Ngerengere virus (NGEV)
	*Mammarenavirus okahandjaense*	Okahandja virus (OKAV)
	*Mammarenavirus oliverosense*	Oliveros virus (OLVV)
	*Mammarenavirus paranaense*	Paraná virus (PRAV)
	*Mammarenavirus piritalense*	Pirital virus (PIRV)
	*Mammarenavirus praomyidis*	mobala virus (MOBV)
	*Mammarenavirus ryukyuense*	Ryukyu virus (RYKV)
	*Mammarenavirus solweziense*	Solwezi virus (SOLV)
	*Mammarenavirus songeaense*	Songea virus (SOGV)
	*Mammarenavirus tacaribeense*	Tacaribe virus (TCRV)
	*Mammarenavirus tamiamiense*	Tamiami virus (TMMV)
	*Mammarenavirus tietense*	Tietê virus (TIEV)
	*Mammarenavirus wenzhouense*	Wēnzhōu virus (WENV)
	*Mammarenavirus whitewaterense*	Big Brushy Tank virus (BBRTV)
		Catarina virus (CTNV)
		Skinner Tank virus (SKTV)
		Tonto Creek virus (TTCV)
		Whitewater Arroyo virus (WWAV)
	*Mammarenavirus xapuriense*	Xapuri virus (XAPV)
*Reptarenavirus*	*Reptarenavirus aurei*	Golden Gate virus (GOGV)
	*Reptarenavirus californiae*	CAS virus (CASV)
	*Reptarenavirus commune*	tavallinen suomalainen mies virus 2 (TSMV2)
	*Reptarenavirus giessenae*	University of Giessen virus 1 (UGV1)
		University of Giessen virus 2 (UGV2)
		University of Giessen virus 3 (UGV3)
	*Reptarenavirus rotterdamense*	ROUT virus (ROUTV)
		University of Helsinki virus 1 (UHV1)
**Family *Discoviridae*** [145]		
*Orthodiscovirus*	*Orthodiscovirus coniellae*	Coniothyrium diplodiella negative-stranded RNA virus 1 (CdNSRV1)
		Plasmopara viticola lesion-associated mycobunyavirales-like virus 9 (PvLAMLV9)
	*Orthodiscovirus hispaniae*	Plasmopara viticola lesion-associated mycobunyavirales-like virus 8 (PvLAMLV8)
	*Orthodiscovirus iberiae*	Plasmopara viticola lesion-associated mycobunyavirales-like virus 4 (PvLAMLV4)
	*Orthodiscovirus missouriense*	Penicillium discovirus (PDV)
	*Orthodiscovirus oryzae*	rice dwarf-associated bunya-like virus (RDaBV)
	*Orthodiscovirus penicillii*	Penicillium roseopurpureum-negative ssRNA virus 1 (PrNssV1)
**Family *Konkoviridae***		
*Olpivirus*	*Olpivirus tulipae*	tulip streak virus (TuSV)
**Family *Leishbuviridae*** [146]		
*Shilevirus*	*Shilevirus leptomonadis*	Leptomonas moramango virus (LEPMV)
**Family *Mypoviridae*** [147]		
*Hubavirus*	*Hubavirus myriapedis*	Húběi myriapoda virus 5 (HbMV5)
**Family *Nairoviridae*** [148]		
*Norwavirus*	*Norwavirus beijiense*	Běijí nairovirus (BJNV)
	*Norwavirus grotenhoutense*	Grotenhout virus (GRHV)
*Ocetevirus*	*Ocetevirus paratemnopterygis*	red goblin roach virus 1 (RGRV1)
*Orthonairovirus*	*Orthonairovirus abuhammadense*	Abū Ḥammād virus (AHV)
	*Orthonairovirus abuminaense*	Abū Mīnā virus (AMV)
	*Orthonairovirus amblyommae*	kupe virus (KUPEV)
	*Orthonairovirus antuense*	Āntú virus (ANTV)
	*Orthonairovirus artashatense*	Artashat virus (ARTSV)
	*Orthonairovirus australiaense*	Vinegar Hill virus (VINHV)
	*Orthonairovirus avalonense*	Avalon virus (AVAV)
	*Orthonairovirus bandiaense*	Bandia virus (BDAV)
	*Orthonairovirus buranaense*	Burana virus (BURV)
	*Orthonairovirus bushkeyense*	Caspiy virus (CASV)
		Farallon virus (FARV)
		Great Saltee virus (GRSV)
		Hughes virus (HUGV)
		Raza virus (RAZAV)
	*Orthonairovirus chimense*	Chim virus (CHIMV)
	*Orthonairovirus clomorense*	Clo Mor virus (CMV; CLMV)
	*Orthonairovirus crocidurae*	Wǔfēng Crocidura attenuata orthonairovirus 1 (WfCAV1)
	*Orthonairovirus dermacentoris*	Pacific Coast tick nairovirus (PCTNV)
	*Orthonairovirus dugbeense*	Dugbe virus (DUGV)
	*Orthonairovirus erveense*	Erve virus (ERVEV)
	*Orthonairovirus esteroense*	Estero Real virus (ERV)
	*Orthonairovirus gossasense*	Gossas virus (GOSV)
	*Orthonairovirus gubboense*	Gubbo nairovirus (GUBV)
	*Orthonairovirus haemorrhagiae*	Crimean-Congo haemorrhagic fever virus (CCHFV)
	*Orthonairovirus hazaraense*	Hazara virus (HAZV)
	*Orthonairovirus huangpiense*	Huángpí tick virus 1 (HpTV1)
	*Orthonairovirus issykkulense*	Issyk-kul virus (ISKV)
	*Orthonairovirus japonicum*	tofla virus (TFLV)
	*Orthonairovirus kasokeroense*	Kasokero virus (KASV; KASOV)
	*Orthonairovirus keterehense*	Keterah virus (KTRV)
		Uzun-Agach virus (UZAV)
	*Orthonairovirus khani*	Dera Ghazi Khan virus (DGKV)
	*Orthonairovirus lambarenense*	lamusara virus (LMSV)
	*Orthonairovirus lusakaense*	Leopards Hill virus (LPHV)
	*Orthonairovirus macquariense*	Taggert virus (TAGV)
	*Orthonairovirus manidae*	pangolin orthonairovirus (PONV)
	*Orthonairovirus meihuashanense*	Méihuā Mountain virus (MHMV)
	*Orthonairovirus meramense*	Meram virus (MEMV)
	*Orthonairovirus nairobiense*	Nairobi sheep disease virus (NSDV)
	*Orthonairovirus parahaemorrhagiae*	Aigai virus (AIGV)
	*Orthonairovirus peruense*	Punta Salinas virus (PSV)
	*Orthonairovirus qalyubense*	Geran virus (GERV)
		Qalyub virus (QYBV)
	*Orthonairovirus randallense*	Sapphire II virus (SAPV)
	*Orthonairovirus sakhalinense*	Sakhalin virus (SAKV)
		Tillamook virus (TILLV)
	*Orthonairovirus sinense*	wawsin virus (WAWV)
	*Orthonairovirus soldadoense*	Soldado virus (SOLV)
	*Orthonairovirus songlingense*	Sōnglǐng virus (SGLV)
	*Orthonairovirus sulinaense*	Sulina virus (SULV)
	*Orthonairovirus sunci*	cencurut virus (CENV)
	*Orthonairovirus tachengense*	Tǎchéng tick virus 1 (TcTV1)
	*Orthonairovirus thiaforaense*	Thiafora virus (TFAV)
	*Orthonairovirus tomdiense*	Tamdy virus (TAMV)
	*Orthonairovirus tunisense*	Tunis virus (TUNV)
	*Orthonairovirus wenzhouense*	Wēnzhōu tick virus (WzTV)
	*Orthonairovirus yezoense*	Yezo virus (YEZV)
	*Orthonairovirus yogueense*	Yogue virus (YOGV)
	*Orthonairovirus zirkuense*	Zirqa virus (ZIRV)
*Sabavirus*	*Sabavirus americanum*	South Bay virus (SBV)
*Shaspivirus*	*Shaspivirus aranei*	Shāyáng spider virus 1 (SySV1)
*Sitinavirus*	*Sitinavirus sichuanense*	Sìchuān tick nairovirus (ScTNV)
*Striwavirus*	*Striwavirus sanxiaense*	Sānxiá water strider virus 1 (SxWSV1)
*Xinspivirus*	*Xinspivirus xinzhouense*	Xīnzhōu spider virus (XSV)
**Family *Phenuiviridae*** [149]		
*Bandavirus*	*Bandavirus albatrossense*	Albatross Island virus (ABIV)
	*Bandavirus amblyommae*	lone star virus (LSV)
	*Bandavirus bhanjanagarense*	Bhanja virus (BHAV)
	*Bandavirus dabieense*	severe fever with thrombocytopenia syndrome virus (SFTSV)
	*Bandavirus guertuense*	Gùěrtú virus (GTV)
	*Bandavirus heartlandense*	Heartland virus (HRTV)
	*Bandavirus kismaayoense*	Kismaayo virus (KISV)
	*Bandavirus razdanense*	Razdan virus (RAZV)
	*Bandavirus zwieselense*	Zwiesel bat bandavirus (ZbbV)
*Beidivirus*	*Beidivirus muscae*	Húběi diptera virus 3 (HbDV3)
*Bocivirus*	*Bocivirus botrytidis*	Botrytis cinerea bocivirus 1 (BcBV1)
	*Bocivirus fusarii*	Fusarium sibiricum coguvirus 1 (FsCV1)
	*Bocivirus trichodermae*	Trichoderma gamsii cogu-like virus 1 (TgClV1)
	*Bocivirus viticulum*	grapevine-associated cogu-like virus 1 (GaCLV1)
*Citricivirus*	*Citricivirus chongqinense*	Aphis citricidus bunyavirus (AcBV)
*Coguvirus*	*Coguvirus chinense*	Brassica campestris chinensis coguvirus 1 (BCCoV1)
	*Coguvirus chrysanthae*	Edgeworthia chrysantha mosaic-associated virus (ECMaV)
	*Coguvirus citri*	citrus concave gum-associated virus (CCGaV)
	*Coguvirus citrulli*	watermelon crinkle leaf-associated virus 1 (WCLaV1)
	*Coguvirus eburi*	citrus virus A (CiVA)
	*Coguvirus henanense*	watermelon crinkle leaf-associated virus 2 (WCLaV2)
	*Coguvirus yunnanense*	Yúnnán Paris negative-stranded virus (YPNSV)
*Entovirus*	*Entovirus entoleucae*	Entoleuca phenui-like virus 1 (EnPLV1)
*Goukovirus*	*Goukovirus aphalarae*	Aphalara polygoni bunya-like virus (ApBLV)
	*Goukovirus ceraphri*	Ceraphron bunya-like virus (CerBLV)
	*Goukovirus cumutoense*	Cumuto virus (CUMV)
	*Goukovirus gouleakoense*	Gouléako virus (GOLV)
	*Goukovirus yichangense*	Yíchāng insect virus (YcIV)
*Horwuvirus*	*Horwuvirus fitzroyense*	Fitzroy Crossing tenui-like virus 1 (FCTenV1)
	*Horwuvirus solenopsidis*	Solenopsis invicta virus 14 (SINV14)
	*Horwuvirus wuhanense*	Wǔhàn horsefly virus (WhHV)
*Hudivirus*	*Hudivirus muscae*	Húběi diptera virus 4 (HbDV4)
*Hudovirus*	*Hudovirus lepidopteris*	Húběi lepidoptera virus 1 (HbLV1)
*Ixovirus*	*Ixovirus heckscherense*	blacklegged tick virus 1 (BLTV1)
	*Ixovirus ixodis*	blacklegged tick virus 3 (BLTV3)
	*Ixovirus norvegiae*	Fairhair virus (FHAV)
*Laulavirus*	*Laulavirus alphaviticulum*	grapevine-associated cogu-like virus 2 (GaCLV2)
	*Laulavirus betaviticulum*	grapevine-associated cogu-like virus 3 (GaCLV3)
	*Laulavirus gammaviticulum*	grapevine-associated cogu-like virus 4 (GaCLV4)
	*Laulavirus laurelense*	Laurel Lake virus (LLV)
	*Laulavirus wardellense*	Wardell virus (WRDV)
*Lentinuvirus*	*Lentinuvirus lentinulae*	Lentinula edodes negative-strand RNA virus 2 (LeNSRV2)
*Mechlorovirus*	*Mechlorovirus cucumeris*	melon chlorotic spot virus (MeCSV)
	*Mechlorovirus ramuense*	Ramu stunt virus (RmSV)
*Mobuvirus*	*Mobuvirus arnae*	Euproctis pseudoconspersa bunyavirus (EPBV)
	*Mobuvirus mothrae*	Mothra virus (MTHV)
	*Mobuvirus narangueense*	Ñarangue virus (NRGV)
	*Mobuvirus stephanocirci*	Browner virus (BRWV)
*Phasivirus*	*Phasivirus baduense*	Badu virus (BADUV)
	*Phasivirus guadeloupeense*	Guadeloupe mosquito phasivirus (GMPV)
	*Phasivirus hubeiense*	Húběi diptera virus 5 (HbDV5)
	*Phasivirus parryense*	Parry’s Creek phasivirus 1 (PCPhasV1)
	*Phasivirus phasiense*	Phasi Charoen-like virus (PCLV)
	*Phasivirus wuhanense*	Wǔhàn fly virus 1 (WhFV1)
	*Phasivirus wutaiense*	Wǔtái mosquito virus (WtMV)
*Phlebovirus*	*Phlebovirus adanaense*	Adana virus (ADAV)
	*Phlebovirus aguacateense*	Aguacate virus (AGUV)
	*Phlebovirus alcubeense*	Alcube virus (ACBV)
	*Phlebovirus alenquerense*	Alenquer virus (ALEV)
	*Phlebovirus almendrasense*	Tres Almendras virus (TRAV)
	*Phlebovirus ambeense*	Ambé virus (ABEV)
	*Phlebovirus anhangaense*	Anhangá virus (ANHV)
	*Phlebovirus arumowotense*	Arumowot virus (AMTV)
	*Phlebovirus bogoriaense*	Bogoria virus (BGRV)
	*Phlebovirus buenaventuraense*	Buenaventura virus (BUEV)
	*Phlebovirus bujaruense*	Bujaru virus (BUJV)
	*Phlebovirus cacaoense*	Cacao virus (CACV)
	*Phlebovirus campanaense*	Campana virus (CMAV)
	*Phlebovirus candiruense*	Ariquemes virus (ARQV)
		Candirú virus (CDUV)
		Jacundá virus (JCNV)
		Morumbi virus (MRBV; MRMBV)
		Mucura virus (MCRV; MRAV)
		Serra Norte virus (SRNV)
	*Phlebovirus chagresense*	Chagres virus (CHGV)
	*Phlebovirus claroense*	Rio Claro virus (RICV)
	*Phlebovirus cocleense*	Coclé virus (CCLV)
	*Phlebovirus corfouense*	Corfou virus (CFUV)
	*Phlebovirus dashliense*	Dāshlī virus (DASV)
	*Phlebovirus duraniaense*	Durania virus (DRNV)
	*Phlebovirus echarateense*	Echarate virus (ECHV)
	*Phlebovirus embossosense*	Embossos virus (EMBV)
	*Phlebovirus florisense*	Saint-Floris virus (SAFV)
	*Phlebovirus gabekense*	Gabek forest virus (GFV)
	*Phlebovirus gloriaense*	La Gloria virus (LAGV)
	*Phlebovirus gordilense*	Gordil virus (GORV)
	*Phlebovirus hediense*	Hédǐ virus (HEDV)
	*Phlebovirus icoaraciense*	Icoaraci virus (ICOV)
	*Phlebovirus itaitubaense*	Itaituba virus (ITAV)
	*Phlebovirus itaporangaense*	Itaporanga virus (ITPV)
	*Phlebovirus ixcanalense*	Ixcanal virus (IXCV)
	*Phlebovirus karimabadense*	Karimabad virus (KARV)
	*Phlebovirus kiborgochense*	Kiborgoch virus (KBGV)
	*Phlebovirus leticiaense*	Leticia virus (LTCV)
	*Phlebovirus limboense*	frijoles virus (FRIV)
		Joá virus (JOAV)
	*Phlebovirus maldonadoense*	Maldonado virus (MLOV)
	*Phlebovirus massiliaense*	Massilia virus (MASV)
	*Phlebovirus medjerdaense*	Medjerda Valley virus (MVV)
	*Phlebovirus monagritaense*	Mona Grita virus (MOGV)
	*Phlebovirus mukawaense*	Mukawa virus (MKWV)
	*Phlebovirus mungubaense*	Munguba virus (MUNV)
	*Phlebovirus napoliense*	Arrábida virus (ARRV)
		Balkan virus (BALKV)
		Fermo virus (FERV)
		Granada virus (GRV; GRAV)
		Saddaguia virus (SADV)
		sandfly fever Naples virus (SFNV)
	*Phlebovirus niqueense*	Nique virus (NIQV)
	*Phlebovirus ntepesense*	Ntepes virus (NTPV)
	*Phlebovirus odrenisrouense*	Odrénisrou virus (ODRV)
	*Phlebovirus oriximinaense*	Oriximiná virus (ORXV)
	*Phlebovirus pantanalense*	viola virus (VIOV)
	*Phlebovirus penablancaense*	Peña Blanca virus (PEBV)
	*Phlebovirus penshurtense*	Penshurt virus (PEHV)
	*Phlebovirus perkerraense*	Perkerra virus (PKEV)
	*Phlebovirus puniqueense*	Punique virus (PUNV)
	*Phlebovirus riftense*	Rift Valley fever virus (RVFV)
	*Phlebovirus riograndense*	Rio Grande virus (RGV)
	*Phlebovirus salangaense*	Salanga virus (SLGV)
	*Phlebovirus salehabadense*	Adria virus (ADRV)
		Arbia virus (ARBV)
		Bregalaka virus (BREV)
		Olbia virus (OLBV)
		Salehabad virus (SALV)
		Zaba virus (ZABAV)
	*Phlebovirus saloboense*	Salobo virus (SLBOV)
	*Phlebovirus siciliaense*	sandfly fever Sicilian virus (SFSV)
	*Phlebovirus taparaense*	Tapará virus (TPRV)
	*Phlebovirus tehranense*	Tehran virus (THEV)
	*Phlebovirus ticoense*	Tico virus (TICV)
	*Phlebovirus toroense*	Capira virus (CAPIV)
		Punta Toro virus (PTV)
	*Phlebovirus torosense*	Toros virus (TORV)
	*Phlebovirus toscanaense*	Toscana virus (TOSV)
	*Phlebovirus turunaense*	Turuna virus (TUAV)
	*Phlebovirus uriuranaense*	Uriurana virus (URIV)
	*Phlebovirus urucuriense*	Urucuri virus (URUV)
	*Phlebovirus zerdaliense*	Zerdali virus (ZERV)
*Pidchovirus*	*Pidchovirus pidgei*	Pidgey virus (PGYV)
	*Pidchovirus stethori*	coleopteran phenui-related virus 308 (CoPrV308)
*Rubodvirus*	*Rubodvirus argentinaense*	grapevine Muscat rose virus (GMRV)
	*Rubodvirus armeniaense*	grapevine Garan dmak virus (GGDV)
	*Rubodvirus mali*	apple rubbery wood virus 1 (ARWV1)
	*Rubodvirus prosserense*	apple rubbery wood virus 2 (ARWV2)
*Tanzavirus*	*Tanzavirus daressalaamense*	Dar es Salaam virus (DeSV)
*Tenuivirus*	*Tenuivirus echinochloae*	Echinochloa hoja blanca virus (EHBV)
	*Tenuivirus eurotritici*	European wheat striate mosaic virus (EWSMV)
	*Tenuivirus festucae*	Festuca stripe-associated virus (FSaV)
	*Tenuivirus kwazuluense*	wheat yellows virus (WhYV)
	*Tenuivirus oryzabrevis*	rice grassy stunt virus (RGSV)
	*Tenuivirus oryzaclavatae*	rice stripe virus (RSV; RStV)
	*Tenuivirus oryzalbae*	rice hoja blanca virus (RHBV)
	*Tenuivirus persotritici*	Iranian wheat stripe virus (IWSV)
	*Tenuivirus pontaense*	wheat white spike virus (WWSV)
	*Tenuivirus urochloae*	Urochloa hoja blanca virus (UHBV)
	*Tenuivirus zeae*	maize stripe virus (MSV; MStV; MSpV)
*Uukuvirus*	*Uukuvirus dabieshanense*	Dàbiéshān tick virus (DbsTV; DBTV; DTV)
	*Uukuvirus dermacentoris*	American dog tick virus (ADAV)
	*Uukuvirus grandarbaudense*	Grand Arbaud virus (GAV)
	*Uukuvirus hoplandense*	Pacific coast tick virus (PACV)
	*Uukuvirus huangpiense*	Huángpí tick virus 2 (HpTV2)
	*Uukuvirus kabutoense*	Kabuto mountain virus (KAMV)
	*Uukuvirus kaisodiense*	Kaisodi virus (KASDV)
	*Uukuvirus lihanense*	Lǐhán tick virus (LITV)
	*Uukuvirus macquariense*	Precarious Point virus (PPV)
	*Uukuvirus rukutamaense*	Rukutama virus (RUKV)
	*Uukuvirus schmidti*	Nile warbler virus (NIWV)
	*Uukuvirus silverwaterense*	Silverwater virus (SILV)
	*Uukuvirus tachengense*	Tǎchéng tick virus 2 (TcTV2)
	*Uukuvirus toyoense*	Tōyo virus (TOYOV)
	*Uukuvirus tyulenyense*	Zaliv Terpeniya virus (ZTV)
	*Uukuvirus uriae*	murre virus (MURV)
	*Uukuvirus uukuniemiense*	Chizé virus (CHZV)
		Fin V 707 virus (FINV)
		Uukuniemi virus (UUKV)
	*Uukuvirus yongjiaense*	Yǒngjiā tick virus (YONV)
*Wenrivirus*	*Wenrivirus penaei*	Mourilyan virus (MoV)
**Family *Wupedeviridae*** [150]		
*Wumivirus*	*Wumivirus millepedae*	Wǔhàn millipede virus 2 (WhMV2)

Note that viruses are real objects that are assigned to concepts that are called taxa. Species, genera, families and orders are taxa.

* Taxon names are always italicized and always begin with a capital letter.

† Virus names are not italicized and are not capitalized, except if the name or a name component is a proper noun. This column lists the virus names with their correct (lack of) capitalization.

**Table 8.** Taxonomy of the order *Articulavirales* (*Negarnaviricota: Polyploviricotina: Insthoviricetes*) as of April 2024

Genus	Species*	Virus (abbreviation)†
**Family *Amnoonviridae*** [151]		
*Tilapinevirus*	*Tilapinevirus tilapiae*	tilapia lake virus (TiLV)
**Family** ***Orthomyxoviridae***		
*Alphainfluenzavirus*	*Alphainfluenzavirus influenzae*	influenza A virus (FLUAV)
*Betainfluenzavirus*	*Betainfluenzavirus influenzae*	influenza B virus (FLUBV)
*Deltainfluenzavirus*	*Deltainfluenzavirus influenzae*	influenza D virus (FLUDV)
*Gammainfluenzavirus*	*Gammainfluenzavirus influenzae*	influenza C virus (FLUCV)
*Isavirus*	*Isavirus salaris*	infectious salmon anaemia virus (ISAV)
*Mykissvirus*	*Mykissvirus tructae*	rainbow trout orthomyxovirus (RbtOV)
		steelhead trout orthomyxovirus (SttOV1)
*Quaranjavirus*	*Quaranjavirus araguariense*	Araguari virus (ARAV)
	*Quaranjavirus chadense*	Lake Chad virus (LKCV)
	*Quaranjavirus johnstonense*	Johnston Atoll virus (JAV)
	*Quaranjavirus quaranfilense*	Quaranfil virus (QRFV)
	*Quaranjavirus tyulekense*	Tyulek virus (TLKV); Tjuloc virus
	*Quaranjavirus wellfleetense*	Wellfleet Bay virus (WFBV)
*Sardinovirus*	*Sardinovirus pilchardi*	pilchard orthomyxovirus (POMV)
*Thogotovirus*	*Thogotovirus bourbonense*	Bourbon virus (BRBV)
	*Thogotovirus dhoriense*	Dhori virus (DHOV)
	*Thogotovirus josense*	Jos virus (JOSV)
	*Thogotovirus ozense*	Oz virus (OZV)
	*Thogotovirus sinuense*	Sinu virus (SINUV)
	*Thogotovirus thailandense*	Thailand tick thogotovirus (TT-THOV)
	*Thogotovirus thogotoense*	Thogoto virus (THOV)
	*Thogotovirus upoluense*	Upolu virus (UPOV)

Note that viruses are real objects that are assigned to concepts that are called taxa. Species, genera, families and orders are taxa

* Taxon names are always italicized and always begin with a capital letter.

† Virus names are not italicized and are not capitalized, except if the name or a name component is a proper noun. This column lists the virus names with their correct (lack of) capitalization.

**Table 9.** Taxonomy of family *Tosoviridae* (*Negarnaviricota*: unassigned) as of April 2024

Genus	Species*	Virus (abbreviation)†
**Family *Tosoviridae*** [152]		
*Fraservirus*	*Fraservirus testudinis*	turtle fraservirus 1 (TFV1)

Note that viruses are real objects that are assigned to concepts that are called taxa. Species, genera, families and orders are taxa.

* Taxon names are always italicized and always begin with a capital letter.

† Virus names are not italicized and are not capitalized, except if the name or a name component is a proper noun. This column lists the virus names with their correct (lack of) capitalization.

## Supplementary material

10.1099/jgv.0.002077 Uncited Fig. S1.

10.1099/jgv.0.002077 Uncited Supplementary Material 1.

## Abbreviations

HTS: high-throughput sequencing
ICTV: International Committee on Taxonomy of Viruses
RdRP: RNA-directed RNA polymerase
RT-PCR: reverse transcription polymerase chain reaction
SRA: Sequence Read Archive
TSA: transcriptome shotgun assembly
