# Supplementary material for: Annual (2024) taxonomic update of RNA-directed RNA polymerase-encoding negative-sense RNA viruses (realm Riboviria: kingdom Orthornavirae: phylum Negarnaviricota)
Source: J Gen Virol. 2025 Jun 13;106(6):002077. doi: 10.1099/jgv.0.002077 (PMC12282299; doi:10.1099/jgv.0.002077)
Supplement: Uncited Fig. S1. [file jgv-106-02077-s001.pdf]

2024 taxonomy of phylum *Negarnaviricota* (*Riboviria: Orthornavirae*): subphylum *Haploviricotina*

Kuhn JH *et al.* (2024) 2024 taxonomic update of phylum *Negarnaviricota* (*Riboviria: Orthornavirae*), including the large orders *Elliovirales*, *Hareavirales*, and *Mononegavirales*.

Negarnaviricota

Haploviricotina

Polyploviricotina, Unassigned

Chunqiuviricetes

Muvirales

Qinviridae

Yingvirus

Yingvirus beihaiense — Běihǎi sesarmid crab virus 4 (BhSCV4)

Yingvirus charybdis

Yingvirus hubeiense

Yingvirus sanxiaense

Yingvirus shaheense

Yingvirus wenzhouense

Yingvirus xinzhouense

[illegible]

Milneviricetes

Naedrevirales

Aspiviridae

Ophiovirus

Ophiovirus capsici, *Ophiovirus citri*, *Ophiovirus freesiae*, *Ophiovirus lactucae*, *Ophiovirus mirafioriense*, *Ophiovirus ranunculi*, *Ophiovirus tulipae*, *Ophiovirus vaccinii*

[illegible]

Yunchangviricetes

Goujianvirales

Yueviridae

Yuyuevirus

Yuyuevirus beihaiense, *Yuyuevirus shaheense*

[illegible]

Monjiviricetes

Jingchuvirales

Aliusviridae, *Chuviridae*, *Crepuscuviridae*, *Myriaviridae*, *Natareviridae*, *Orthoviridae*, *Lispiviridae*, *Artoviridae*, *Bornaviridae*, *Filoviridae*, *Nyamiviridae*, *Paramyxoviridae*, *Pneumoviridae*, *Rhabdoviridae*, *Sunviridae*, *Xinmoviridae*, *Mymonaviridae*

[illegible]

Mononegavirales

[illegible]

Phylum

Subphylum

Class

Order

Family

Subfamily

Genus

Species

Virus (abbreviation)

[1]Includes: Newcastle disease virus (NDV)

2024 taxonomy of phylum *Negarnaviricota* (*Riboviria: Orthornavirae*): subphylum *Polyploviricotina* and unassigned

Kuhn JH *et al.* (2024) 2024 taxonomic update of phylum *Negarnaviricota* (*Riboviria: Orthornavirae*), including the large orders *Elliovirales*, *Hareavirales*, and *Mononegavirales*.

Negarnaviricota

Haploviricotina

Polyploviricotina

Bunyaviricetes

Elliovirales

Cruliviridae

Lincruvirus
- *Lincruvirus braziliense*
  - Callinectes danae portunibunyavirus 1 (CdPBV1)
- *Lincruvirus europense*
  - European shore crab virus 1 (EscV1)
- *Lincruvirus sinense*
  - Chinese mitten crab virus 1 (CmcV1)
- *Lincruvirus wenlingense*
  - Wēnlǐng crustacean virus 9 (WlCV9)

Fimoviridae

Emaravirus
- *Emaravirus aceris*
  - maple mottle-associated virus (MaMaV)
- *Emaravirus actinidiae*
  - Actinidia chlorotic ringspot-associated virus (AcCRaV)
- *Emaravirus ailanthi*
  - Ailanthus crinkle leaf-associated virus (aCrLaV)
- *Emaravirus artemisiae*
  - Artemisia fimovirus 1 (ArtV1)
- *Emaravirus cajani*
  - pigeonpea sterility mosaic virus 1 (PPSMV1)
- *Emaravirus camelliae*
  - Camellia japonica-associated virus 1 (CjaV1)
- *Emaravirus cercidis*
  - redbud yellow ringspot-associated virus (RYRaV)
- *Emaravirus chrysanthemi*
  - chrysanthemum mosaic-associated virus (ChMaV)
- *Emaravirus cordylinae*
  - ti ringspot-associated virus (TiRSaV)
- *Emaravirus corynocarpi*
  - karaka Ōkahu purepure emaravirus (KOPV)
- *Emaravirus fici*
  - fig mosaic virus (FMV)
- *Emaravirus fraxini*
  - ash shoestring-associated virus (aSaV)
- *Emaravirus idaeobati*
  - raspberry leaf blotch virus (RLBV)
- *Emaravirus illicii*
  - Japanese star anise ringspot-associated virus (JSARaV)
- *Emaravirus kiwii*
  - Actinidia virus 2 (AcV2)
- *Emaravirus kudzu*
  - Pueraria lobata-associated emaravirus (PloaEV)
- *Emaravirus parkinsoniae*
  - palo verde broom virus (PVBV)
- *Emaravirus perillae*
  - Perilla mosaic virus (PerMV)
- *Emaravirus pistaciae*
  - Pistacia virus B (PiVB)
- *Emaravirus populi*
  - aspen mosaic-associated virus (AsMaV)
- *Emaravirus pyri*
  - pear chlorotic leaf spot-associated virus (PCLSaV)
- *Emaravirus quercus*
  - common oak ringspot-associated virus (CORaV)
- *Emaravirus rosae*
  - rose rosette virus (RRV)
- *Emaravirus rubi*
  - blackberry leaf mottle-associated virus (BLMaV)
- *Emaravirus sorbi*
  - European mountain ash ringspot-associated virus (EMARaV)
- *Emaravirus syringae*
  - lilac chlorotic ringspot-associated virus (LiCRaV)
- *Emaravirus toordali*
  - pigeonpea sterility mosaic virus 2 (PPSMV2)
- *Emaravirus tritici*
  - High Plains wheat mosaic virus (HPWMoV)
- *Emaravirus verbanni*
  - Camellia japonica-associated virus 2 (CjaV2)
- *Emaravirus visci*
  - Arceuthobium sichuanense-associated virus 1 (ArSaV1)
- *Emaravirus vitis*
  - Vitis emaravirus (VEV)
- *Emaravirus ziziphi*
  - jujube yellow mottle-associated virus (JYMaV)

Hantaviridae

Actantavirinae

Actinovirus
- *Actinovirus bernense*
  - Bern perch virus (BRPV)
- *Actinovirus halieutaeae*
  - Wēnlǐng minipizza batfish virus (WEMBV)
- *Actinovirus triacanthodis*
  - Wēnlǐng red spikefish virus (WERSV)

Percilovirus
- *Percilovirus gobii*
  - Luposicya lupus actinovirus (LlAV)
- *Percilovirus lophii*
  - Wēnlǐng yellow goosefish virus (WEYGV)

Agantavirinae

Agnathovirus
- *Agnathovirus eptatreti*
  - Wēnlǐng hagfish virus (WEHV)

Mammantavirinae

Loanvirus
- *Loanvirus brunaense*
  - Brno virus (BRNV)
- *Loanvirus longquanense*
  - Lóngquán virus (LQUV)

Mobatvirus
- *Mobatvirus dakrongense*
  - Đakrông virus (DKGV)
- *Mobatvirus laibinense*
  - Láibīn virus (LAIV)
- *Mobatvirus lenaense*
  - Lena virus (LENV)
- *Mobatvirus novaense*
  - Nova virus (NVAV)
- *Mobatvirus quezonense*
  - Quezon virus (QZNV)
- *Mobatvirus robinaense*
  - Robina virus (ROBV)
- *Mobatvirus xuansonense*
  - Xuân Sơn virus (XSV)

Orthohantavirus
- *Orthohantavirus andesense*
  - Andes virus (ANDV)
- *Orthohantavirus artybashense*
  - Artybash virus (ARTV)
- *Orthohantavirus asamaense*
  - Asama virus (ASAV)
- *Orthohantavirus asikkalaense*
  - Asikkala virus (ASIV)
- *Orthohantavirus bayoui*
  - bayou virus (BAYV)
  - Catacamas virus (CATV)
- *Orthohantavirus boweense*
  - Bowé virus (BOWV)
- *Orthohantavirus brugesense*
  - Bruges virus (BRGV)
- *Orthohantavirus caobangense*
  - Cao Bằng virus (CBNV)
- *Orthohantavirus carrizalense*
  - Carrizal virus (CARV)
  - Huitzilac virus (HUIV)
- *Orthohantavirus chocloense*
  - Choclo virus (CHOV)
- *Orthohantavirus dabieshanense*
  - Dàbiéshān virus (DBSV)
- *Orthohantavirus delgaditoense*
  - Caño Delgadito virus (CADV)
- *Orthohantavirus dobravaense*
  - Dobrava virus (DOBV)
  - Kurkino virus (KURV)
  - Saaremaa virus (SAAV)
  - Sochi virus (SOCV)
- *Orthohantavirus fugongense*
  - Fúgòng virus (FUGV)
- *Orthohantavirus hantanense*
  - Amur virus (AMRV)
  - Hantaan virus (HTNV)
  - Soochong virus (SOOV)
- *Orthohantavirus jejuense*
  - Jeju virus (JJUV)
- *Orthohantavirus kenkemeense*
  - Kenkeme virus (KKMV)
- *Orthohantavirus khabarovskense*
  - Khabarovsk virus (KHAV)
  - Topografov virus (TOPV)
- *Orthohantavirus lankaense*
  - Lanka virus (LNKV)
- *Orthohantavirus luxiense*
  - Lúxī virus (LUXV)
- *Orthohantavirus mamorense*
  - Maripa virus (MARV)
  - Rio Mamoré virus (RIOMV)
- *Orthohantavirus maporalense*
  - Maporal virus (MAPV)
- *Orthohantavirus montanoense*
  - Montaño virus (MTNV)
- *Orthohantavirus nigrorivense*
  - Black Creek Canal virus (BCCV)
- *Orthohantavirus prospectense*
  - Prospect Hill virus (PHV)
- *Orthohantavirus puumalaense*
  - Hokkaido virus (HOKV)
  - Muju virus (MUJV)
  - Puumala virus (PUUV)
- *Orthohantavirus rockportense*
  - Rockport virus (RKPV)
- *Orthohantavirus sangassouense*
  - Sangassou virus (SANGV)
- *Orthohantavirus seoulense*
  - Seoul virus (SEOV)
- *Orthohantavirus sinnombreense*
  - New York virus (NYV)
  - Sin Nombre virus (SNV)
- *Orthohantavirus tatenalense*
  - Tatenale virus (TATV)
- *Orthohantavirus thailandense*
  - Anjozorobe virus (ANJZV)
  - Thailand virus (THAIV)
- *Orthohantavirus tigrayense*
  - Tigray virus (TIGV)
- *Orthohantavirus tulaense*
  - Tula virus (TULV)
- *Orthohantavirus wufangense*
  - Wǔfēng Chodsigoa smithii orthohantavirus 1 (WfCsOHV1)

Thottimvirus
- *Thottimvirus imjinense*
  - Imjin virus (MJNV)
- *Thottimvirus thottapalayamense*
  - Thottapalayam virus (TPMV)

Repantavirinae

Reptillovirus
- *Reptillovirus hemidactyli*
  - Hǎinán oriental leaf-toed gecko virus (HOLGV)

Peribunyaviridae

Gryffinivirus
- *Gryffinivirus alamedaense*
  - Udune virus (UDNV)
- *Gryffinivirus hedwigae*
  - Hedwig virus (HEDV)

Herbevirus
- *Herbevirus herberti*
  - Herbert virus (HEBV)
- *Herbevirus kibaleense*
  - Kibale virus (KIBV)
- *Herbevirus taiense*
  - Taï virus (TAIV)

Khurdivirus
- *Khurdivirus volgaense*
  - Khurdun virus (KURV)

Lakivirus
- *Lakivirus lakamhaense*
  - Lakamha virus (LAKV)

Lambavirus
- *Lambavirus wisconsinense*
  - largemouth bass bunyavirus (LBBV)

Orthobunyavirus
- *Orthobunyavirus abrasense*
  - Abras virus (ABRV)
- *Orthobunyavirus acaraense*
  - Acará virus (ACAV)
  - Benevides virus (BVSV; BENV)
- *Orthobunyavirus achiotei*
  - Achiote virus (ACHOV)
  - trivittatus virus (TVTV)
- *Orthobunyavirus ainoense*
  - Aino virus (AINOV)
  - Kaikalur virus (KAIV)
- *Orthobunyavirus akabaneense*
  - Akabane virus (AKAV)
  - Tinaroo virus (TINV)
- *Orthobunyavirus anadyrense*
  - Anadyr virus (ANADV)
- *Orthobunyavirus ananindeuaense*
  - Ananindeua virus (ANUV)
- *Orthobunyavirus angeloense*
  - San Angelo virus (SAV)
- *Orthobunyavirus anhembiense*
  - Anhembi virus (AMBV)
- *Orthobunyavirus apeuense*
  - Apeú virus (APEUV)
- *Orthobunyavirus baakalense*
  - Baakal virus (BKAV)
- *Orthobunyavirus bakauense*
  - Bakau virus (BAKV)
  - Tanjong Rabok virus (TRV)
- *Orthobunyavirus balagoduense*
  - Balagodu virus (BLGV)
- *Orthobunyavirus barritaense*
  - Barrita virus (BITV)
- *Orthobunyavirus bataiense*
  - Batai virus (BATV)
- *Orthobunyavirus batamaense*
  - Batama virus (BMAV)
- *Orthobunyavirus belemense*
  - Belém virus (BLMV)
- *Orthobunyavirus bellavistaense*
  - Antequera virus (ANTV)
  - Barranqueras virus (BQSV)
  - Bellavista virus (BELLV)
  - Resistencia virus (RTAV)
- *Orthobunyavirus belmontense*
  - Belmont virus (BELV)
- *Orthobunyavirus benficaense*
  - Benfica virus (BENV; BNFV)
- *Orthobunyavirus bertiogaense*
  - Bertioga virus (BERV)
  - Cananéia virus (CNAV)
- *Orthobunyavirus biraoense*
  - Birao virus (BIRV)
- *Orthobunyavirus bobayaense*
  - Bobaya virus (BOBV)
- *Orthobunyavirus boraceiaense*
  - Boracéia virus (BORV)
- *Orthobunyavirus botambiense*
  - Botambi virus (BOTV)
- *Orthobunyavirus bozoense*
  - Bozo virus (BOZOV)
- *Orthobunyavirus brazoriaense*
  - brazoran virus (BRAZV)
- *Orthobunyavirus bruconhaense*
  - Bruconha virus (BRUV)
- *Orthobunyavirus buffaloense*
  - Buffalo Creek virus (BUCV)
  - Murrumbidgee virus (MURBV)
  - Trubanaman virus (TRUV)
- *Orthobunyavirus bunyamweraense*
  - Bunyamwera virus (BUNV)
  - Germiston virus (GERV)
  - Mboké virus (MBOV)
  - Ngari virus (NRIV)
  - Stanfield virus (STAV)
  - Xingu virus (XINV)
- *Orthobunyavirus bushbushense*
  - Bushbush virus (BSBV)
  - Moriche virus (MORV)
- *Orthobunyavirus buttonwillowense*
  - Buttonwillow virus (BUTV)
- *Orthobunyavirus bwambaense*
  - Bwamba virus (BWAV)
  - Pongola virus (PGAV)
- *Orthobunyavirus cacheense*
  - Cache Valley virus (CVV)
  - Cholul virus (CHLV)
  - Playas virus (PLAV)
  - Tlacotalpan virus (TLAV)
- *Orthobunyavirus capimense*
  - Capim virus (CAPV)
- *Orthobunyavirus caraparuense*
  - Caraparú virus (CARV)
  - El Huayo virus (EHUV)
  - Itaquí virus (ITQV)
  - Itaya virus (ITYV)
  - Ossa virus (OSSAV)
- *Orthobunyavirus catqueense*
  - Cát Quế virus (CQV)
  - Oya virus (OYAV)
- *Orthobunyavirus catuense*
  - Catú virus (CATUV)
  - Moju virus (MOJUV)
- *Orthobunyavirus cuchillaense*
  - Anopheles B virus (ANBV)
- *Orthobunyavirus ebiense*
  - Ebinur Lake virus (EBIV)
- *Orthobunyavirus encephalitidis*
  - California encephalitis virus (CEV)
  - Morro Bay virus (MBV)
- *Orthobunyavirus enseadaense*
  - Enseada virus (ENSV)
- *Orthobunyavirus faceyense*
  - Facey's paddock virus (FPV)
- *Orthobunyavirus gamboaense*
  - Alajuela virus (ALJV)
  - Brus Laguna virus (BLAV)
  - Calchaquí virus (CQIV)
  - Gamboa virus (GAMV)
  - Soberanía virus (SOBV)
- *Orthobunyavirus ganganense*
  - Gan Gan virus (GGV)
  - Salt Ash virus (SASHV)
- *Orthobunyavirus guajaraense*
  - Guajará virus (GJAV)
- *Orthobunyavirus guamaense*
  - Bimiti virus (BIMV)
  - Guamá virus (GMAV)
- *Orthobunyavirus guaratubaense*
  - Guaratuba virus (GTBV)
  - Itimirim virus (ITIV)
- *Orthobunyavirus guaroaense*
  - Guaroa virus (GROV)
- *Orthobunyavirus gumbolimboense*
  - Gumbo Limbo virus (GLV)
- *Orthobunyavirus heptayabaense*
  - Yaba-7 virus (Y7V)
- *Orthobunyavirus horizonteense*
  - Anopheles A virus (ANAV)
  - Arumateua virus (ARTV; ARMTV)
  - Caraipé virus (CPEV; CRPV)
  - Trombetas virus (TRMV)
  - Tucuruí virus (TUCV; TUCRV)
- *Orthobunyavirus iacoense*
  - Iaco virus (IACOV)
- *Orthobunyavirus ileshaense*
  - Ilesha virus (ILEV)
- *Orthobunyavirus infirmati*
  - Infirmatus virus (INFV)
- *Orthobunyavirus ingwavumaense*
  - Ingwavuma virus (INGV)
- *Orthobunyavirus insulae*
  - kairi virus (KRIV)
- *Orthobunyavirus jamestownense*
  - Inkoo virus (INKV)
  - Jamestown Canyon virus (JCV)
  - Jerry Slough virus (JSV)
  - South River virus (SORV)
- *Orthobunyavirus jatobalense*
  - Jatobal virus (JATV)
- *Orthobunyavirus juandiazense*
  - Juan Díaz virus (JDV)
- *Orthobunyavirus kaengkhoiense*
  - Kaeng Khoi virus (KKV)
- *Orthobunyavirus kernense*
  - Lokern virus (LOKV)
  - Main Drain virus (MDV)
  - Santa Rosa virus (SARV)
- *Orthobunyavirus ketapangense*
  - Ketapang virus (KETV)
- *Orthobunyavirus keystoneense*
  - Keystone virus (KEYV)
- *Orthobunyavirus khatangaense*
  - Khatanga virus (KHATV)
  - snowshoe hare virus (SSHV)
- *Orthobunyavirus koongoli*
  - koongol virus (KOOV)
  - wongal virus (WONV)
- *Orthobunyavirus kowanyamaense*
  - Kowanyama virus (KOWV)
- *Orthobunyavirus lacrosseense*
  - La Crosse virus (LACV)
- *Orthobunyavirus lasmaloyasense*
  - Las Maloyas virus (LMV)
- *Orthobunyavirus leanyerense*
  - Leanyer virus (LEAV)
- *Orthobunyavirus ledniceense*
  - Lednice virus (LEDV)
- *Orthobunyavirus lukuniense*
  - Lukuni virus (LUKV)
- *Orthobunyavirus lumboense*
  - Lumbo virus (LUMV)
- *Orthobunyavirus macauaense*
  - Macauã virus (MCAV)
- *Orthobunyavirus madridense*
  - Madrid virus (MADV)
  - Vinces virus (VINV)
- *Orthobunyavirus maguariense*
  - Maguari virus (MAGV)
- *Orthobunyavirus mahoganyense*
  - Mahogany Hammock virus (MHV)
- *Orthobunyavirus manzanillaense*
  - Manzanilla virus (MANV)
  - Inini virus (INIV)
- *Orthobunyavirus maprikense*
  - Maprik virus (MPKV)
- *Orthobunyavirus marituba*ense
  - Marituba virus (MTBV)
  - Zungarococha virus (ZUNV)
- *Orthobunyavirus matruhense*
  - Matruh virus (MTRV)
- *Orthobunyavirus melajoense*
  - Melao virus (MELV)
- *Orthobunyavirus mermetense*
  - Mermet virus (MERV)
- *Orthobunyavirus minatitlanense*
  - Minatitlán virus (MNTV)
  - Palestina virus (PLSV)
- *Orthobunyavirus mirimense*
  - Mirim virus (MIRV)
- *Orthobunyavirus mitchellense*
  - Mapputta virus (MAPV)
- *Orthobunyavirus mpokoense*
  - M'Poko virus (MPOV)
  - Yaba-1 virus (Y1V)
- *Orthobunyavirus navioense*
  - Serra do Navio virus (SDNV)
- *Orthobunyavirus nepuyoi*
  - Nepuyo virus (NEPV)
- *Orthobunyavirus nesszionaense*
  - Ness Ziona virus (NZV)
- *Orthobunyavirus nolaense*
  - Nola virus (NOLAV)
- *Orthobunyavirus northwayense*
  - Northway virus (NORV)
- *Orthobunyavirus nyandoense*
  - Eretmapodites virus (ERETV)
  - Mojuí dos Campos virus (MDCV)
  - Nyando virus (NDV)
- *Orthobunyavirus okolaense*
  - Okola virus (OKOV)
- *Orthobunyavirus olifantsvleiense*
  - Bobia virus (BIAV)
  - Dabakala virus (DABV)
  - Olifantsvlei virus (OLIV)
  - Oubi virus (OUBIV)
- *Orthobunyavirus oribocaense*
  - Murutucú virus (MURV)
  - Oriboca virus (ORIV)
  - Restan virus (RESV)
- *Orthobunyavirus oropoucheense*
  - Iquitos virus (IQTV)
  - Madre de Dios virus (MDDV)
  - Oropouche virus (OROV)
  - Perdões virus (PDEV)
  - Pintupo virus (PINTV)
- *Orthobunyavirus oyoense*
  - Oyo virus (OYOV)
- *Orthobunyavirus pacoraense*
  - Pacora virus (PCAV)
- *Orthobunyavirus parkeri*
  - Parker's Farm virus (PFV)
- *Orthobunyavirus patoisense*
  - Babahoya virus (BABV)
  - Patois virus (PATV)
- *Orthobunyavirus peachesterense*
  - peaton virus (PEAV)
- *Orthobunyavirus porteiraense*
  - Cachoeira Porteira virus (CPOV)
- *Orthobunyavirus potosiense*
  - Potosi virus (POTV)
- *Orthobunyavirus saboense*
  - Sabo virus (SABOV)
- *Orthobunyavirus sangoense*
  - Sango virus (SANV)
- *Orthobunyavirus sanjuanense*
  - Pueblo Viejo virus (PVV)
  - San Juan virus (SJV)
- *Orthobunyavirus schmallenbergense*
  - Douglas virus (DOUV)
  - Sathuperi virus (SATV)
  - Schmallenberg virus (SBV)
  - Shamonda virus (SHAV)
- *Orthobunyavirus sedlecense*
  - Sedlec virus (SEDV)
- *Orthobunyavirus shermanense*
  - Fort Sherman virus (FSV)
  - Laguna Larga virus (LLV)
- *Orthobunyavirus shokweense*
  - Shokwe virus (SHOV)
- *Orthobunyavirus shuniense*
  - Shuni virus (SHUV)
- *Orthobunyavirus simbuense*
  - Para virus (PARAV)
  - Simbu virus (SIMV)
- *Orthobunyavirus sororocaense*
  - Sororoca virus (SORV)
- *Orthobunyavirus squalofluvii*
  - Shark River virus (SRV)
- *Orthobunyavirus sussexense*
  - Little Sussex virus (LTLSV)
- *Orthobunyavirus tacaiumaense*
  - CoAr 1071 virus (CA1071V)
  - CoAr 3627 virus (CA3626V)
  - Tacaiuma virus (TCMV)
  - Virgin River virus (VRV)
- *Orthobunyavirus tahynaense*
  - Ťahyňa virus (TAHV)
- *Orthobunyavirus tangaense*
  - Tanga virus (TANV)
- *Orthobunyavirus tataguineense*
  - Tataguine virus (TATV)
- *Orthobunyavirus telokense*
  - Telok Forest virus (TFV)
- *Orthobunyavirus tensawense*
  - Tensaw virus (TENV)
- *Orthobunyavirus termeilense*
  - Termeil virus (TERV)
- *Orthobunyavirus teteense*
  - Bahig virus (BAHV)
  - Tete virus (TETEV)
  - Tsuruse virus (TSUV)
  - Weldona virus (WELV)
- *Orthobunyavirus thimiriense*
  - Thimiri virus (THIV)
- *Orthobunyavirus timboteuaense*
  - Timboteua virus (TBTV)
- *Orthobunyavirus trinitiense*
  - Triniti virus (TNTV)
- *Orthobunyavirus turlockense*
  - Turlock virus (TURV)
- *Orthobunyavirus umbreense*
  - Umbre virus (UMBV)
- *Orthobunyavirus utingaense*
  - Utinga virus (UTIV)
- *Orthobunyavirus witwatersrandense*
  - Witwatersrand virus (WITV)
- *Orthobunyavirus wolkbergense*
  - Wolkberg virus (WBV)
- *Orthobunyavirus wyeomyiae*
  - Rio Pracupi virus
  - Taiassui virus (TAIAV)
  - Tucunduba virus (TUCV)
  - Wyeomyia virus (WYOV)
- *Orthobunyavirus yacaabaense*
  - Yacaaba virus (YACV)

Pacuvirus
- *Pacuvirus caimitoense*
  - Caimito virus (CAIV)
- *Pacuvirus chilibreense*
  - Chilibre virus (CHIV)
- *Pacuvirus evaense*
  - Rio Preto da Eva virus (RPEV)
- *Pacuvirus pacuiense*
  - Pacui virus (PACV)
- *Pacuvirus tapirapeense*
  - Tapirapé virus (TAPV)

Shangavirus
- *Shangavirus shuangaoense*
  - Shuāngào insect virus 1 (SgIV1)

Phasmaviridae

Cicadellivirus
- *Cicadellivirus scaphoidei*
  - Scaphoideus titanus bunya-like virus 1 (StHV1)

Feravirus
- *Feravirus ferakinum*
  - Ferak virus (FRKV)
- *Feravirus guaguaense*
  - Guagua virus (GUAV)
- *Feravirus hemipterus*
  - hemipteran phasma-related virus OKIAV247 (HeFV)
- *Feravirus neuropterus*
  - neuropteran phasma-related virus OKIAV248 (NeFV)

Hymovirus
- *Hymovirus chrysis*
  - hymenopteran phasma-related virus OKIAV250 (HyHV2)
- *Hymovirus chrysurae*
  - hymenopteran phasma-related virus OKIAV252 (HyHV1)

Jonvirus
- *Jonvirus eboris*
  - jonchet virus (JONV)

Orthophasmavirus
- *Orthophasmavirus aedis*
  - Wǔhàn mosquito virus 2 (WhMV2)
- *Orthophasmavirus anophelae*
  - Anopheles triannulatus orthophasmavirus (AtOPV)
- *Orthophasmavirus barstukasense*
  - Barstukas virus (BARV)
- *Orthophasmavirus chrysis*
  - hymenopteran phasma-related virus OKIAV227 (HyOV2)
- *Orthophasmavirus coleopterus*
  - coleopteran phasma-related virus OKIAV235 (CPRV)
- *Orthophasmavirus coredoense*
  - Coredo virus (CORV)
- *Orthophasmavirus culicis*
  - Culex phasma-like virus (CPLV)
- *Orthophasmavirus flenense*
  - Flen virus (FLNV)
- *Orthophasmavirus fushunense*
  - Fǔshùn phasmavirus 2 (FsnPV2)
- *Orthophasmavirus gandaense*
  - Ganda bee virus (GBEEV)
- *Orthophasmavirus hubeiense*
  - Húběi odonate virus 9 (HbOV9)
- *Orthophasmavirus kigluaikense*
  - Kigluaik phantom virus (KIGV)
- *Orthophasmavirus miglotasense*
  - miglotas virus (MIGV)
- *Orthophasmavirus niuklukense*
  - Niukluk phantom virus (NUKV)
- *Orthophasmavirus odonatus*
  - Húběi odonate virus 8 (HbOV8)
- *Orthophasmavirus philoctetis*
  - hymenopteran phasma-related virus OKIAV228 (HyOV1)
- *Orthophasmavirus sogatellae*
  - Fǔshùn phasmavirus 1 (FsnPV1)
- *Orthophasmavirus wuchangense*
  - Wǔchāng cockroach virus 1 (WcCV1)
- *Orthophasmavirus wuhanense*
  - Wǔhàn mosquito virus 1 (WhMV1)

Sawastrivirus
- *Sawastrivirus sanxiaense*
  - Sānxiá water strider virus 2 (SxWSV2)

Wuhivirus
- *Wuhivirus insecti*
  - Wǔhàn insect virus 2 (WhIV2)

Tospoviridae

Orthotospovirus
- *Orthotospovirus alstroemeriflavi*
  - Alstroemeria yellow spot virus (AYSV)
- *Orthotospovirus alstroemerinecrosis*
  - Alstroemeria necrotic streak virus (ANSV)
- *Orthotospovirus arachianuli*
  - groundnut ringspot virus (GRSV)
- *Orthotospovirus arachiflavamaculae*
  - groundnut yellow spot virus (GYSV)
- *Orthotospovirus arachiflavi*
  - groundnut chlorotic fan-spot virus (GCFSV)
- *Orthotospovirus arachinecrosis*
  - groundnut bud necrosis virus (GBNV)
- *Orthotospovirus callaflavi*
  - calla lily chlorotic spot virus (CCSV)
- *Orthotospovirus capsiciflavi*
  - Capsicum chlorosis virus (CaCV)
- *Orthotospovirus capsicimaculaflavi*
  - pepper chlorotic spot virus (PCSV)
- *Orthotospovirus chrysanthinecrocaulis*
  - Chrysanthemum stem necrosis virus (CSNV)
- *Orthotospovirus citrullomaculosi*
  - watermelon silver mottle virus (WSMoV)
- *Orthotospovirus citrullonecrosis*
  - watermelon bud necrosis virus (WBNV)
- *Orthotospovirus cucurbichlorosis*
  - zucchini lethal chlorosis virus (ZLCV)
- *Orthotospovirus glycininecrovenae*
  - soybean vein necrosis virus (SVNV)
- *Orthotospovirus hippeflavi*
  - Hippeastrum chlorotic spot virus (HCRV)
- *Orthotospovirus impatiensnecromaculae*
  - impatiens necrotic spot virus (INSV)
- *Orthotospovirus iridimaculaflavi*
  - iris yellow spot virus (IYSV)
- *Orthotospovirus meloflavi*
  - melon yellow spot virus (MYSV)
- *Orthotospovirus melotessellati*
  - melon severe mosaic virus (MSMV)
- *Orthotospovirus morivenae*
  - mulberry vein banding-associated virus (MVBaV)
- *Orthotospovirus phaseolinecrotessellati*
  - bean necrotic mosaic virus (BeNMV)
- *Orthotospovirus polygonianuli*
  - Polygonum ringspot virus (PolRSV)
- *Orthotospovirus tomatoanuli*
  - tomato yellow ring virus (TYRV)
- *Orthotospovirus tomatoflavi*
  - tomato chlorotic spot virus (TCSV)
- *Orthotospovirus tomatomaculae*
  - tomato spotted wilt virus (TSWV)
- *Orthotospovirus tomatozonae*
  - tomato zonate spot virus (TZSV)

Tulasviridae

Orthotulasvirus
- *Orthotulasvirus tulasnellae*
  - Tulasnella bunyavirales-like virus 1 (TB-LV)

Paratulasvirus
- *Paratulasvirus agrocybes*
  - Agrocybe praecox tulasvirus 1 (ApTV1)

Hareavirales

Arenaviridae

Antennavirus
- *Antennavirus hirsutum*
  - Wēnlǐng frogfish arenavirus 2 (WlFAV2)
- *Antennavirus salmonis*
  - salmon pescarenavirus 1 (SPAV1)
  - salmon pescarenavirus 2 (SPAV2)
- *Antennavirus striale*
  - Wēnlǐng frogfish arenavirus 1 (WlFAV1)

Hartmanivirus
- *Hartmanivirus brazilense*
  - SetPatVet virus 1 (SPVV1)
- *Hartmanivirus haartmani*
  - Haartman Institute snake virus 1 (HISV1)
  - Haartman Institute snake virus 2 (HISV2)
- *Hartmanivirus helvetiae*
  - Dante Muikkunen virus 1 (DaMV1)
- *Hartmanivirus patriae*
  - andere Heimat virus 1 (aHeV1)
- *Hartmanivirus quadrati*
  - big electron-dense squares virus 1 (BESV1)
- *Hartmanivirus scholae*
  - old schoolhouse virus 1 (OScV1)
  - old schoolhouse virus 2 (OScV2)
- *Hartmanivirus turici*
  - veterinary pathology Zurich virus 1 (VPZV1)
  - veterinary pathology Zurich virus 2 (VPZV2)
- *Hartmanivirus unni*
  - Universidad Nacional virus 1 (UnNV1)

Innmovirus
- *Innmovirus hailarense*
  - Hailar virus (HLRV)

Mammarenavirus
- *Mammarenavirus abaense*
  - Àbà-Miányáng virus (AMYV)
- *Mammarenavirus alashanense*
  - Alxa virus (ALXV)
- *Mammarenavirus allpahuayoense*
  - Allpahuayo virus (ALLV)
- *Mammarenavirus amapariense*
  - Amaparí virus (AMAV)
- *Mammarenavirus aporeense*
  - Aporé virus (APOV)
- *Mammarenavirus batangense*
  - Bātáng virus (BTTV)
- *Mammarenavirus bearense*
  - Bear Canyon virus (BCNV)
- *Mammarenavirus beregaense*
  - Berega virus (BEGV)
- *Mammarenavirus bituense*
  - Bitu virus (BITV)
- *Mammarenavirus brazilense*
  - Sabiá virus (SBAV)
- *Mammarenavirus caliense*
  - Pichindé virus (PICHV)
- *Mammarenavirus cameroonense*
  - souris virus (SOUV)
- *Mammarenavirus chapareense*
  - Chapare virus (CHAPV)
- *Mammarenavirus choriomeningitidis*
  - Dandenong virus (DANV)
  - lymphocytic choriomeningitis virus (LCMV)
- *Mammarenavirus cupixiense*
  - Cupixi virus (CUPXV)
- *Mammarenavirus dhati-welelense*
  - Dhati Welel virus (DHWV)
- *Mammarenavirus flexalense*
  - Flexal virus (FLEV)
- *Mammarenavirus gairoense*
  - Gairo virus (GAIV)
- *Mammarenavirus ganziense*
  - Gānzī virus (GNZV)
- *Mammarenavirus guanaritoense*
  - Guanarito virus (GTOV)
- *Mammarenavirus ippyense*
  - Ippy virus (IPPYV)
- *Mammarenavirus juninense*
  - Junín virus (JUNV)
- *Mammarenavirus kitaleense*
  - Kitale virus (KTLV)
- *Mammarenavirus kwanzaense*
  - Kwanza virus (KWAV)
- *Mammarenavirus lassaense*
  - Lassa virus (LASV)
- *Mammarenavirus latinum*
  - Latino virus (LATV)
- *Mammarenavirus lijiangense*
  - Lìjiāng virus (LIJV)
- *Mammarenavirus loeiense*
  - Loei River virus (LORV)
- *Mammarenavirus lujoense*
  - Lujo virus (LUJV)
- *Mammarenavirus lunaense*
  - Luli virus (LULV)
  - Luna virus (LUAV)
- *Mammarenavirus lunkense*
  - Lunk virus (LNKV)
- *Mammarenavirus machupoense*
  - Machupo virus (MACV)
- *Mammarenavirus mafigaense*
  - Mafiga virus (MAFV)
- *Mammarenavirus marientalense*
  - Mariental virus (MRLV)
- *Mammarenavirus mecsekense*
  - Mecsek Mountains virus (MEMV)
- *Mammarenavirus merinoense*
  - Merino Walk virus (MRWV)
- *Mammarenavirus mopeiaense*
  - Mopeia virus (MPOV)
  - Morogoro virus (MORV)
- *Mammarenavirus ngerengerense*
  - Ngerengere virus (NGEV)
- *Mammarenavirus okahandjaense*
  - Okahandja virus (OKAV)
- *Mammarenavirus oliverosense*
  - Oliveros virus (OLVV)
- *Mammarenavirus paranaense*
  - Paraná virus (PRAV)
- *Mammarenavirus piritalense*
  - Pirital virus (PIRV)
- *Mammarenavirus praomyidis*
  - mobala virus (MOBV)
- *Mammarenavirus ryukyuense*
  - Ryukyu virus (RYKV)
- *Mammarenavirus solweziense*
  - Solwezi virus (SOLV)
- *Mammarenavirus songeaense*
  - Songea virus (SOGV)
- *Mammarenavirus tacaribeense*
  - Tacaribe virus (TCRV)
- *Mammarenavirus tamiamiense*
  - Tamiami virus (TMMV)
- *Mammarenavirus tietense*
  - Tietê virus (TIEV)
- *Mammarenavirus wenzhouense*
  - Wēnzhōu virus (WENV)
- *Mammarenavirus whitewaterense*
  - Big Brushy Tank virus (BBRTV)
  - Catarina virus (CTNV)
  - Skinner Tank virus (SKTV)
  - Tonto Creek virus (TTCV)
  - Whitewater Arroyo virus (WWAV)
- *Mammarenavirus xapuriense*
  - Xapuri virus (XAPV)

Reptarenavirus
- *Reptarenavirus aurei*
  - Golden Gate virus (GOGV)
- *Reptarenavirus californiae*
  - CAS virus (CASV)
- *Reptarenavirus commune*
  - tavallinen suomalainen mies virus 2 (TSMV2)
- *Reptarenavirus giessenae*
  - University of Giessen virus 1 (UGV1)
  - University of Giessen virus 2 (UGV2)
  - University of Giessen virus 3 (UGV3)
- *Reptarenavirus rotterdamense*
  - ROUT virus (ROUTV)
  - University of Helsinki virus 1 (UHV1)

Discoviridae

Orthodiscovirus
- *Orthodiscovirus coniellae*
  - Coniothyrium diplodiella negative-stranded RNA virus 1 (CdNSRV1)
  - Plasmopara viticola lesion associated mycobunyavirales-like virus 9 (PvLAMLV9)
- *Orthodiscovirus hispaniae*
  - Plasmopara viticola lesion associated mycobunyavirales-like virus 8 (PvLAMLV8)
- *Orthodiscovirus iberiae*
  - Plasmopara viticola lesion associated mycobunyavirales-like virus 4 (PvLAMLV4)
- *Orthodiscovirus missouriense*
  - Penicillium discovirus (PDV)
- *Orthodiscovirus oryzae*
  - rice dwarf-associated bunya-like virus (RDaBV)
- *Orthodiscovirus penicillii*
  - Penicillium roseopurpureum negative ssRNA virus 1 (PrNssV1)

Konkoviridae

Olpivirus
- *Olpivirus tulipae*
  - tulip streak virus (TuSV)

Leishbuviridae

Shilevirus
- *Shilevirus leptomonadis*
  - Leptomonas moramango virus (LEPMV)

Mypoviridae

Hubavirus
- *Hubavirus myriapedis*
  - Húběi myriapoda virus 5 (HbMV5)

Nairoviridae

Norwavirus
- *Norwavirus beijiense*
  - Běijí nairovirus (BJNV)
- *Norwavirus grotenhoutense*
  - Grotenhout virus (GRHV)

Ocetevirus
- *Ocetevirus paratemnopterygis*
  - red goblin roach virus 1 (RGRV1)

Orthonairovirus
- *Orthonairovirus abuhammadense*
  - Abū Ḥammād virus (AHV)
- *Orthonairovirus abuminaense*
  - Abū Mīnā virus (AMV)
- *Orthonairovirus amblyommae*
  - kupe virus (KUPEV)
- *Orthonairovirus antuense*
  - Āntú virus (ANTV)
- *Orthonairovirus artashatense*
  - Artashat virus (ARTSV)
- *Orthonairovirus australiaense*
  - Vinegar Hill virus (VINHV)
- *Orthonairovirus avalonense*
  - Avalon virus (AVAV)
- *Orthonairovirus bandiaense*
  - Bandia virus (BDAV)
- *Orthonairovirus buranaense*
  - Burana virus (BURV)
- *Orthonairovirus bushkeyense*
  - Caspiy virus (CASV)
  - Farallon virus (FARV)
  - Great Saltee virus (GRSV)
  - Hughes virus (HUGV)
  - Raza virus (RAZAV)
- *Orthonairovirus chimiense*
  - Chim virus (CHIMV)
- *Orthonairovirus clomorense*
  - Clo Mor virus (CMV; CLMV)
- *Orthonairovirus crocidurae*
  - Wǔfēng Crocidura attenuata orthonairovirus 1 (WfCAV1)
- *Orthonairovirus dermacentoris*
  - Pacific Coast tick nairovirus (PCTNV)
- *Orthonairovirus dugbeense*
  - Dugbe virus (DUGV)
- *Orthonairovirus erveense*
  - Erve virus (ERVEV)
- *Orthonairovirus esteroense*
  - Estero Real virus (ERV)
- *Orthonairovirus gossasense*
  - Gossas virus (GOSV)
- *Orthonairovirus gubboense*
  - Gubbo nairovirus (GUBV)
- *Orthonairovirus haemorrhagiae*
  - Crimean-Congo hemorrhagic fever virus (CCHFV)
- *Orthonairovirus hazaraense*
  - Hazara virus (HAZV)
- *Orthonairovirus huangpiense*
  - Huángpí tick virus 1 (HpTV1)
- *Orthonairovirus issykkulense*
  - Issyk-kul virus (ISKV)
- *Orthonairovirus japonicum*
  - tofla virus (TFLV)
- *Orthonairovirus kasokeroense*
  - Kasokero virus (KASV; KASOV)
- *Orthonairovirus keterehense*
  - Keterah virus (KTRV)
  - Uzun-Agach virus (UZAV)
- *Orthonairovirus khani*
  - Dera Ghazi Khan virus (DGKV)
- *Orthonairovirus lambarenense*
  - lamusara virus (LMSV)
- *Orthonairovirus lusakaense*
  - Leopards Hill virus (LPHV)
- *Orthonairovirus macquariense*
  - Taggert virus (TAGV)
- *Orthonairovirus manidae*
  - pangolin orthonairovirus (PONV)
- *Orthonairovirus meihuashanense*
  - Méihuā Mountain virus (MHMV)
- *Orthonairovirus meramense*
  - Meram virus (MEMV)
- *Orthonairovirus nairobiense*
  - Nairobi sheep disease virus (NSDV)
- *Orthonairovirus parahaemorrhagiae*
  - Aigai virus (AIGV)
- *Orthonairovirus peruense*
  - Punta Salinas virus (PSV)
- *Orthonairovirus qalyubense*
  - Geran virus (GERV)
  - Qalyub virus (QYBV)
- *Orthonairovirus randallense*
  - Sapphire II virus (SAPV)
- *Orthonairovirus sakhalinense*
  - Sakhalin virus (SAKV)
  - Tillamook virus (TILLV)
- *Orthonairovirus sinense*
  - wawsin virus (WAWV)
- *Orthonairovirus soldadoense*
  - Soldado virus (SOLV)
- *Orthonairovirus songlingense*
  - Sōnglǐng virus (SGLV)
- *Orthonairovirus sulinaense*
  - Sulina virus (SULV)
- *Orthonairovirus sunci*
  - cencurut virus (CENV)
- *Orthonairovirus tachengense*
  - Tǎchéng tick virus 1 (TcTV1)
- *Orthonairovirus thiaforaense*
  - Thiafora virus (TFAV)
- *Orthonairovirus tomdiense*
  - Tamdy virus (TAMV)
- *Orthonairovirus tunisense*
  - Tunis virus (TUNV)
- *Orthonairovirus wenzhouense*
  - Wēnzhōu tick virus (WzTV)
- *Orthonairovirus yezoense*
  - Yezo virus (YEZV)
- *Orthonairovirus yogueense*
  - Yogue virus (YOGV)
- *Orthonairovirus zirkuense*
  - Zirqa virus (ZIRV)

Sabavirus
- *Sabavirus americanum*
  - South Bay virus (SBV)

Shaspivirus
- *Shaspivirus aranei*
  - Shāyáng spider virus 1 (SySV1)

Sitinavirus
- *Sitinavirus sichuanense*
  - Sìchuān tick nairovirus (ScTNV)

Striwavirus
- *Striwavirus sanxiaense*
  - Sānxiá water strider virus 1 (SxWSV1)

Xinspivirus
- *Xinspivirus xinzhouense*
  - Xīnzhōu spider virus (XSV)

Phenuiviridae

Bandavirus
- *Bandavirus albatrossense*
  - Albatross Island virus (ABIV)
- *Bandavirus amblyommae*
  - lone star virus (LSV)
- *Bandavirus bhanjanagarense*
  - Bhanja virus (BHAV)
- *Bandavirus dabieense*
  - severe fever with thrombocytopenia syndrome virus (SFTSV)
- *Bandavirus guertuense*
  - Gùěrtú virus (GTV)
- *Bandavirus heartlandense*
  - Heartland virus (HRTV)
- *Bandavirus kismaayoense*
  - Kismaayo virus (KISV)
- *Bandavirus razdanense*
  - Razdan virus (RAZV)
- *Bandavirus zwieselense*
  - Zwiesel bat bandavirus (ZbbV)

Beidivirus
- *Beidivirus muscae*
  - Húběi diptera virus 3 (HbDV3)

Bocivirus
- *Bocivirus botrytidis*
  - Botrytis cinerea bocivirus 1 (BcBV1)
- *Bocivirus fusarii*
  - Fusarium sibiricum coguvirus 1 (FsCV1)
- *Bocivirus trichodermae*
  - Trichoderma gamsii cogu-like virus 1 (TgCLV1)
- *Bocivirus viticulum*
  - grapevine associated cogu-like virus 1 (GaCLV1)

Citricivirus
- *Citricivirus chongqinense*
  - Aphis citricidus bunyavirus (AcBV)

Coguvirus
- *Coguvirus chinense*
  - Brassica campestris chinensis coguvirus 1 (BCCoV1)
- *Coguvirus chrysanthae*
  - Edgeworthia chrysantha mosaic-associated virus (ECMaV)
- *Coguvirus citri*
  - citrus concave gum-associated virus (CCGaV)
- *Coguvirus citrulli*
  - watermelon crinkle leaf-associated virus 1 (WCLaV1)
- *Coguvirus eburi*
  - citrus virus A (CiVA)
- *Coguvirus henanense*
  - watermelon crinkle leaf-associated virus 2 (WCLaV2)
- *Coguvirus yunnanense*
  - Yúnnán Paris negative-stranded virus (YPNSV)

Entovirus
- *Entovirus entoleucae*
  - Entoleuca phenui-like virus 1 (EnPLV1)

Goukovirus
- *Goukovirus aphalarae*
  - Aphalara polygoni bunya-like virus (ApBLV)
- *Goukovirus ceraphri*
  - Ceraphron bunya-like virus (CerBLV)
- *Goukovirus cumutoense*
  - Cumuto virus (CUMV)
- *Goukovirus gouleakoense*
  - Gouléako virus (GOLV)
- *Goukovirus yichangense*
  - Yíchāng insect virus (YcIV)

Horwuvirus
- *Horwuvirus fitzroyense*
  - Fitzroy Crossing tenui-like virus 1 (FCTenV1)
- *Horwuvirus solenopsidis*
  - Solenopsis invicta virus 14 (SINV14)
- *Horwuvirus wuhanense*
  - Wǔhàn horsefly virus (WhHV)

Hudivirus
- *Hudivirus muscae*
  - Húběi diptera virus 4 (HbDV4)

Hudovirus
- *Hudovirus lepidopteris*
  - Húběi lepidoptera virus 1 (HbLV1)

Ixovirus
- *Ixovirus heckscherense*
  - blacklegged tick virus 1 (BLTV1)
- *Ixovirus ixodis*
  - blacklegged tick virus 3 (BLTV3)
- *Ixovirus norvegiae*
  - Fairhair virus (FHAV)

Laulavirus
- *Laulavirus alphaviticulum*
  - grapevine associated cogu-like virus 2 (GaCLV2)
- *Laulavirus betaviticulum*
  - grapevine associated cogu-like virus 3 (GaCLV3)
- *Laulavirus gammaviticulum*
  - grapevine associated cogu-like virus 4 (GaCLV4)
- *Laulavirus laurelense*
  - Laurel Lake virus (LLV)
- *Laulavirus wardellense*
  - Wardell virus (WRDV)

Lentinuvirus
- *Lentinuvirus lentinulae*
  - Lentinula edodes negative-strand RNA virus 2 (LeNSRV2)

Mechlorovirus
- *Mechlorovirus cucumeris*
  - melon chlorotic spot virus (MeCSV)
- *Mechlorovirus ramuense*
  - Ramu stunt virus (RmSV)

Mobuvirus
- *Mobuvirus arnae*
  - Euproctis pseudoconspersa bunyavirus (EPBV)
- *Mobuvirus mothrae*
  - Mothra virus (MTHV)
- *Mobuvirus narangueense*
  - Ñarangue virus (NRGV)
- *Mobuvirus stephanocirci*
  - Browner virus (BRWV)

Phasivirus
- *Phasivirus baduense*
  - Badu virus (BADUV)
- *Phasivirus guadeloupeense*
  - Guadeloupe mosquito phasivirus (GMPV)
- *Phasivirus hubeiense*
  - Húběi diptera virus 5 (HbDV5)
- *Phasivirus parryense*
  - Parry's Creek phasivirus 1 (PCPhasV1)
- *Phasivirus phasiense*
  - Phasi Charoen-like virus (PCLV)
- *Phasivirus wuhanense*
  - Wǔhàn fly virus 1 (WhFV1)
- *Phasivirus wutaiense*
  - Wǔtái mosquito virus (WtMV)

Phlebovirus
- *Phlebovirus adanaense*
  - Adana virus (ADAV)
- *Phlebovirus aguacateense*
  - Aguacate virus (AGUV)
- *Phlebovirus alcubeense*
  - Alcube virus (ACBV)
- *Phlebovirus alenquerense*
  - Alenquer virus (ALEV)
- *Phlebovirus almendrasense*
  - Tres Almendras virus (TRAV)
- *Phlebovirus ambeense*
  - Ambé virus (ABEV)
- *Phlebovirus anhangaense*
  - Anhangá virus (ANHV)
- *Phlebovirus arumowotense*
  - Arumowot virus (AMTV)
- *Phlebovirus bogoriaense*
  - Bogoria virus (BGRV)
- *Phlebovirus buenaventuraense*
  - Buenaventura virus (BUEV)
- *Phlebovirus bujaruense*
  - Bujaru virus (BUJV)
- *Phlebovirus cacaoense*
  - Cacao virus (CACV)
- *Phlebovirus campanaense*
  - Campana virus (CMAV)
- *Phlebovirus candiruense*
  - Ariquemes virus (ARQV)
  - Candirú virus (CDUV)
  - Jacundá virus (JCNV)
  - Morumbi virus (MRBV; MRMBV)
  - Mucura virus (MCRV; MRAV)
  - Serra Norte virus (SRNV)
- *Phlebovirus chagresense*
  - Chagres virus (CHGV)
- *Phlebovirus claroense*
  - Rio Claro virus (RICV)
- *Phlebovirus cocleense*
  - Coclé virus (CCLV)
- *Phlebovirus corfouense*
  - Corfou virus (CFUV)
- *Phlebovirus dashliense*
  - Dāshlī virus (DASV)
- *Phlebovirus duraniaense*
  - Durania virus (DRNV)
- *Phlebovirus echarateense*
  - Echarate virus (ECHV)
- *Phlebovirus embossosense*
  - Embossos virus (EMBV)
- *Phlebovirus floriseense*
  - Saint-Floris virus (SAFV)
- *Phlebovirus gabekense*
  - Gabek forest virus (GFV)
- *Phlebovirus gloriaense*
  - La Gloria virus (LAGV)
- *Phlebovirus gordilense*
  - Gordil virus (GORV)
- *Phlebovirus hediense*
  - Hédī virus (HEDV)
- *Phlebovirus icoaraciense*
  - Icoaraci virus (ICOV)
- *Phlebovirus itaitubaense*
  - Itaituba virus (ITAV)
- *Phlebovirus itaporangaense*
  - Itaporanga virus (ITPV)
- *Phlebovirus ixcanalense*
  - Ixcanal virus (IXCV)
- *Phlebovirus karimabadense*
  - Karimabad virus (KARV)
- *Phlebovirus kiborgochense*
  - Kiborgoch virus (KBGV)
- *Phlebovirus leticiaense*
  - Leticia virus (LTCV)
- *Phlebovirus limboense*
  - frijoles virus (FRIV)
  - Joá virus (JOAV)
- *Phlebovirus maldonadoense*
  - Maldonado virus (MLOV)
- *Phlebovirus massiliaense*
  - Massilia virus (MASV)
- *Phlebovirus medjerdaense*
  - Medjerda Valley virus (MVV)
- *Phlebovirus monagritaense*
  - Mona Grita virus (MOGV)
- *Phlebovirus mukawaense*
  - Mukawa virus (MKWV)
- *Phlebovirus mungubaense*
  - Munguba virus (MUNV)
- *Phlebovirus napoliense*
  - Arrábida virus (ARRV)
  - Balkan virus (BALKV)
  - Fermo virus (FERV)
  - Granada virus (GRV; GRAV)
  - Saddaguia virus (SADV)
  - sandfly fever Naples virus (SFNV)
- *Phlebovirus niqueense*
  - Nique virus (NIQV)
- *Phlebovirus ntepesense*
  - Ntepes virus (NTPV)
- *Phlebovirus odrenisrouense*
  - Odrénisrou virus (ODRV)
- *Phlebovirus oriximinaense*
  - Oriximiná virus (ORXV)
- *Phlebovirus pantanalense*
  - viola virus (VIOV)
- *Phlebovirus penablancaense*
  - Peña Blanca virus (PEBV)
- *Phlebovirus penshurtense*
  - Penshurt virus (PEHV)
- *Phlebovirus perkerraense*
  - Perkerra virus (PKEV)
- *Phlebovirus puniqueense*
  - Punique virus (PUNV)
- *Phlebovirus riftense*
  - Rift Valley fever virus (RVFV)
- *Phlebovirus riograndense*
  - Rio Grande virus (RGV)
- *Phlebovirus salangaense*
  - Salanga virus (SLGV)
- *Phlebovirus salehabadense*
  - Adria virus (ADRV)
  - Arbia virus (ARBV)
  - Bregalaka virus (BREV)
  - Olbia virus (OLBV)
  - Salehabad virus (SALV)
  - Zaba virus (ZABAV)
- *Phlebovirus saloboense*
  - Salobo virus (SLBOV)
- *Phlebovirus siciliaense*
  - sandfly fever Sicilian virus (SFSV)
- *Phlebovirus taparaense*
  - Tapará virus (TPRV)
- *Phlebovirus tehranense*
  - Tehran virus (THEV)
- *Phlebovirus ticoense*
  - Tico virus (TICV)
- *Phlebovirus toroense*
  - Capira virus (CAPIV)
  - Punta Toro virus (PTV)
- *Phlebovirus torosense*
  - Toros virus (TORV)
- *Phlebovirus toscanaense*
  - Toscana virus (TOSV)
- *Phlebovirus turunaense*
  - Turuna virus (TUAV)
- *Phlebovirus uriuranaense*
  - Uriurana virus (URIV)
- *Phlebovirus urucuriense*
  - Urucuri virus (URUV)
- *Phlebovirus zerdaliense*
  - Zerdali virus (ZERV)

Pidchovirus
- *Pidchovirus pidgei*
  - Pidgey virus (PGYV)
- *Pidchovirus stethori*
  - coleopteran phenui-related virus 308 (CoPrV308)

Rubodvirus
- *Rubodvirus argentinaense*
  - grapevine Muscat rose virus (GMRV)
- *Rubodvirus armeniaense*
  - grapevine Garan dmak virus (GGDV)
- *Rubodvirus mali*
  - apple rubbery wood virus 1 (ARWV1)
- *Rubodvirus prosserense*
  - apple rubbery wood virus 2 (ARWV2)

Tanzavirus
- *Tanzavirus daressalaamense*
  - Dar es Salaam virus (DeSV)

Tenuivirus
- *Tenuivirus echinochloae*
  - Echinochloa hoja blanca virus (EHBV)
- *Tenuivirus eurotritici*
  - European wheat striate mosaic virus (EWSMV)
- *Tenuivirus festucae*
  - Festuca stripe-associated virus (FSaV)
- *Tenuivirus kwazuluense*
  - wheat yellows virus (WhYV)
- *Tenuivirus oryzabrevis*
  - rice grassy stunt virus (RGSV)
- *Tenuivirus oryzaclavatae*
  - rice stripe virus (RSV; RStV)
- *Tenuivirus oryzalbae*
  - rice hoja blanca virus (RHBV)
- *Tenuivirus persotritici*
  - Iranian wheat stripe virus (IWSV)
- *Tenuivirus pontaense*
  - wheat white spike virus (WWSV)
- *Tenuivirus urochloae*
  - Urochloa hoja blanca virus (UHBV)
- *Tenuivirus zeae*
  - maize stripe virus (MSV; MStV; MSpV)

Uukuvirus
- *Uukuvirus dabieshanense*
  - Dàbiéshān tick virus (DbsTV; DBTV; DTV)
- *Uukuvirus dermacentoris*
  - American dog tick virus (ADAV)
- *Uukuvirus grandarbaudense*
  - Grand Arbaud virus (GAV)
- *Uukuvirus hoplandense*
  - Pacific coast tick virus (PACV)
- *Uukuvirus huangpiense*
  - Huángpí tick virus 2 (HpTV2)
- *Uukuvirus kabutoense*
  - Kabuto mountain virus (KAMV)
- *Uukuvirus kaisodiense*
  - Kaisodi virus (KASDV)
- *Uukuvirus lihanense*
  - Lǐhán tick virus (LITV)
- *Uukuvirus macquariense*
  - Precarious Point virus (PPV)
- *Uukuvirus rukutamaense*
  - Rukutama virus (RUKV)
- *Uukuvirus schmidti*
  - Nile warbler virus (NIWV)
- *Uukuvirus silverwaterense*
  - Silverwater virus (SILV)
- *Uukuvirus tachengense*
  - Tǎchéng tick virus 2 (TcTV2)
- *Uukuvirus toyoense*
  - Tōyo virus (TOYOV)
- *Uukuvirus tyulenyense*
  - Zaliv Terpeniya virus (ZTV)
- *Uukuvirus uriae*
  - murre virus (MURV)
- *Uukuvirus uukuniemiense*
  - Chizé virus (CHZV)
  - Fin V 707 virus (FINV)
  - Uukuniemi virus (UUKV)
- *Uukuvirus yongjiaense*
  - Yǒngjiā tick virus (YONV)

Wenrivirus
- *Wenrivirus penaei*
  - Mourilyan virus (MoV)

Wupedeviridae

Wumivirus
- *Wumivirus millepedae*
  - Wǔhàn millipede virus 2 (WhMV2)

Insthoviricetes

Articulavirales

Amnoonviridae

Tilapinevirus
- *Tilapinevirus tilapiae*
  - tilapia lake virus (TiLV)

Orthomyxoviridae

Alphainfluenzavirus
- *Alphainfluenzavirus influenzae*
  - influenza A virus (FLUAV)

Betainfluenzavirus
- *Betainfluenzavirus influenzae*
  - influenza B virus (FLUBV)

Deltainfluenzavirus
- *Deltainfluenzavirus influenzae*
  - influenza D virus (FLUDV)

Gammainfluenzavirus
- *Gammainfluenzavirus influenzae*
  - influenza C virus (FLUCV)

Isavirus
- *Isavirus salaris*
  - infectious salmon anemia virus (ISAV)

Mykissvirus
- *Mykissvirus tructae*
  - rainbow trout orthomyxovirus (RbtOV)
  - steelhead trout orthomyxovirus (SttOV1)

Quaranjavirus
- *Quaranjavirus araguariense*
  - Araguari virus (ARAV)
- *Quaranjavirus chadense*
  - Lake Chad virus (LKCV)
- *Quaranjavirus johnstonense*
  - Johnston Atoll virus (JAV)
- *Quaranjavirus quaranfilense*
  - Quaranfil virus (QRFV)
- *Quaranjavirus tyulekense*
  - Tyulek virus (TLKV); Tjuloc virus
- *Quaranjavirus wellfleetense*
  - Wellfleet Bay virus (WFBV)

Sardinovirus
- *Sardinovirus pilchardi*
  - pilchard orthomyxovirus (POMV)

Thogotovirus
- *Thogotovirus bourbonense*
  - Bourbon virus (BRBV)
- *Thogotovirus dhoriense*
  - Dhori virus (DHOV)
- *Thogotovirus josense*
  - Jos virus (JOSV)
- *Thogotovirus ozense*
  - Oz virus (OZV)
- *Thogotovirus sinuense*
  - Sinu virus (SINUV)
- *Thogotovirus thailandense*
  - Thailand tick thogotovirus (TT-THOV)
- *Thogotovirus thogotoense*
  - Thogoto virus (THOV)
- *Thogotovirus upoluense*
  - Upolu virus (UPOV)

Unassigned

Tosoviridae

Fraservirus
- *Fraservirus testudinis*
  - turtle fraservirus 1 (TFV1)

Phylum
Subphylum
Class
Order
Family
Subfamily
Genus
Species
Virus (abbreviation)
